# The inclusion and impact of digital determinants of health in digital nutrition interventions for adolescents: a systematic review

**DOI:** 10.1093/heapro/daaf154

**Published:** 2025-09-29

**Authors:** Paris Mooney, Lai Ting Veronica Lam, Allyson R Todd, Stephanie R Partridge, Rebecca Raeside

**Affiliations:** Nutrition and Dietetics Group, Faculty of Medicine and Health, Susan Wakil School of Nursing and Midwifery, The University of Sydney, Level 8, Susan Wakil Health Building, Western Ave, Camperdown, NSW 2050, Australia; Nutrition and Dietetics Group, Faculty of Medicine and Health, Susan Wakil School of Nursing and Midwifery, The University of Sydney, Level 8, Susan Wakil Health Building, Western Ave, Camperdown, NSW 2050, Australia; Faculty of Medicine and Health, Susan Wakil School of Nursing and Midwifery, The University of Sydney, Level 8, Susan Wakil Health Building, Western Ave, Camperdown, NSW 2050, Australia; Charles Perkins Centre, The University of Sydney, Johns Hopkins Dr, Camperdown NSW 2050, Australia; Faculty of Medicine and Health, Susan Wakil School of Nursing and Midwifery, The University of Sydney, Level 8, Susan Wakil Health Building, Western Ave, Camperdown, NSW 2050, Australia; Charles Perkins Centre, The University of Sydney, Johns Hopkins Dr, Camperdown NSW 2050, Australia; Faculty of Medicine and Health, Susan Wakil School of Nursing and Midwifery, The University of Sydney, Level 8, Susan Wakil Health Building, Western Ave, Camperdown, NSW 2050, Australia; Charles Perkins Centre, The University of Sydney, Johns Hopkins Dr, Camperdown NSW 2050, Australia

**Keywords:** adolescent health, digital determinants of health, nutrition, digital health, health equity

## Abstract

Digital determinants of health (DDoH) is an emerging concept that captures domains relating to both digital health adoption and health equity. Digital transformations are reshaping many aspects of healthcare and health promotion, including how adolescent nutrition interventions are developed, delivered and utilized. As digital health interventions expand in popularity, it is crucial that they do not widen existing health disparities. This systematic review aimed to evaluate whether the DDoH are addressed in the development or delivery of digital nutrition interventions for adolescents, and whether this impacts access or use of these interventions to influence nutrition outcomes. Ten major electronic databases were searched and dual screened, capturing randomized controlled trials published from 2005 that aimed to improve nutrition outcomes through digital health interventions among adolescents 10–19 years. Primary outcome was objective or self-report change in nutrition intake or behaviours. DDoH assessment criteria were developed against nine pre-established dimensions. Study and intervention characteristics including information aligning with DDoH assessment criteria were extracted, and data synthesized in narrative format. Twenty articles representing 19 unique studies (13 246 participants) were identified, with 84% of studies conducted in high-income countries. All studies delivered interventions through mobile phone or computers and addressed at least one DDoH criteria. Affordability (100%) and usability (42%) were the most common DDoH criteria addressed. No studies successfully addressed all DDoH criteria. Therefore, we were unable to assess impact of addressing DDoH on adolescent nutrition outcomes. Overall, DDoH were inadequately addressed or reported in the development of digital nutrition interventions targeting adolescents.

Contribution to Health Promotion

- This review took a novel approach by developing assessment criteria to evaluate whether digital interventions aimed at improving adolescent nutrition addressed the digital determinants of health (DDoH).
- This review demonstrates the need to consider the DDoH in the design and delivery of digital health interventions to support equitable outcomes.
- The DDoH assessment criteria hold potential as a tool to evaluate and inform future digital health promotion efforts among adolescents.

## INTRODUCTION

Adolescence, as defined by the World Health Organization (WHO) encompasses individuals aged 10–19 (World Health Organization 2024a). This critical period of life is characterized by significant physical, emotional and cognitive development (National Academies of Sciences 2019). During this period, ∼15%–25% of adult height, up to 50% of adult weight and around 50% of adult bone mass are attained (Stang and Stotmeister 2017, World Health Organization 2024a). Other foundational health behaviours, such as diet, physical activity, social behaviours and emotional regulation are also established, which have long-term implications on overall health and wellbeing (Patton *et al*. 2016). Adolescence has previously been regarded as a relatively healthy stage of life (World Health Organization 2024a). However, due to rapid changes in environmental, social, commercial, and cultural factors over recent decades, today’s adolescents are becoming vulnerable to establish poor health behaviours (e.g. increased sedentary time, consumption of energy-dense, nutrient-poor foods). Global attention is being placed on the importance of investment to improve adolescent health and wellbeing, as the economic and social returns will be significant, estimated at USD $10 for every USD $1 spent (World Health Organization 2024b).

Adolescents currently face multiple heightened nutritional risks, including but not limited to, micronutrient deficiencies, food insecurity, malnutrition, poor-quality diets, and a growing obesity epidemic (Hargreaves *et al*. 2022). A 2020 multi-country study using 24-h dietary recall found most adolescents consumed less than the recommended fruits and vegetables, with a third of their intake comprising of ultra-processed foods (Fleming *et al*. 2020). Adolescents were also identified as having limited nutrition knowledge or skills to purchase and prepare healthy foods (Fleming *et al*. 2020). In addition, sedentary lifestyle and the widespread availability of energy-dense, nutrient-poor foods have significantly contributed to the global rise in adolescent obesity (Kerr *et al*. 2025). This is reflected in the increase in the prevalence of children and adolescents with overweight and obesity globally. From 1990 to 2021, the prevalence of overweight doubled, and obesity tripled, to a total of 493 million (Kerr *et al*. 2025). Reducing diet-related health risks has the potential to alleviate both the health and economic burdens, by lowering healthcare costs and reducing the demand on health services (Afshin *et al*. 2019, Partridge *et al*. 2024). Targeted health promotion interventions, education, and policies are needed to empower adolescents for improvement in dietary behaviours. However, it is well established that the social determinants of health influence health equity, with individuals from priority populations (e.g. culturally and linguistically diverse, socioeconomically disadvantaged) experiencing greater health inequities such as limited access to affordable healthcare, quality education, and food insecurity (Braveman *et al*. 2011). These social determinants influence dietary behaviours, dietary intake, food access, and food choices, which are common targets of nutrition interventions (Braveman *et al*. 2011 ). Thus, addressing the social determinants of health through health interventions is crucial to reduce health inequities. Without addressing these, interventions may be unintentionally creating additional barriers to accessing information, services, and education, making efforts to improve adolescent nutrition outcomes more challenging.

Digital health interventions hold potential to improve adolescent nutrition outcomes, yet the research to date has been unable to show consistent impact (Hargreaves *et al*. 2022). Prior research has examined effectiveness of digital health interventions to improve nutrition behaviours among adolescents, demonstrating that websites are effective for supporting behaviour change but this change was not sustained in the mid- to long- term (Rose *et al*. 2017). A recently published review also evaluated behaviour change techniques that were effective in digital dietary interventions among adolescents, finding that when personalized feedback and gamification were incorporated, there was high intervention adherence and improvements in dietary behaviours, yet long-term engagement was also a challenge (Melo *et al*. 2025). In addition, social media has been identified as a promising tool for nutrition interventions among adolescents, with a majority of studies having significant improvements in nutrition outcomes but engagement was variable (Chau *et al*. 2018, Kulandaivelu *et al*. 2023). To progress this field of research it is vital that research looks beyond engagement with digital health interventions. Research must examine implicit factors within the technology itself (e.g. personalization, accessibility) as well as the use and implementation of the technology, as these factors have potential to widen existing health disparities if they are not addressed.

The newly established digital determinants of health (DDoH) may be applied as a tool to determine whether digital health interventions are impacting health equity. DDoH refer to the design, use, and implementation of technological factors that may interact with sociodemographic disparities and health inequities to influence health outcomes and delivery of affordable, accessible, and quality care. It includes multiple dimensions such as data poverty and information asymmetry, ease of use, usability, interactivity, digital literacy, digital accessibility, digital affordability, algorithmic bias, and technology personalization (Chidambaram *et al*. 2024). Without consideration of these determinants, digital health interventions risk exacerbating existing health disparities, as they may fail to address the needs of all population groups. This in turn will impact upon the effectiveness of digital interventions. Consideration of the DDoH dimensions may provide a way to advance digital nutrition interventions for adolescents, ensuring that technology is able to be used by priority population groups. However, the impact of the DDoH among digital nutrition interventions for adolescents remains poorly understood.

To advance equity in digital health interventions, it is important to understand whether the DDoH have been considered and how these factors influence the effectiveness of interventions aimed at improving adolescent nutrition outcomes. This review aims to evaluate the effect of DDoH on digital interventions aiming to improve adolescents’ nutrition outcomes, and addresses these questions:

1. Have the DDoH been addressed in the development or delivery of adolescent digital nutrition interventions?
2. Does addressing the DDoH influence how adolescents’ access and use digital nutrition interventions and does that impact nutrition outcomes?

## METHODS

### Protocol and registration

This systematic review was conducted by following the Preferred Reporting Items for Systematic reviews and Meta-Analysis (PRISMA) statement guidelines (PRISMA 2020) (Supplementary Table S1). The review was registered in the International Prospective Register of Systematic Reviews (CRD42024582141), and there were no deviations from the registered protocol.

### Eligibility criteria

Eligibility criteria for study selection were formulated using the Population, Intervention, Comparison, Outcome, and Setting framework. Data from included studies were synthesized within the context of the DDoH (Chidambaram *et al*. 2024) as outlined below. Studies that were eligible met the following criteria:

#### Population

Adolescents, defined by the WHO as 10–19 years old (World Health Organization 2024a), of all genders and any demographic background.

#### Intervention

Delivered via digital health interventions of any duration. Digital health refers to the utilization of information and communication technologies to support health and healthcare-related activities (World Health Organization 2019). This includes both eHealth and mobile health, including telehealth, email, mobile phone applications, text messaging, websites, smart watches, activity trackers, email, and personal digital assistant use. Emerging areas of digital health such as social media, artificial intelligence (AI), and digital games were also eligible for inclusion.

#### Comparator

Studies need to have a comparator group receiving standard care or a control intervention.

#### Outcomes

Primary outcome of interest was objective or self-reported change in adolescents’ nutrition intake or behaviours.

#### Setting

All study settings were included, such as digital-only, community, home, school-based or health care. Only randomized controlled trials (RCTs) were eligible for inclusion and only studies published after 2005 were considered. The cut-off date of 2005 was selected as the current generation of adolescents (‘Generation Z’) appeared in the population after 1995, and the oldest of this generation were 10 years old in 2005 (Oxford English Dictionary 2025).

### Information sources and search strategy

Ten electronic databases (Pre-Medline, Medline, Cochrane, Cochrane Central Register of Controlled Trials, Embase, CINAHL, AMED, Informit, Scopus, and Web of Science) were systematically searched by two authors (P.M. and V.L.) until 26 August 2024. Database searches were developed drawing from relevant literature (Partridge *et al*. 2024, Raeside *et al*. 2024). Search terms were combined from three main concepts: (i) adolescents, (ii) digital health, and (iii) nutrition. Search terms were combined using AND/OR operators and included combinations, truncations and synonyms. Database RCT filters were applied (where appropriate) to maximize the results of RCTs and limits were set to only identify papers published from 2005 to current. Full search strategies for each database and screenshots of search results are available in Supplementary Tables S2–S7 and Supplementary Figures S1–S10.

### Study selection

Due to the volume of databases searched, two authors (P.M. and V.L.) equally divided electronic databases and searched five each. Both authors identified articles using the search strategy and uploaded references to Endnote citation manager (version 21). Endnote citation manager was used to remove duplicates and articles were uploaded to Covidence (Veritas Health Innovation Ltd, Melbourne, Australia). Covidence further removed duplicates and studies that were not RCTs before the title abstract screening began. Following the Cochrane Handbook of Systematic Reviews and the PRISMA statement for study selection (PRISMA 2020, Higgins *et al*. 2024), both authors (P.M. and V.L.) independently screened titles and abstracts and removed ineligible studies. Next, full-text articles were screened by two authors (P.M. and V.L.) for eligibility against the pre-determined inclusion and exclusion criteria. Conflicts or disagreements were reviewed and resolved with the research team (R.R., A.R.T., and S.R.P.).

### Data extraction

For studies meeting the inclusion criteria, information was extracted into pre-designed data extraction tables in Microsoft Word. Two authors (P.M. and V.L.) independently extracted the data, and the research team (R.R. and A.R.T.) cross-checked all included articles for accuracy. Extracted data include participant characteristics summarized by the Cochrane PROGRESS-Plus Framework (O'Neill *et al*. 2014) This included place of residence, race/ethnicity/culture/language, occupation, gender/sex, religion, education, socioeconomic status, social capital, and other equity factors (e.g. age disability). Countries income status was categorized according to the World Bank classification (The World Bank 2025). Intervention characteristics were summarized and extracted by modality, intervention details, exposure, control, duration, DDoH criteria addressed and nutritional outcomes (intake and/or behaviours e.g. fruit and vegetable intake, adherence to food groups, consumption of discretionary food, and dietary data collection methods). Any missing data of demographics was excluded from calculation. Missing, incomplete or unclear data were clarified where possible by contacting corresponding authors of included studies.

### Data synthesis and analysis—development of digital determinants of health assessment criteria

The DDoH dimensions were previously developed by (Chidambaram *et al*. 2024) and includes data poverty and information asymmetry, ease of use, usability, interactivity, digital literacy, digital accessibility, digital affordability, algorithmic bias and technology personalization. To develop the DDoH assessment criteria, a definition for each dimension was first established through searching relevant supporting literature by the authors (P.M. and V.L.) (Adomavicius and Tuzhilin 2006, Cover 2016, Guidelines Review Committee 2019, Lattimore *et al*. 2020, McLean *et al*. 2020, Barraket 2021, Clinical Trials Transformation Initiative 2021, Hargreaves *et al*. 2022, Jahnel *et al*. 2022, Fraser *et al*. 2023, Holly *et al*. 2023, Park and Kim 2023, Chidambaram *et al*. 2024, University of Florida Health 2024). Next, three categories were developed to assess whether each DDoH criteria were ‘addressed’, ‘somewhat addressed’ or ‘not addressed’. These were developed and piloted prior to data extraction by both authors (P.M., V.L.) in collaboration with the research team (R.R., A.R.T., S.R.P.). Table 1 provides the DDoH assessment criteria, including definitions for the nine DDoH dimensions.

**Table 1.** Definitions and criteria to assess digital determinants of health within digital health interventions.

Digital determinant of health	Definition	Addressed	Somewhat addressed	Not addressed
Data poverty and information asymmetry	Health data poverty is the inability for individuals, groups, or populations to benefit from a discovery or innovation due to insufficient data that are adequately representative (Chidambaram *et al*. 2024). A key factor in determining the effectiveness of digital health and especially machine learning models is data completeness and accuracy, including how faithfully it represents all communities and individuals (Fraser *et al*. 2023).	Intervention is developed from special research on underserved groups. Information is given across multiple platforms or to multiple populations with the same information without advantaging or biasing any topic or population.	Intervention adjusted for minority groups characteristics, e.g. diet habits, requirements/study population has targeted underserved groups, but intervention not specifically developed upon their characteristics.	Intervention is directly adapted from other studies, with no consideration of one's own study population.
Usability	The degree to which the participant population is willing and able to interact with the digital technology as required for its effective use (Clinical Trials Transformation Initiative 2021).	Participants received detailed instructions/continuous support for receiving interventions. Intervention is designed to be engaging specifically for the study population.	Intervention has been designed to be easily adapted by participants.	No instructions are provided to participants before/throughout the intervention period.
Perceived usefulness	The extent to which people find a technology to be a facilitator of their work (Park and Kim 2023).	Intervention is designed to fit specifically for the population needs/preference.	Intervention involves some design that increases engagement, but not directly to application of intervention.	Intervention was adapted from other studies that were not applied to the study population with similar characteristics e.g. adult intervention applied to adolescent’s study.
Interactivity	The ability for a digital intervention to interact with the participant or for participants to receive live help or feedback (Cover 2016).	Participants can interact with each other. Participants can interact through online forums, discussion groups and messaging. Participants receive an interactive component to a digital intervention such as through gaming or website use.	Only some participants can interact with others.	Participants solely carry out intervention.
Digital literacy	Digital literacy is the ability and skill of individuals to understand and use digital technology safely and effectively (McLean *et al*. 2020).	Participants are provided with training on how to use the digital intervention. Participants are provided ongoing support throughout the duration of the study. Participants are able to contact support throughout the duration of the study.	Participants can contact support throughout the duration of the study but not training on how to use the digital intervention.	Participants are not given guidance or ongoing help on how to navigate and use the digital intervention.
Accessibility	Digital accessibility refers to the use of digital technologies by all populations (University of Florida Health 2024).	Specialized software or digital interventions that cater for individuals with varying needs such as but not limited to vision or hearing impairment, disability or individuals with low socioeconomic status. Digital interventions that provide free internet and devices to individuals that do not have access to a mobile phone, computer or internet.	Digital interventions that provide devices, training or specialized equipment to some individuals and exclude others.	Digital interventions that exclude populations of people who cannot access the intervention.
Affordability	Digital affordability refers to the ability to afford digital technologies with access cost covered (Barraket 2021).	Participants are given mobile phones or internet access to access the digital intervention. Participants are given free access to all aspects of the digital intervention. Participants are financially compensated.	Participants are given free access to websites, games or technology but required a mobile phone or internet access to be included in the study. Participants are given free access to some parts of the digital intervention but were required to pay for others.	Participants require a mobile phone or internet to be included in the study and are not given a mobile phone or internet access if they cannot afford it. Participants are required to pay for the digital intervention.
Algorithmic bias	Algorithmic bias refers to the systemic errors in a technological system that create unfair outcomes, biasing a particular population or topic (Lattimore *et al*. 2020).	Use of artificial intelligence to develop programmes for minorities within populations. Even distribution of information and data.	Algorithmic bias was used to personalize digital interventions to a certain population but is not adaptable to other populations.	Algorithmic bias in word associations, online ads, facial recognition technology, incomplete training data or reporting bias. Recruitment tools favouring gender, sex, ethnicity, age, or socioeconomic status. Systematic errors and repeatable behaviours in a computer system that create an unfair advantage for one category over another.
Technology personalization	Technology personalization is the personalization of digital technologies to individuals (Adomavicius and Tuzhilin 2006).	Digital interventions that give participants personalized feedback, programmes, training or advice. Digital interventions that are designed upon the study of population characteristics. Digital interventions that are personalized to the participant population.	Digital interventions that give generalized digital interventions with personalized feedback after a specific duration.	Digital interventions that do not give any personalized feedback, programmes, training or advice.

For example, usability (defined as the degree to which the participant population is willing and able to interact with the digital technology as required for its effective use) was determined to be addressed if the participants received detailed instructions, continuous support on how to use the interventions or if the intervention was specifically designed to be engaging for the chosen study population. Usability was considered somewhat addressed if the intervention was designed to be easily adapted by participants and not addressed if the participants received no instructions before or throughout the intervention period.

Each DDoH criteria influences the adoption of digital health interventions and health equity within population groups. Therefore, addressing as many applicable criteria should be the goal, and addressing all DDoH criteria is best practice. For the purposes of this study, all applicable criteria must have been addressed or somewhat addressed to move on to assessing the impact of DDoH on nutrition outcomes. A narrative synthesis and simple descriptive statistics determined the frequency of studies meeting each criteria, the adequacy of reporting of the criteria across included studies, and whether nutrition outcomes were influenced by addressing the DDoH.

### Risk of bias assessment

The Cochrane Risk of Bias (RoB) 2 tool (Cochrane Methods Bias 2024) was used to assess the risk of bias of the included studies which were independently evaluated by two authors (P.M. and V.L.). Any discrepancies were reviewed by the research team (R.R., A.R.T., and S.R.P.). The risk of bias domains included randomization process, deviations from the intended interventions, missing outcome data, measurement of the outcome and selection of the reported result. Included articles were then judged ‘low’, ‘some concerns’ or ‘high’ risk of bias (Cochrane Methods Bias 2024).

## RESULTS

### Study selection

**Figure 1.**
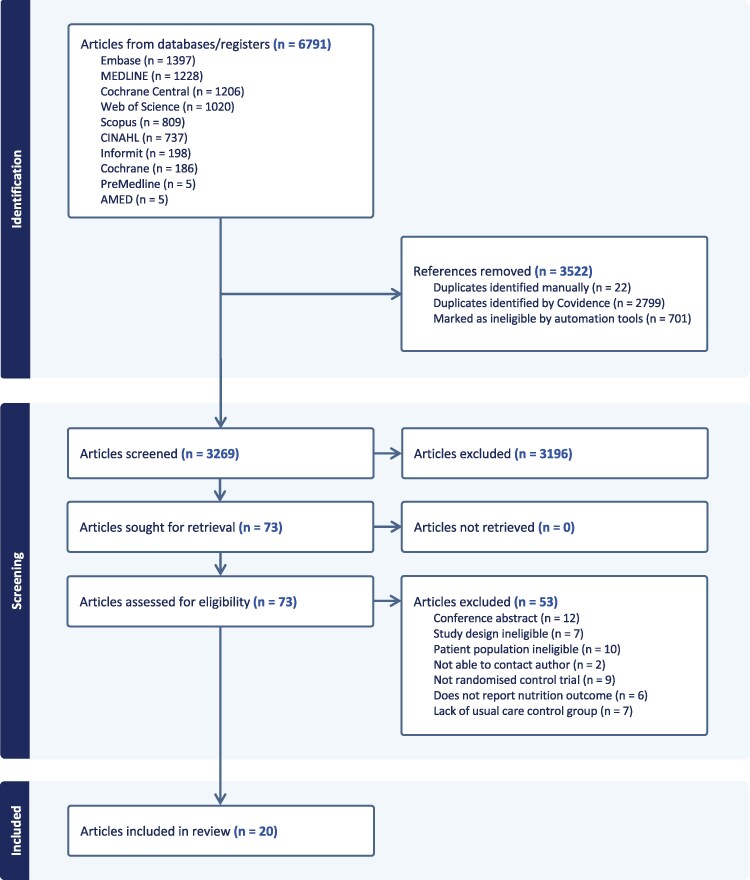
PRISMA flow diagram.

The searches retrieved 6791 articles from the electronic databases (Fig. 1). After removing duplicates, 3269 articles were screened by title and abstract and 3196 were excluded. A total of 73 full-text articles were assessed for eligibility and 53 were excluded with reasons available in Supplementary Information (Supplementary Table S8). Twenty articles representing 19 unique studies met our inclusion criteria and were included in the final synthesis, representing 13 246 participants.

### Study characteristics

Studies were conducted from 2006 to 2024. Out of 19 unique studies, six (32%) were conducted in the United States of America (Doyle *et al*. 2008, Jones *et al*. 2008, Patrick *et al*. 2013, Nollen *et al*. 2014, Chen *et al*. 2019, Wambach *et al*. 2022) and one (5%) was conducted in each of the following countries: Australia (Heinicke *et al*. 2007), Spain (Manzano-Felipe *et al*. 2024), Finland (Heikkila *et al*. 2019), Saudi Arabia (Shatwan *et al*. 2023), Denmark (Bjerregaard *et al*. 2024), Thailand (Likhitweerawong *et al*. 2021), Germany (van Egmond-Fröhlich *et al*. 2006), Canada (Tugault-Lafleur *et al*. 2023), Iran (Ghadam *et al*. 2023), Belgium (Vermeiren *et al*. 2021), India (Sivasamy *et al*. 2020), and Italy (Carfora *et al*. 2016). One study (5%) was conducted across Spain and Mexico (Lana *et al*. 2014). Further, 16 studies (84%) were conducted in high-income countries, two (10%) in upper-middle-income countries and one (5%) in a lower-middle-income country (The World Bank 2025). Sample size of the studies ranged from 40 to 7890 participants and the mean age (SD) was 14.1 (2.05), with four studies not included in the mean calculation and eight studies not included in the SD calculation as it was not reported. Ten studies (53%) recruited participants from schools (Heinicke *et al*. 2007, Jones *et al*. 2008, Lana *et al*. 2014, Nollen *et al*. 2014, Carfora *et al*. 2016, Sivasamy *et al*. 2020, Likhitweerawong *et al*. 2021, Ghadam *et al*. 2023, Shatwan *et al*. 2023, Manzano-Felipe *et al*. 2024), four studies (21%) recruited participants from community health centres including general and specialist practices (Patrick *et al*. 2013, Chen *et al*. 2019, Wambach *et al*. 2022, Bjerregaard *et al*. 2024), three studies (16%) recruited participants from hospitals as inpatients (van Egmond-Fröhlich *et al*. 2006, Tugault-Lafleur *et al*. 2023, Vermeiren *et al*. 2021), and one study (5%) each recruited from community centres and school (Doyle *et al*. 2008) and sports clubs (Heikkila *et al*. 2019). Most studies (79%) included participants of both sexes whilst four (21%) recruited females only (Heinicke *et al*. 2007. Nollen *et al*. 2014, Wambach *et al*. 2022, Ghadam *et al*. 2023). Four studies (21%) reported family situation and parents’ level of education (Heinicke *et al*. 2007, Doyle *et al*. 2008, Shatwan *et al*. 2023, Tugault-Lafleur *et al*. 2023). Six studies (32%) reported family income (Heinicke *et al*. 2007, Patrick *et al*. 2013, Nollen *et al*. 2014. Wambach *et al*. 2022, Shatwan *et al*. 2023, Tugault-Lafleur *et al*. 2023). Full study characteristics are available in Supplementary Table S9.

### Intervention characteristics and effectiveness

Fifteen of the 19 interventions (79%) were delivered completely through digital modalities (Heinicke *et al*. 2007, Doyle *et al*. 2008, Jones *et al*. 2008, Lana *et al*. 2014, Nollen *et al*. 2014, Carfora *et al*. 2016, Chen *et al*. 2019 , Sivasamy *et al*. 2020, Likhitweerawong *et al*. 2021, Vermeiren *et al*. 2021, Wambach *et al*. 2022, Ghadam *et al*. 2023, Shatwan *et al*. 2023, Tugault-Lafleur *et al*. 2023, Bjerregaard *et al*. 2024). Four studies (21%) applied multicomponent interventions (van Egmond-Fröhlich *et al*. 2006, Patrick *et al*. 2013, Heikkila *et al*. 2019, Manzano-Felipe *et al*. 2024). These other intervention components were mostly delivered in school settings, including school-based education, outpatient follow-ups and school sports programmes. Among digital modalities of delivery, all studies delivered digital interventions through mobile phone or computer access. Online programmes or games were applied in ten studies (53%) (Heinicke *et al*. 2007, Doyle *et al*. 2008, Jones *et al*. 2008, Patrick *et al*. 2013, Lana *et al*. 2014, Nollen *et al*. 2014, Chen *et al*. 2019, Vermeiren *et al*. 2021, Wambach *et al*. 2022, Ghadam *et al*. 2023), with the other nine (47%) using SMS, apps or phone-call follow-up (van Egmond-Fröhlich *et al*. 2006, Carfora *et al*. 2016, Heikkila *et al*. 2019, Sivasamy *et al*. 2020, Likhitweerawong *et al*. 2021, Shatwan *et al*. 2023, Tugault-Lafleur *et al*. 2023, Bjerregaard *et al*. 2024, Manzano-Felipe *et al*. 2024). Thirteen studies (68%) reported nutrition outcomes by measuring food, beverage, or nutrient intake (van Egmond-Fröhlich *et al*. 2006, Lana *et al*. 2014, Nollen *et al*. 2014, Carfora *et al*. 2016, Chen *et al*. 2019, Heikkila *et al*. 2019, Sivasamy *et al*. 2020, Likhitweerawong *et al*. 2021, Ghadam *et al*. 2023, Shatwan *et al*. 2023, Tugault-Lafleur *et al*. 2023, Wambach *et al*. 2022, Bjerregaard *et al*. 2024); one (5%) measured adherence to diet (Manzano-Felipe *et al*. 2024); and five others (26%) assessed nutrition behaviours (Heinicke *et al*. 2007, Doyle *et al*. 2008, Jones *et al*. 2008, Patrick *et al*. 2013, Vermeiren *et al*. 2021). Five studies (26%) reported positive significant differences in nutrition outcomes between intervention and control groups (Heinicke *et al*. 2007, Jones *et al*. 2008, Carfora *et al*. 2016, Likhitweerawong *et al*. 2021, Ghadam *et al*. 2023). Among studies with significant differences between intervention and control group, three intervened with mobile phones (app and/or text messaging) and two intervened with online programmes (Heinicke *et al*. 2007, Jones *et al*. 2008, Carfora *et al*. 2016, Likhitweerawong *et al*. 2021, Ghadam *et al*. 2023), where participants learnt nutrition knowledge and achieved behavioural change. Full intervention characteristics are available in Supplementary Table S10.

### Digital determinants of health assessment criteria

All 19 studies were assessed against nine DDoH assessment criteria (171 assessments total). 29.8% (51/171) of all criteria were addressed, 4.1% (7/171) were somewhat addressed, 55.0% (94/171) were not addressed, and 11.1% (19/171) were not applicable. Overall, none of the studies successfully addressed all DDoH assessment criteria and only six studies (32%) have somewhat addressed or addressed four or more criteria in their interventions (Heinicke *et al*. 2007, Doyle *et al*. 2008, Patrick *et al*. 2013, Nollen *et al*. 2014, Wambach *et al*. 2022, Tugault-Lafleur *et al*. 2023). All studies addressed at least one assessment criteria. An overview is presented in Table 2; all criteria are expanded on below.

**Table 2.** Overview of included studies addressing the digital determinants of health (*n* = 19).

	Digital determinants of health								
Author, year, country	Data poverty and information asymmetry	Usability	Perceived usefulness	Interactivity	Digital literacy	Accessibility	Affordability	Algorithmic bias	Technology personalization
Bjerregaard *et al*. (2024) Denmark	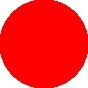	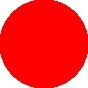	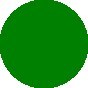	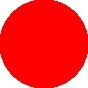	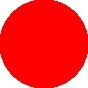	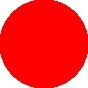	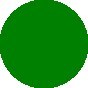	N/A	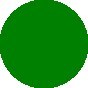
Carfora *et al*. (2016) Italy	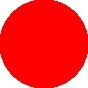	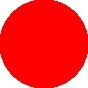	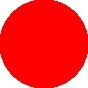	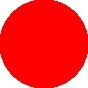	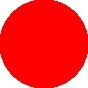	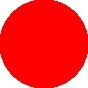	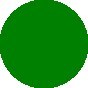	N/A	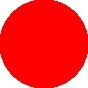
Chen et al. (2017) and Chen *et al*. (2019) USA	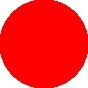	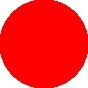	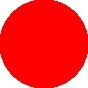	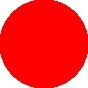	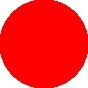	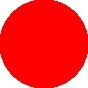	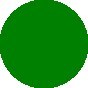	N/A	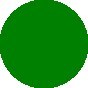
Doyle *et al*. (2008) USA	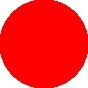	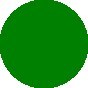	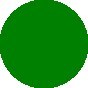	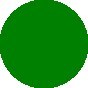	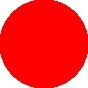	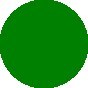	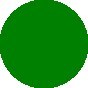	N/A	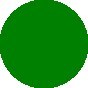
Ghadam *et al.* (2023) Iran	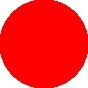	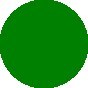	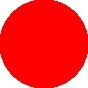	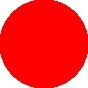	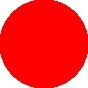	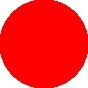	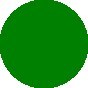	N/A	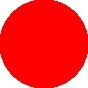
Heikkila *et al*. (2019) Finland	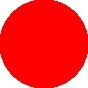	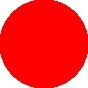	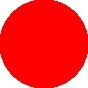	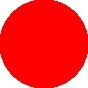	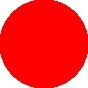	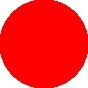	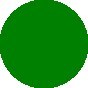	N/A	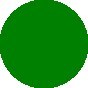
Heinicke *et al*. (2007) Australia	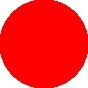	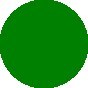	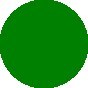	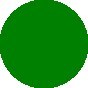	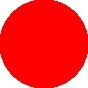	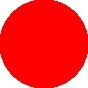	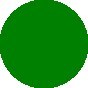	N/A	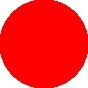
Jones *et al*. (2008) USA	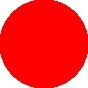	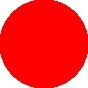	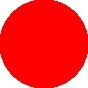	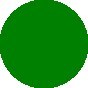	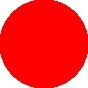	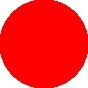	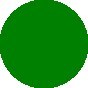	N/A	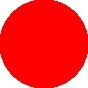
Lana *et al.* (2014) Spain and Mexico	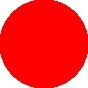	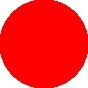	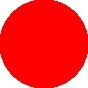	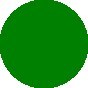	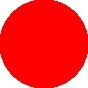	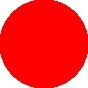	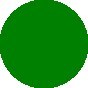	N/A	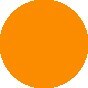
Likhitweerawong *et al*. (2021) Thailand	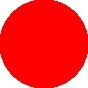	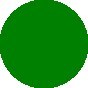	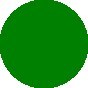	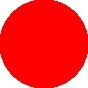	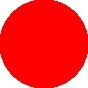	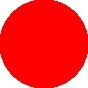	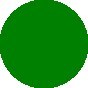	N/A	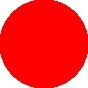
Manzano-Felipe *et al*. (2024) Spain	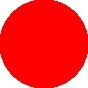	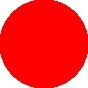	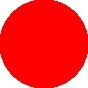	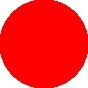	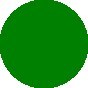	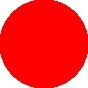	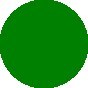	N/A	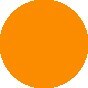
Nollen *et al*. (2014) USA	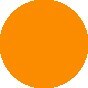	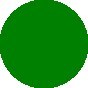	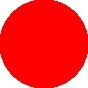	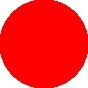	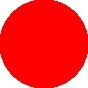	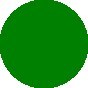	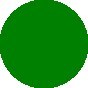	N/A	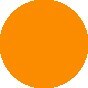
Patrick *et al*. (2013) USA	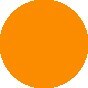	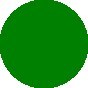	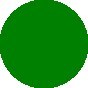	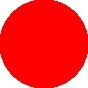	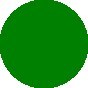	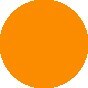	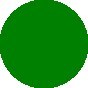	N/A	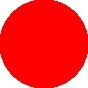
Shatwan *et al*. (2023) Saudi Arabia	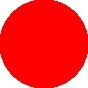	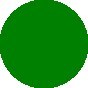	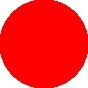	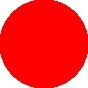	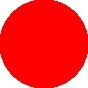	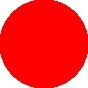	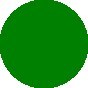	N/A	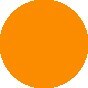
Sivasamy *et al*. (2020) India	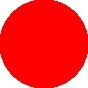	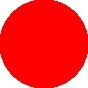	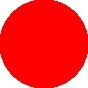	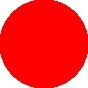	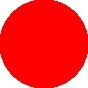	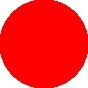	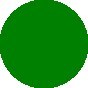	N/A	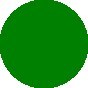
Tugault-Lafleur *et al*. (2023) Canada	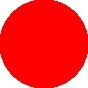	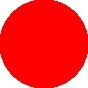	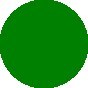	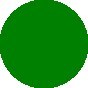	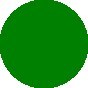	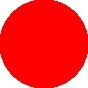	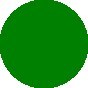	N/A	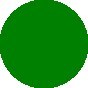
van Egmond-Fröhlich *et al*. (2006) Germany	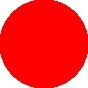	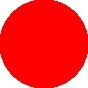	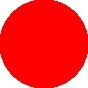	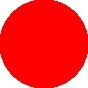	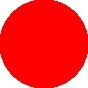	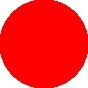	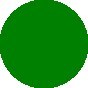	N/A	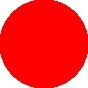
Vermeiren *et al* (2021) Belgium	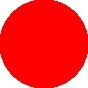	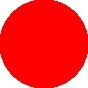	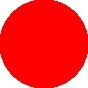	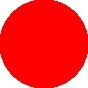	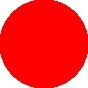	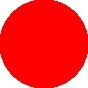	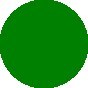	N/A	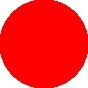
Wambach *et al*. (2022) USA	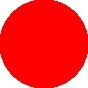	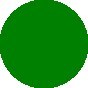	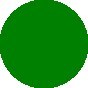	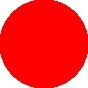	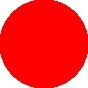	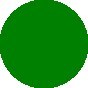	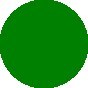	N/A	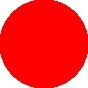

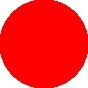
  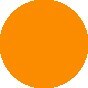  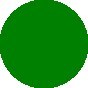

Key: Red = not addressed/not reported.

Orange = somewhat addressed.

Green = addressed.

N/A, not applicable.

#### Data poverty and information asymmetry

Only two studies (11%) somewhat addressed data poverty and information asymmetry (Patrick *et al*. 2013 , Nollen *et al*. 2014). They somewhat considered this digital determinant criteria by targeting the intervention to economically and ethnically diverse populations, but the intervention was not developed with specific adaptations to these populations.

#### Usability

Eight studies (42%) addressed usability (Heinicke *et al*. 2007, Doyle *et al*. 2008, Patrick *et al*. 2013, Nollen *et al*. 2014, Likhitweerawong *et al*. 2021, Wambach *et al*. 2022, Ghadam *et al*. 2023, Shatwan *et al*. 2023), through providing digital technology support sessions, pre-intervention orientations or training, trouble-shooting information or user manuals, or regular check-ups during the intervention period.

#### Perceived usefulness

Seven studies (37%) addressed perceived usefulness (Heinicke *et al*. 2007, Doyle *et al*. 2008, Patrick *et al*. 2013, Likhitweerawong *et al*. 2021, Wambach *et al*. 2022, Tugault-Lafleur *et al*. 2023, Bjerregaard *et al*. 2024). Five of them (26%) addressed this through designing the intervention upon participants’ characteristics or preferences (Heinicke *et al*. 2007, Doyle *et al*. 2008, Likhitweerawong *et al*. 2021, Wambach *et al*. 2022, Bjerregaard *et al*. 2024). Two of the studies (11%) were pre-tested with adolescents (Patrick *et al*. 2013, Tugault-Lafleur *et al*. 2023). Heinicke (Heinicke *et al*. 2007) conducted a qualitative evaluation of the intervention method before commencing the RCT to ensure perceived usefulness to participants.

#### Interactivity

Interactivity was addressed in five studies (26%) (Heinicke *et al*. 2007, Doyle *et al*. 2008, Jones *et al*. 2008, Lana *et al*. 2014, Tugault-Lafleur *et al*. 2023). They all included discussion forums, group sessions or peer-interactive chatrooms, which provided a space for participants to influence one another, gain peer support from sharing and responding to each other’s concerns. Three of the studies (16%) (Heinicke *et al*. 2007, Jones *et al*. 2008, Tugault-Lafleur *et al*. 2023) also aided participants with a research assistant or trained therapist who moderated chatrooms or appointments. In Tugault-Lafleur’s study (Tugault-Lafleur *et al*. 2023), research participants also received in-app text messages from a live coach and had access to an optional web-based appointment for further support.

#### Affordability

All studies provided free access to the digital intervention addressing affordability. However, four of them could only address the affordability criteria (van Egmond-Fröhlich *et al*. 2006, Carfora *et al*. 2016, Chen *et al*. 2019, Vermeiren *et al*. 2021). More than half (11 studies, 58%) excluded individuals from the study if they did not have access to a mobile phone, laptop, or internet (Heinicke *et al*. 2007, Doyle *et al*. 2008, Patrick *et al*. 2013, Carfora *et al*. 2016, Chen *et al*. 2019, Sivasamy *et al*. 2020, Likhitweerawong *et al*. 2021, Ghadam *et al*. 2023, Shatwan *et al*. 2023, Bjerregaard *et al*. 2024, Manzano-Felipe *et al*. 2024).

#### Digital literacy

A total of three studies (16%) addressed digital literacy by providing training or designing digital interventions for adolescent populations to ensure that they understood and had the skills to be able to use the technology (Patrick *et al*. 2013, Tugault-Lafleur *et al*. 2023, Manzano-Felipe *et al*. 2024). One study (5%) designed different versions of the digital intervention to suit different levels of digital literacy (Tugault-Lafleur *et al*. 2023). One study (5%) also pre-tested their intervention to ensure the level of understanding of concepts was appropriate to adolescent populations (Patrick *et al*. 2013).

#### Accessibility

Four studies (21%) addressed or somewhat addressed accessibility (Doyle *et al*. 2008 , Patrick *et al*. 2013, Nollen *et al*. 2014, Wambach *et al*. 2022). Two of these studies (11%) addressed this determinant through providing participants with a handheld device. For example, participants were provided with an iPad mini to access the intervention (Nollen *et al*. 2014, Wambach *et al*. 2022) and one study (5%) allowed participants to use school and public computers to access the intervention (Doyle *et al*. 2008). One study (5%) somewhat addressed accessibility by providing internet access at school but required participants to own a functioning mobile phone to be eligible for the study (Patrick *et al*. 2013). The majority of studies (11, 58%) did not address this as they reported that it was a requirement for participants to have access to a mobile phone, computer or internet to be included in the study therefore decreasing access to the intervention (Heinicke *et al*. 2007, Doyle *et al*. 2008, Patrick *et al*. 2013, Carfora *et al*. 2016, Chen *et al*. 2019, Sivasamy *et al*. 2020, Likhitweerawong *et al*. 2021 , Ghadam *et al*. 2023, Shatwan *et al*. 2023, Bjerregaard *et al*. 2024, Manzano-Felipe *et al*. 2024). Six studies (32%) also excluded individuals who had hearing or vision difficulties, disability, or were unable to speak English (Doyle *et al*. 2008, Likhitweerawong *et al*. 2021 , Shatwan *et al*. 2023, Tugault-Lafleur *et al*. 2023, Bjerregaard *et al*. 2024, Manzano-Felipe *et al*. 2024).

#### Algorithmic bias

Algorithmic bias was not applicable in any of the included studies as all interventions did not use algorithms or AI.

#### Technology personalization

Six studies (32%) addressed this domain by giving participants personalized feedback or interventions (Heinicke *et al*. 2007, Doyle *et al*. 2008, Chen *et al*. 2019, Sivasamy *et al*. 2020, Tugault-Lafleur *et al*. 2023, Bjerregaard *et al*. 2024). In Bjerregaard’s study (Bjerregaard *et al*. 2024), educational messages were sent according to participants’ individual dietary habit risk levels, which were evaluated and stratified prior to the intervention phase. Additionally, four studies (21%) have somewhat addressed this determinant. Study groups either received general interventions with personalized feedback, or intervention tools were designed upon certain study population characteristics, but not personalized for each participant individually (Lana *et al*. 2014, Nollen *et al*. 2014, Shatwan *et al*. 2023, Manzano-Felipe *et al*. 2024).

### Risk of bias

Overall, nine studies (47%) had a high risk of bias (van *et al*. 2006, Doyle *et al*. 2008, Jones *et al*. 2008, Lana *et al*. 2014, Sivasamy *et al*. 2020, Vermeiren *et al*. 2021, Wambach *et al*. 2022, Bjerregaard *et al*. 2024, Manzano-Felipe *et al*. 2024). Four of the studies were judged high risk of bias in the domain ‘missing outcome data’, generally due to analysis methods which did not account for missing data (e.g. intention to treat) or a lack of reporting of reasons that participants dropped out of the trial, thus a judgement could not be made on whether the missing data would impact trial outcomes (Doyle *et al*. 2008, Lana *et al*. 2014, Sivasamy *et al*. 2020, Vermeiren *et al*. 2021). Two studies were judged high risk of bias in the ‘measurement of the outcome’ domain (Wambach *et al*. 2022, Bjerregaard *et al*. 2024). In digital health studies, it can be difficult to blind participants to intervention assignment, and they are often also completing self-reported outcome measures which can introduce bias. Overall, many of the studies were poorly reported, or not reported according to CONSORT guidelines. Full results are available in Supplementary Table S11.

## DISCUSSION

This systematic review addresses a significant gap in the current literature, specifically in evaluating the DDoH in the development and delivery of adolescent digital nutrition interventions. Further, exploring if addressing the DDoH has an impact on the nutrition outcomes of these interventions. To our knowledge, no other published systematic reviews have addressed the impact of addressing the DDoH on nutrition outcomes for adolescents. This review found that the DDoH are significantly under reported or explored in the development of adolescent digital nutrition interventions. The lack of reporting or assessment of these limited the ability to assess the impact of the DDoH on adolescent nutrition outcomes. Therefore, it is unclear whether positive nutrition outcomes can be attributed to addressing DDoH or if improvements are due to external factors. Further, none of the included studies successfully addressed all DDoH criteria when designing and implementing their nutrition interventions. These findings demonstrate the need to integrate the DDoH into the design and delivery of digital health interventions, supporting not only their adoption but also fostering equitable access to digital health services. Systematic reporting of the DDoH should be applied to provide valuable insights for public health and healthcare sectors on how these determinants interconnect with intervention effectiveness, access, and further nutrition outcomes. Addressing this gap is crucial for advancing digital health equity and improving health outcomes for adolescents.

No studies in this review addressed all DDoH assessment criteria. The failure of digital health interventions to adequately address the DDoH has potential to exacerbate digital health inequities, highlighting the need for the DDoH assessment criteria. The newly developed assessment criteria outline how interventions can address all nine DDoH dimensions to enhance equity and adoption for future intervention development. It is essential for future research, as it provides a standardized criteria for evaluating the integration of the DDoH and offers strategies for enhancing digital health equity and adoption across populations. Our assessment criteria represent a unique contribution to the literature; however, we recognize that these have been developed to assess adolescent nutrition interventions and there is a need to expand these and apply them to other population groups and digital modality of interventions to expand definitions in the future. Regardless, the assessment criteria may help direct future development of digital health interventions to improve the nutritional outcomes of adolescents and in turn, improve overall health burden.

Previous studies have used the Digital Health Equity Framework developed by Richardson *et al*. to assess equity of digital health interventions (Richardson *et al*. 2022). Adapted from the National Institute on Minority Health and Health Disparities Research Framework (Alvidrez *et al*. 2019), it examines four levels of influence in the digital environment across individual, interpersonal, community, and societal levels. A previous study assessed digital health equity within adolescent obesity and management interventions, yet found inadequate reporting of digital health equity across all four levels of influence as interventions mostly targeted the individual level (Partridge *et al*. 2024). As most digital adolescent nutrition interventions also target the individual level, our team have developed the DDoH assessment criteria. This newly developed assessment criteria are more relevant for assessing interventions which target the individual level. This is demonstrated through differences in the number of criteria addressed between the studies, e.g. 82% of criteria were not addressed in (Partridge *et al*. 2024) study versus 55% not addressed in this study. Furthermore, much of the previous literature combine factors related to digital technology within the social determinants of health (Chidambaram *et al*. 2024). However, intrinsic technology design, use, and incorporation within healthcare and community settings have potential negative effects that extend beyond the social determinants of health (Chidambaram *et al*. 2024). Though social and DDoH are closely intertwined, DDoH should be considered as a separate construct which influences health equity.

Previous research demonstrates that the DDoH can be closely intertwined with adolescent attitudes towards digital health promotion (Raeside 2025). Therefore, it is vital that adolescents from the target population are engaged in the process of developing the intervention, ensuring that it meets the needs of that specific population. This study found that only 32% of studies addressed technology personalization, with an additional 21% of studies having somewhat addressed this criterion. Incorporating personalization within interventions has shown to improve user satisfaction, facilitate behaviour change, and enhance the overall efficacy of the interventions (Jahedi *et al*. 2024). Furthermore, adolescents identified a lack of personalization as a deterrent to engage with digital health promotion tools (Ferretti *et al*. 2023), leaving them at risk of being ineffective. Personalized interventions tailored to population-specific needs have the potential to drive greater behaviour change and improve nutrition-related outcomes across diverse groups of adolescents. Addressing this gap in future research through co-design and the integration of user preferences and experiences is essential for promoting digital nutrition interventions which advance equity.

An interesting finding from this study is that none of the included digital nutrition interventions used algorithms or AI to deliver the intervention. The use of AI and algorithmic technology is still within its infancy in healthcare. Therefore, though this criteria was not applicable for any of the included studies, it holds a valuable place within the DDoH assessment criteria and should be included in the future. Advances in this technology herald it as a promising avenue for delivering digital behaviour change interventions among adolescents (Rowe and Lester 2020). Giovanelli discussed how advancements in algorithmic bias and AI can enhance behaviour change in adolescents, offering new opportunities for engagement while reducing costs and saving time on human resources (Giovanelli *et al*. 2023). Future research should investigate how algorithmic bias can be harnessed to improve adolescent nutrition outcomes through expanding access to digital interventions and using AI to further personalize them. The personalization of interventions may help with existing issues of engaging adolescents within digital health interventions (Partridge and Redfern 2018, Whitehead *et al*. 2024), which may in turn increase adoption of the intervention. It may also be able to assist with equity by providing interventions which are more personalized, at the right digital literacy levels, enhancing usability and perceived usefulness for different population groups. Whether advancement of digital health technologies through using algorithms and AI to enhance digital health equity is still unknown.

### Strengths and limitations

To our knowledge, this is the first development of DDoH assessment criteria, establishing a clear framework to assess the extent to which DDoH are addressed in intervention design and delivery. By defining the DDoH dimensions and providing examples, the assessment criteria enable an objective evaluation of intervention potential to advance equity across studies. Though assessing studies through the criteria is a subjective process, we aimed to minimize bias using two reviewers to cross-check assessments made. Future research is recommended to apply the assessment criteria across different populations and intervention types to expand definitions and ensure development of a tool which is robust and ultimately increase capability to advance health equity. In addition, it is recommended that researchers report on the dimensions of DDoH when reporting all digital health interventions. During the course of our review, other DDoH have been identified (van Kessel *et al*. 2025). We acknowledge that this is a rapidly expanding field of research and definitions and criteria will change and require updating over time. Rather, we look at this as an essential starting point for developing criteria to assess the DDoH, particularly among interventions which target the individual level.

In this study, we used a comprehensive and systematic search strategy and followed PRISMA guidelines, capturing all available evidence and using best practice methods. However, several limitations were identified. Most studies (94%) were conducted in high and upper-middle-income countries, with only one study in a lower-middle-income country. This limits the generalisability of findings to all global contexts. A recent review demonstrates that low- and middle-income countries require greater support through digital determinants including digital literacy and digital accessibility (connectivity) for increased use of digital technologies within healthcare (Biga *et al*. 2024). Interventions were broad in nutrition behaviours or intake which were targeted, therefore meta-analysis was not possible. In addition, due to variations in the data we were unable to address the second aim of this review. However, a narrative synthesis of positive nutrition outcomes was outlined within the results. Nine of the studies (47%) had an overall high risk of bias, many due to inadequate reporting of trial analysis and outcomes. These limitations underscore the need for more robust research to advance equitable digital nutrition interventions for adolescents.

## CONCLUSION

This systematic review highlights a significant gap in addressing the DDoH in the development and delivery of digital nutrition interventions for adolescents. Establishing objective assessment criteria to assess the extent to which interventions incorporate the DDoH has potential to improve intervention design and delivery. However, due to the limited integration of the DDoH in existing interventions and insufficient reporting, this review was unable to determine whether addressing the DDoH influences adolescents access or use of digital nutrition interventions to improve nutrition outcomes. Future research should explore the direct implications of incorporating the DDoH into intervention design to enhance digital equity, especially among other populations and types of digital health interventions. Developing a structured approach to integrating the DDoH has potential to enhance the effectiveness and accessibility of digital nutrition interventions for adolescents, ultimately contributing to greater digital equity.

## Supplementary Material

daaf154_Supplementary_Data

## Data Availability

The data underlying this article are available in the article and in its online Supplementary material.
