# Supplementary material for: The inclusion and impact of digital determinants of health in digital nutrition interventions for adolescents: a systematic review
Source: Health Promot Int. 2025 Sep 29;40(5):daaf154. doi: 10.1093/heapro/daaf154 (PMC12477533; doi:10.1093/heapro/daaf154)
Supplement: daaf154_Supplementary_Data [file daaf154_supplementary_data.docx]

**Table S1.** PRISMA 2020 checklist

| **Section and Topic** | **Item #** | **Checklist item** | **Location where item is reported (Pg.)** |
| --- | --- | --- | --- |
| **TITLE** | | |  |
| Title | 1 | Identify the report as a systematic review. | 1 |
| **ABSTRACT** | | |  |
| Abstract | 2 | See the PRISMA 2020 for Abstracts checklist. | 2 |
| **INTRODUCTION** | | |  |
| Rationale | 3 | Describe the rationale for the review in the context of existing knowledge. | 4-6 |
| Objectives | 4 | Provide an explicit statement of the objective(s) or question(s) the review addresses. | 6 |
| **METHODS** | | |  |
| Eligibility criteria | 5 | Specify the inclusion and exclusion criteria for the review and how studies were grouped for the syntheses. | 7 |
| Information sources | 6 | Specify all databases, registers, websites, organisations, reference lists and other sources searched or consulted to identify studies. Specify the date when each source was last searched or consulted. | 8 |
| Search strategy | 7 | Present the full search strategies for all databases, registers and websites, including any filters and limits used. | 11 |
| Selection process | 8 | Specify the methods used to decide whether a study met the inclusion criteria of the review, including how many reviewers screened each record and each report retrieved, whether they worked independently, and if applicable, details of automation tools used in the process. | 8 |
| Data collection process | 9 | Specify the methods used to collect data from reports, including how many reviewers collected data from each report, whether they worked independently, any processes for obtaining or confirming data from study investigators, and if applicable, details of automation tools used in the process. | 8-9 |
| Data items | 10a | List and define all outcomes for which data were sought. Specify whether all results that were compatible with each outcome domain in each study were sought (e.g. for all measures, time points, analyses), and if not, the methods used to decide which results to collect. | 8-10 |
|  | 10b | List and define all other variables for which data were sought (e.g. participant and intervention characteristics, funding sources). Describe any assumptions made about any missing or unclear information. | 8-10 |
| Study risk of bias assessment | 11 | Specify the methods used to assess risk of bias in the included studies, including details of the tool(s) used, how many reviewers assessed each study and whether they worked independently, and if applicable, details of automation tools used in the process. | 10 |
| Effect measures | 12 | Specify for each outcome the effect measure(s) (e.g. risk ratio, mean difference) used in the synthesis or presentation of results. | N/A |
| Synthesis methods | 13a | Describe the processes used to decide which studies were eligible for each synthesis (e.g. tabulating the study intervention characteristics and comparing against the planned groups for each synthesis (item #5)). | 9-10 |
|  | 13b | Describe any methods required to prepare the data for presentation or synthesis, such as handling of missing summary statistics, or data conversions. | N/A |
|  | 13c | Describe any methods used to tabulate or visually display results of individual studies and syntheses. | 8-10 |
|  | 13d | Describe any methods used to synthesize results and provide a rationale for the choice(s). If meta-analysis was performed, describe the model(s), method(s) to identify the presence and extent of statistical heterogeneity, and software package(s) used. | 8-10 |
|  | 13e | Describe any methods used to explore possible causes of heterogeneity among study results (e.g. subgroup analysis, meta-regression). | N/A |
|  | 13f | Describe any sensitivity analyses conducted to assess robustness of the synthesized results. | N/A |
| Reporting bias assessment | 14 | Describe any methods used to assess risk of bias due to missing results in a synthesis (arising from reporting biases). | 10 |
| Certainty assessment | 15 | Describe any methods used to assess certainty (or confidence) in the body of evidence for an outcome. | N/A |
| **RESULTS** | | |  |
| Study selection | 16a | Describe the results of the search and selection process, from the number of records identified in the search to the number of studies included in the review, ideally using a flow diagram. | 10 |
|  | 16b | Cite studies that might appear to meet the inclusion criteria, but which were excluded, and explain why they were excluded. | Supp. Table S8 |
| Study characteristics | 17 | Cite each included study and present its characteristics. | 10-15 |
| Risk of bias in studies | 18 | Present assessments of risk of bias for each included study. | Supp. Table S9 |
| Results of individual studies | 19 | For all outcomes, present, for each study: (a) summary statistics for each group (where appropriate) and (b) an effect estimate and its precision (e.g. confidence/credible interval), ideally using structured tables or plots. | 10-15, Table 4, Table 5 |
| Results of syntheses | 20a | For each synthesis, briefly summarise the characteristics and risk of bias among contributing studies. | 15-16, Supp. Table S9 |
|  | 20b | Present results of all statistical syntheses conducted. If meta-analysis was done, present for each the summary estimate and its precision (e.g. confidence/credible interval) and measures of statistical heterogeneity. If comparing groups, describe the direction of the effect. | N/A |
|  | 20c | Present results of all investigations of possible causes of heterogeneity among study results. | N/A |
|  | 20d | Present results of all sensitivity analyses conducted to assess the robustness of the synthesized results. | N/A |
| Reporting biases | 21 | Present assessments of risk of bias due to missing results (arising from reporting biases) for each synthesis assessed. | 15-16, Supp. Table S9 |
| Certainty of evidence | 22 | Present assessments of certainty (or confidence) in the body of evidence for each outcome assessed. | N/A |
| **DISCUSSION** | | |  |
| Discussion | 23a | Provide a general interpretation of the results in the context of other evidence. | 16-18 |
|  | 23b | Discuss any limitations of the evidence included in the review. | 19 |
|  | 23c | Discuss any limitations of the review processes used. | 19 |
|  | 23d | Discuss implications of the results for practice, policy, and future research. | 20 |
| **OTHER INFORMATION** | | |  |
| Registration and protocol | 24a | Provide registration information for the review, including register name and registration number, or state that the review was not registered. | 7 |
|  | 24b | Indicate where the review protocol can be accessed, or state that a protocol was not prepared. | 7 |
|  | 24c | Describe and explain any amendments to information provided at registration or in the protocol. | 7 |
| Support | 25 | Describe sources of financial or non-financial support for the review, and the role of the funders or sponsors in the review. | 21 |
| Competing interests | 26 | Declare any competing interests of review authors. | 21 |
| Availability of data, code and other materials | 27 | Report which of the following are publicly available and where they can be found: template data collection forms; data extracted from included studies; data used for all analyses; analytic code; any other materials used in the review. | 21 |

**Table S2.** Search strategy for PreMEDLINE, MEDLINE (# results displayed), Embase, Cochrane, Cochrane Central Register of Controlled Trials (CENTRAL), and AMED (via OvidSP 1946- present)

| Search Strategy terms | # of results |
| --- | --- |
| 1 Adolescent/ or Adolesc*.mp. 2367967  2 Teen*.mp. 37760  3 Young adult*.mp. or Young Adult/ 1133891  4 Youth.mp. 103908  5 1 or 2 or 3 or 4 2931791  6 Telemedicine/ or digital interventions.mp. 42266  7 digital health interventions.mp. 936  8 telehealth.mp. 16712  9 digital health.mp. or Digital Health/ 10809  10 mobile health.mp. 13987  11 ehealth.mp. 8073  12 Electronic Health Records/ or e-health.mp. 33573  13 mhealth.mp. 10935  14 m-health.mp. 1021  15 Cell Phone/ or Cellphone*.mp. 10871  16 cell phone*.mp. 12853  17 internet.mp. or Internet/ 138869  18 mobile application.mp. or Mobile Applications/ 15629  19 mobile*.mp. 151512  20 world wide web.mp. 3473  21 online*.mp. 257246  22 telemedicine.mp. 54466  23 text messag*.mp. or Text Messaging/ 9054  24 SMS.mp. 8902  25 Smartphone*.mp. or Smartphone/ 29159  26 social media.mp. or Social Media/ 41200  27 Electronic Mail/ or email.mp. 13747  28 e-mail.mp. 8504  29 Wearable Electronic Devices/ or Smartwatch*.mp. 10564  30 Wearable device*.mp. 8019  31 activity tracker*.mp. or Fitness Trackers/ 2022  32 Fitness tracker*.mp. 1588  33 personal digital assistant.mp. 602  34 artificial intelligence.mp. or Artificial Intelligence/ 79731  35 AI.mp. 62018  36 6 or 7 or 9 or 10 or 11 or 12 or 13 or 14 or 15 or 16 or 17 or 18 or 19 or 20 or 21 or 22 or 23 or 24 or 25 or 26 or 27 or 29 or 30 or 31 or 32 or 33 or 34 or 35 756803  37 Diet/ or diet*.mp. 935173  38 nutrition*.mp. 502522  39 "Diet, Food, and Nutrition"/ or food*.mp. or Food/ 836685  40 Obesity/ or obesity.mp. 431720  41 malnutrition.mp. or Malnutrition/ or Protein-Energy Malnutrition/ 65449  42 undernutrition.mp. 10083  43 overnutrition.mp. or Overnutrition/ 2480  44 fruit intake.mp. 1607  45 vegetable intake.mp. 4742  46 processed food.mp. or Food, Processed/ 2758  47 Sugar sweetened beverages.mp. or Sugar-Sweetened Beverages/ 4121  48 Adolescent nutrition.mp. 219  49 Nutritional status.mp. or Nutritional Status/ 82199  50 food intake.mp. or Eating/ 99110  51 Food frequency questionnaire.mp. 14755  52 Diet Surveys/ or Diet Records/ or FFQ.mp. 18716  53 Dietary guidelines.mp. 4775  54 Diet record*.mp. 6923  55 AGHE.mp. 44  56 Australian guide to healthy eating.mp. 77  57 food behavio*.mp. 685  58 Fast food.mp. or Fast Foods/ 6525  59 Nutritional Requirements/ or Nutrition* requirement.mp. 20561  60 Dietary Sugars/ or Sugar.mp. or Sugars/ 130904  61 37 or 38 or 39 or 40 or 41 or 42 or 43 or 44 or 45 or 46 or 47 or 48 or 49 or 50 or 51 or 52 or 53 or 54 or 55 or 56 or 57 or 58 or 59 or 60 2247418  62 5 and 36 and 61 7682  63 Randomized Controlled Trials as Topic/ or control* trial*.mp. 953297  64 Clinical Trials as Topic/ or clinical trial*.mp. 1222364  65 63 or 64 1767884  66 62 and 65 1251  67 limit 66 to yr="2005 -Current" 1228 | 1228 |

**Table S3.** Search strategy for CINAHL

| # | Query | no. of results |
| --- | --- | --- |
| S71 | S5 AND S39 AND S66 AND S69 | 736 |
| S70 | S5 AND S39 AND S66 AND S69 |  |
| S69 | S67 OR S68 |  |
| S68 | (MH "Clinical Trials+") OR "clinical trial" |  |
| S67 | (MH "Randomized Controlled Trials+") OR "control* trial*" |  |
| S66 | S40 OR S41 OR S42 OR S43 OR S44 OR S45 OR S46 OR S47 OR S48 OR S49 OR S50 OR S51 OR S52 OR S53 OR S54 OR S55 OR S56 OR S57 OR S58 OR S59 OR S60 OR S61 OR S62 OR S63 OR S64 OR S65 |  |
| S65 | “FFQ” |  |
| S64 | "Sugar" |  |
| S63 | (MH "Nutritional Requirements+") OR "Nutrition* requirement" |  |
| S62 | (MH "Fast Foods") OR "Fast food" |  |
| S61 | "food behavio*" |  |
| S60 | "Australian guide to healthy eating" |  |
| S59 | "AGHE" |  |
| S58 | "Dietary guidelines" |  |
| S57 | "Diet Surveys" |  |
| S56 | (MH "Diet Records") OR "Diet records" |  |
| S55 | "food frequency questionnaire" |  |
| S54 | (MH "Eating") OR "eating" |  |
| S53 | (MH "Food Intake+") OR "food intake" |  |
| S52 | (MH "Nutritional Status") OR "Nutritional status") |  |
| S51 | (MH "Adolescent Nutrition") OR "Adolescent nutrition" |  |
| S50 | (MH "Sweetened Beverages") OR "sugar sweetened beverages" |  |
| S49 | "processed food" OR (MH "Food, Commercially Packaged") |  |
| S48 | "vegetable intake" |  |
| S47 | "fruit intake" |  |
| S46 | "overnutrition" |  |
| S45 | (MH "Undernutrition") OR "undernutrition" |  |
| S44 | (MH "Malnutrition+") OR "malnutrition" |  |
| S43 | (MH "Obesity+") OR "obesity" |  |
| S42 | (MH "Food+") OR "food*" |  |
| S41 | (MH "Nutrition+") OR "nutrition*" |  |
| S40 | (MH "Diet+") OR "diet*" |  |
| S39 | S6 OR S7 OR S8 OR S9 OR S10 OR S11 OR S12 OR S13 OR S14 OR S15 OR S16 OR S17 OR S18 OR S19 OR S20 OR S21 OR S22 OR S23 OR S24 OR S25 OR S26 OR S27 OR S28 OR S29 OR S30 OR S31 OR S32 OR S33 OR S34 OR S35 OR S36 OR S37 OR S38 |  |
| S38 | "Electronic mail" |  |
| S37 | "AI" |  |
| S36 | (MH "Artificial Intelligence+") OR "artificial intelligence" |  |
| S35 | "personal digital assistant" |  |
| S34 | "Fitness tracker*" |  |
| S33 | (MH "Fitness Trackers") OR "activity tracker*" |  |
| S32 | "wearable device*" |  |
| S31 | "wearable electronic device*" |  |
| S30 | "smartwatch*" |  |
| S29 | "e-mail" |  |
| S28 | (MH "Email") OR "email" |  |
| S27 | (MH "Social Media+") OR "social media" |  |
| S26 | (MH "Smartphone") OR "Smartphone*" |  |
| S25 | "SMS" |  |
| S24 | (MH "Text Messaging+") OR "text messag*" |  |
| S23 | "telemedicine" |  |
| S22 | "online*" |  |
| S21 | (MH "World Wide Web+") OR "world wide web" |  |
| S20 | "mobile*" |  |
| S19 | (MH "Mobile Applications") OR "mobile application*" |  |
| S18 | (MH "Internet+") OR "internet" |  |
| S17 | "cellphone*" |  |
| S16 | (MH "Electronic Health Records+") OR "electronic health records" |  |
| S15 | "m-health" |  |
| S14 | "mhealth" |  |
| S13 | (MH "Electronic Health Records+") OR "electronic health records" |  |
| S12 | "e-health" |  |
| S11 | "ehealth" |  |
| S10 | "mobile health" OR (MH "Telehealth+") |  |
| S9 | (MH "Digital Health+") OR "digital health" |  |
| S8 | "digital health intervention" |  |
| S7 | (MH "Telemedicine+") OR "telemedicine" |  |
| S6 | "digital intervention" |  |
| S5 | S1 OR S2 OR S3 OR S4 |  |
| S4 | "Youth" |  |
| S3 | (MH "Young Adult") OR "Young adult*" |  |
| S2 | "Teen*" |  |
| S1 | (MH "Adolescence+") OR "Adolesce*" |  |

**Table S4.** Search strategy for Scopus

| ( TITLE-ABS-KEY ( adolescent OR adolesc* OR teen OR "Young adult" OR youth ) AND TITLE-ABS-KEY ( telemedicine OR "digital interventions" OR "digital health interventions" OR "digital health" OR "Digital Health" OR "mobile health" OR ehealth OR "Electronic Health Records" OR e-health OR mhealth OR m-health OR "Cell Phone" OR cellphone OR cellphone* OR internet OR internet OR "mobile application" OR "Mobile Applications" OR mobile* OR online* OR telemedicine OR "text messaging" OR "Text Messaging" OR sms OR smartphone* OR smartphone OR "social AND media" OR "electronic AND mail" OR email OR e-mail OR "wearable AND electronic AND devices" OR smartwatch* OR "wearable AND device*" OR "activity AND tracker*" OR "Fitness AND trackers" OR "fitness AND tracker*" OR "personal AND digital AND assistant" OR "artificial AND intelligence" OR ai ) AND TITLE-ABS-KEY ( diet OR diet* OR nutrition* OR "diet, AND food, AND nutrition" OR food* OR food OR obesity OR malnutrition OR "protein-energy AND malnutrition" OR undernutrition OR overnutrition OR "fruit AND intake" OR "vegetable AND intake" OR "processed AND food" OR "food, AND processed" OR "sugar AND sweetened AND beverages" OR "sugar-sweetened AND beverages" OR "adolescent AND nutrition" OR "nutritional AND status" OR "food AND intake" OR eating OR "food AND frequency AND questionnaire" OR "diet AND surveys" OR "diet AND records" OR ffq OR "dietary AND guidelines" OR "diet AND record*" OR aghe OR "australian AND guide AND to AND healthy AND eating" OR "food AND behaviours" OR "fast AND food*" OR "nutritional AND requirements" OR "nutrition* AND requirements" OR "dietary AND sugars" OR sugars OR sugar* ) AND TITLE-ABS-KEY ( "control* AND trial*" OR "clinical trial*" ) ) AND PUBYEAR > 2004 AND PUBYEAR < 2025 |
| --- |

**Table S5.** Search strategy for Web of Science

| (((((((TS=(Adolescen*)) OR TS=(Teen*)) OR TS=(Young adult*)) OR TS=(Youth)) OR TI=(Adolescen*)) OR TI=(Teen*)) OR TI=(Young adult*)) OR TI=(Youth)  (((((((((((((((((((((((((((((((((((((((((((((((((((((((((((((((((TS=(digital interventions)) OR TS=(Telemedicine)) OR TS=(digital health interventions)) OR TS=(digital health)) OR TS=(mobile health)) OR TS=(ehealth)) OR TS=(e-health)) OR TS=(Electronic Health Records)) OR TS=(mhealth)) OR TS=(m-health)) OR TS=(cellphone*)) OR TS=(Cell phone*)) OR TS=(internet)) OR TS=(Mobile Application*)) OR TS=(mobile*)) OR TS=(world wide web)) OR TS=(online*)) OR TS=(text messag*)) OR TS=(SMS)) OR TS=(Smartphone*)) OR TS=(social media)) OR TS=(email)) OR TS=(Electronic Mail)) OR TS=(e-mail)) OR TS=(Wearable Electronic Devices)) OR TS=(Smartwatch*)) OR TS=(Wearable device*)) OR TS=(activity tracker*)) OR TS=(Fitness Tracker*)) OR TS=(personal digital assistant )) OR TS=(artificial intelligence )) OR TS=(AI)) OR TI=(digital interventions)) OR TI=(Telemedicine)) OR TI=(digital health interventions)) OR TI=(digital health)) OR TI=(mobile health)) OR TI=(ehealth)) OR TI=(e-health)) OR TI=(Electronic Health Records)) OR TI=(mhealth)) OR TI=(m-health)) OR TI=(cellphone*)) OR TI=(Cell phone*)) OR TI=(internet)) OR TI=(Mobile Application*)) OR TI=(mobile*)) OR TI=(world wide web)) OR TI=(online*)) OR TI=(text messag*)) OR TI=(SMS)) OR TI=(Smartphone*)) OR TI=(social media)) OR TI=(email)) OR TI=(Electronic Mail)) OR TI=(e-mail)) OR TI=(Wearable Electronic Devices)) OR TI=(Smartwatch*)) OR TI=(Wearable device*)) OR TI=(activity tracker*)) OR TI=(Fitness Tracker*)) OR TI=(personal digital assistant)) OR TI=(artificial intelligence)) OR TI=(AI)) OR TS=(telehealth)) OR TI=(telehealth)  (((((((((((((((((((((((((((((((((((((((((((((((((((((TS=(diet*)) OR TS=(nutrition*)) OR TS=(food*)) OR TS=(obesity )) OR TS=(malnutrition)) OR TS=(undernutrition)) OR TS=(overnutrition)) OR TS=(fruit intake)) OR TS=(vegetable intake )) OR TS=(processed food )) OR TS=(Sugar sweetened beverages )) OR TS=(Adolescent nutrition)) OR TS=(Nutritional status)) OR TS=(food intake )) OR TS=(eating )) OR TS=(Food frequency questionnaire)) OR TS=(Diet Surveys)) OR TS=(Diet Record*)) OR TS=(FFQ)) OR TS=(Dietary guidelines)) OR TS=(AGHE)) OR TS=(Australian guide to healthy eating)) OR TS=(food behavio*)) OR TS=(Fast food*)) OR TS=(Nutrition* requirement)) OR TS=(Sugar)) OR TI=(diet*)) OR TI=(nutrition*)) OR TI=(food*)) OR TI=(obesity)) OR TI=(malnutrition)) OR TI=(undernutrition)) OR TI=(overnutrition)) OR TI=(fruit intake)) OR TI=(vegetable intake)) OR TI=(processed food)) OR TI=(Sugar sweetened beverages)) OR TI=(Adolescent nutrition)) OR TI=(Nutritional status)) OR TI=(food intake)) OR TI=(eating)) OR TI=(Food frequency questionnaire)) OR TI=(Diet Surveys)) OR TI=(Diet Record*)) OR TI=(FFQ)) OR TI=(Dietary guidelines)) OR TI=(AGHE)) OR TI=(Australian guide to healthy eating)) OR TI=(food behavio*)) OR TI=(Fast food*)) OR TI=(Nutrition* requirement)) OR TI=(Sugar))  (TS=(control* trial*)) OR TS=(clinical trial*)  #4 AND #3 AND #2 AND #1  Search: #5 and 2024 or 2023 or 2022 or 2021 or 2020 or 2019 or 2018 or 2017 or 2016 or 2015 or 2014 or 2013 or 2012 or 2011 or 2010 or 2009 or 2008 or 2007 or 2006 or 2005 (Publication Years) |
| --- |

**Table S6.** Search strategy for Informit

| [All Fields:adolescent OR All Fields:adolesc* OR All Fields:teen OR All Fields:"Young adult" OR All Fields:youth] AND [All Fields:telemedicine OR All Fields:"digital interventions" OR All Fields:"digital health interventions" OR All Fields:"digital health" OR All Fields:"Digital Health" OR All Fields:"mobile health" OR All Fields:ehealth OR All Fields:"Electronic Health Records" OR All Fields:e-health OR All Fields:mhealth OR All Fields:m-health OR All Fields:"Cell Phone" OR All Fields:cellphone OR All Fields:cellphone* OR All Fields:internet OR All Fields:internet OR All Fields:"mobile application" OR All Fields:"Mobile Applications" OR All Fields:mobile* OR All Fields:online* OR All Fields:telemedicine OR All Fields:"text messaging" OR All Fields:"Text Messaging" OR All Fields:sms OR All Fields:smartphone* OR All Fields:smartphone OR All Fields:"social OR All Fields:media" OR All Fields:"electronic OR All Fields:mail" OR All Fields:email OR All Fields:e-mail OR All Fields:"wearable OR All Fields:electronic OR All Fields:devices" OR All  Fields:smartwatch* OR All Fields:"wearable OR All Fields:device*" OR All Fields:"activity] AND [All Fields:tracker*" OR All Fields:"Fitness OR All Fields:trackers" OR All Fields:"fitness OR All Fields:tracker*" OR All Fields:"personal OR All Fields:digital OR All Fields:assistant" OR All Fields:"artificial OR All Fields:intelligence" OR All Fields:ai OR All Fields:diet OR All Fields:diet* OR All Fields:nutrition* OR All Fields:"diet, OR All Fields:food, OR All Fields:nutrition" OR All Fields:food* OR All Fields:food OR All Fields:obesity OR All Fields:malnutrition OR All Fields:"protein-energy OR All Fields:malnutrition" OR All Fields:undernutrition OR All Fields:overnutrition OR All Fields:"fruit OR All Fields:intake" OR All Fields:"vegetable OR All Fields:intake" OR All Fields:"processed OR All Fields:food" OR All Fields:"food, OR All Fields:processed" OR All Fields:"sugar OR All Fields:sweetened OR All Fields:beverages"] AND Publication Date: (01/01/2005 TO 31/12/2024) |
| --- |

**Table S7:** Database search date, number of results and corresponding screenshot figure

| **Databases** | **Search Date** | **# of records** |
| --- | --- | --- |
| Medline | 26 Aug 2024 | 1228 |
| PreMedline | 26 Aug 2024 | 5 |
| Cochrane | 26 Aug 2024 | 186 |
| Cochrane Central | 26 Aug 2024 | 1206 |
| Embase | 26 Aug 2024 | 1397 |
| AMED | 26 Aug 2024 | 5 |
| CINAHL | 26 Aug 2024 | 737 |
| Scopus | 26 Aug 2024 | 809 |
| Web of Science | 26 Aug 2024 | 1020 |
| Informit | 26 Aug 2024 | 452 |

**Figure S1.** MEDLINE Search Screenshot


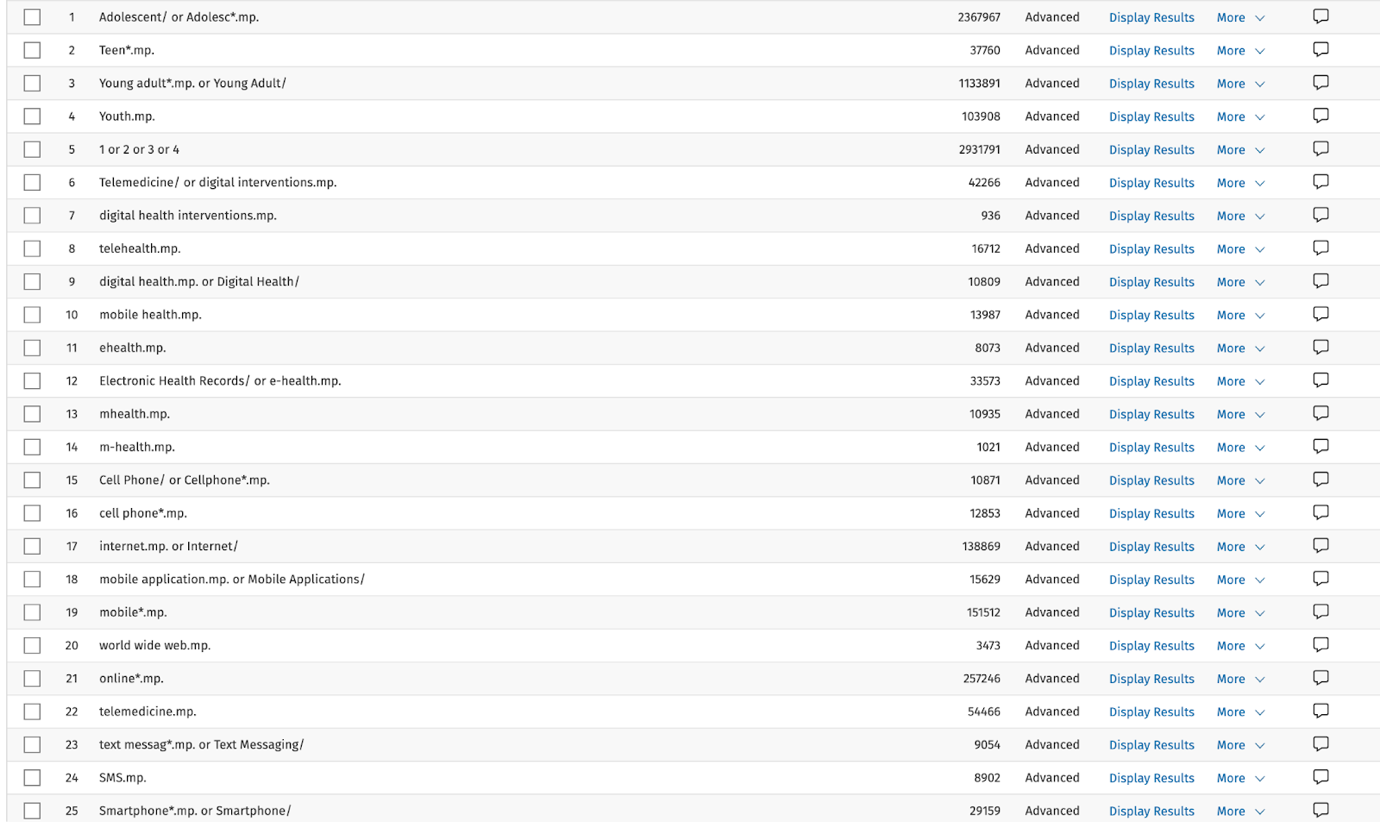


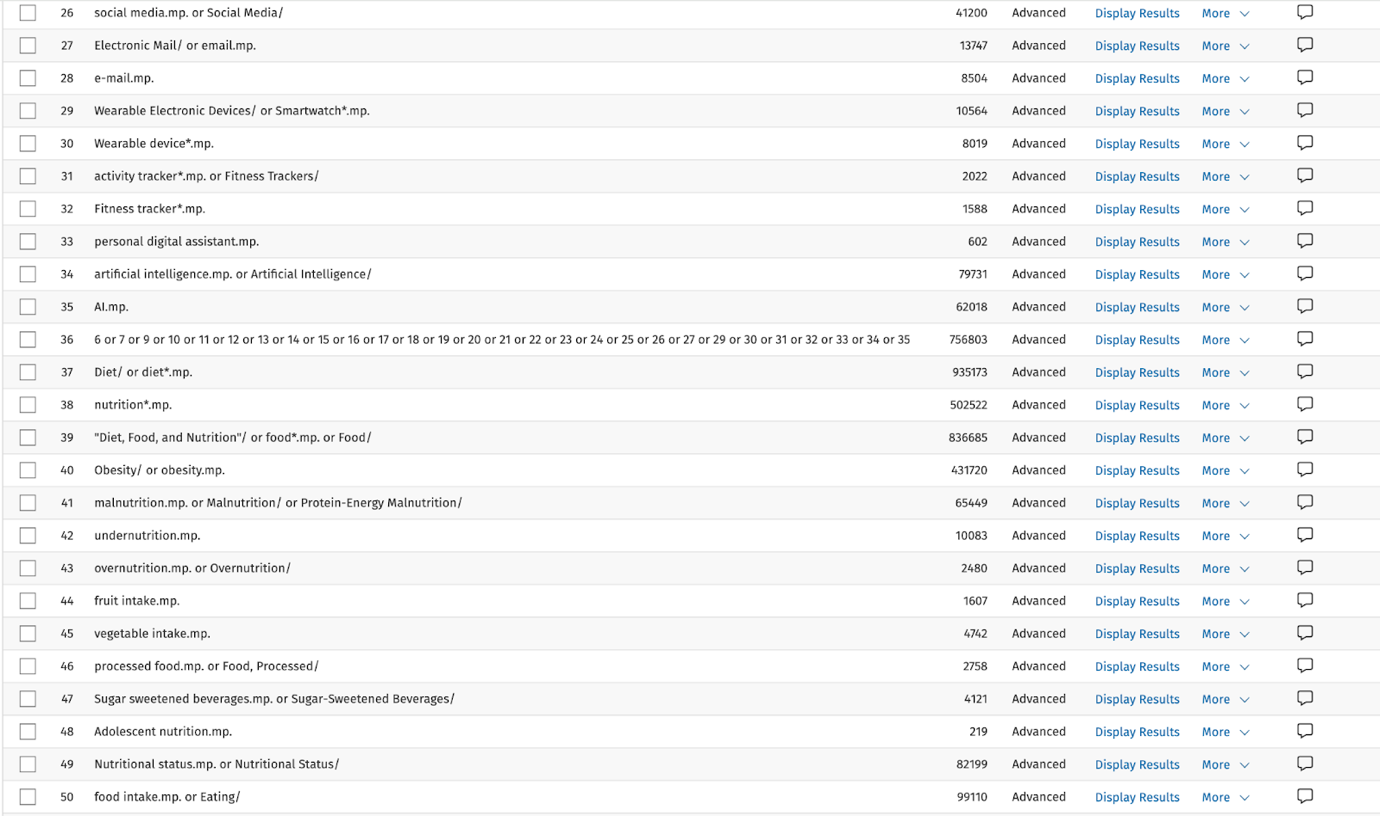


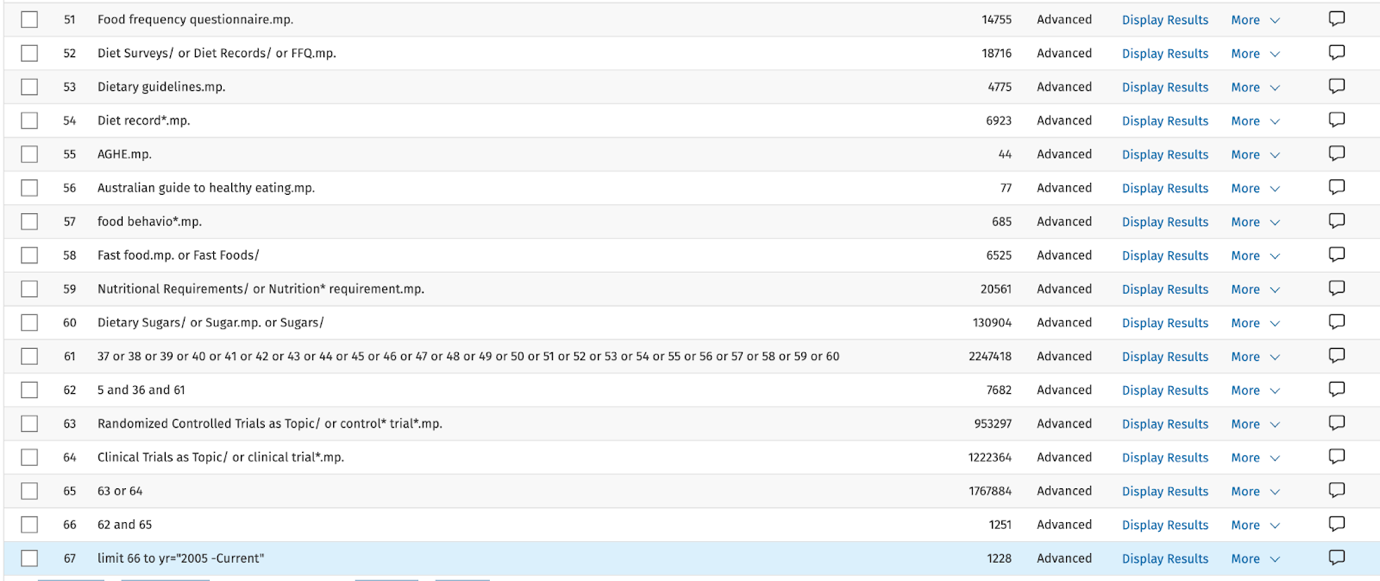


**Figure S2.** PreMEDLINE Search Screenshot


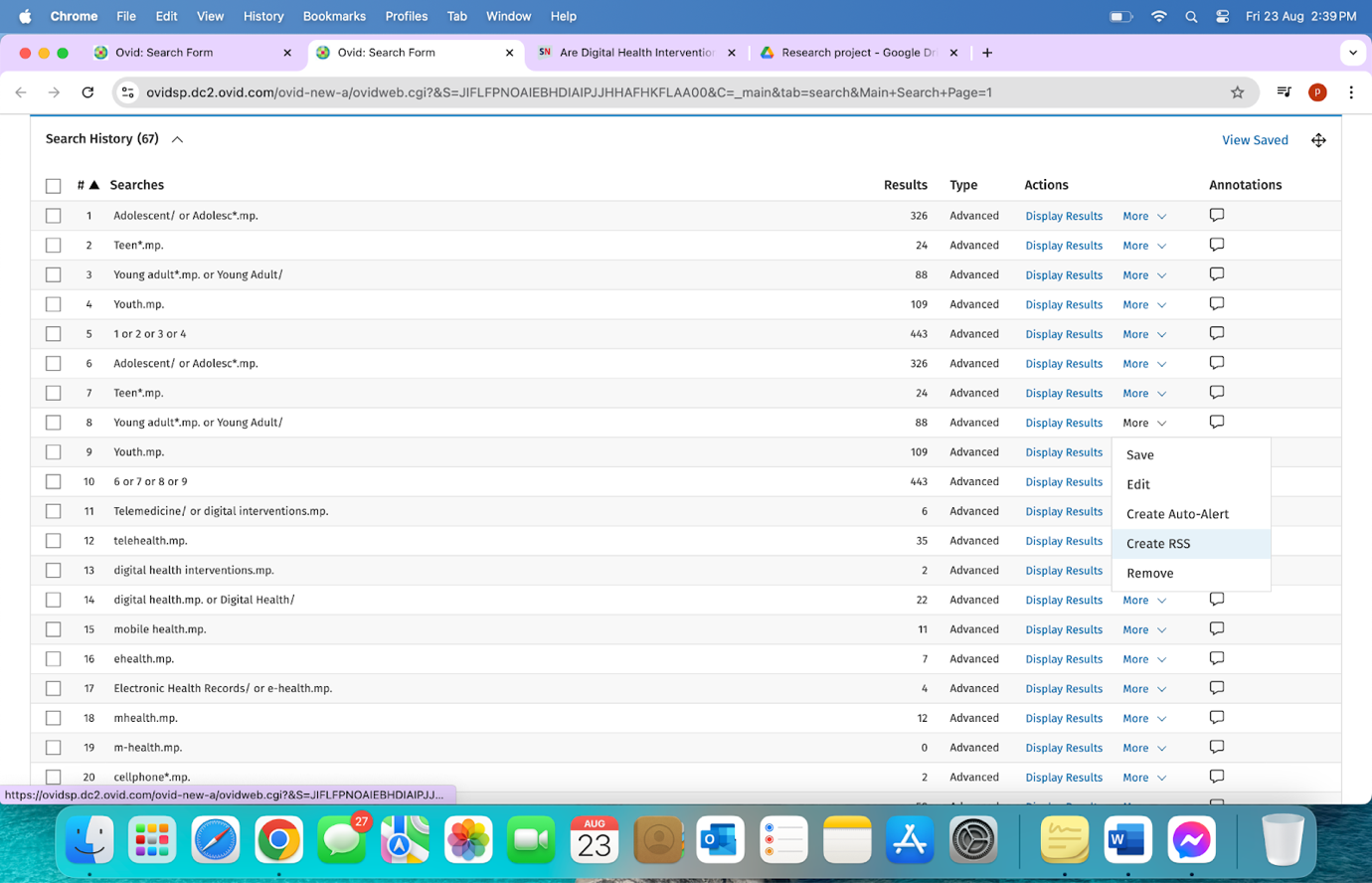


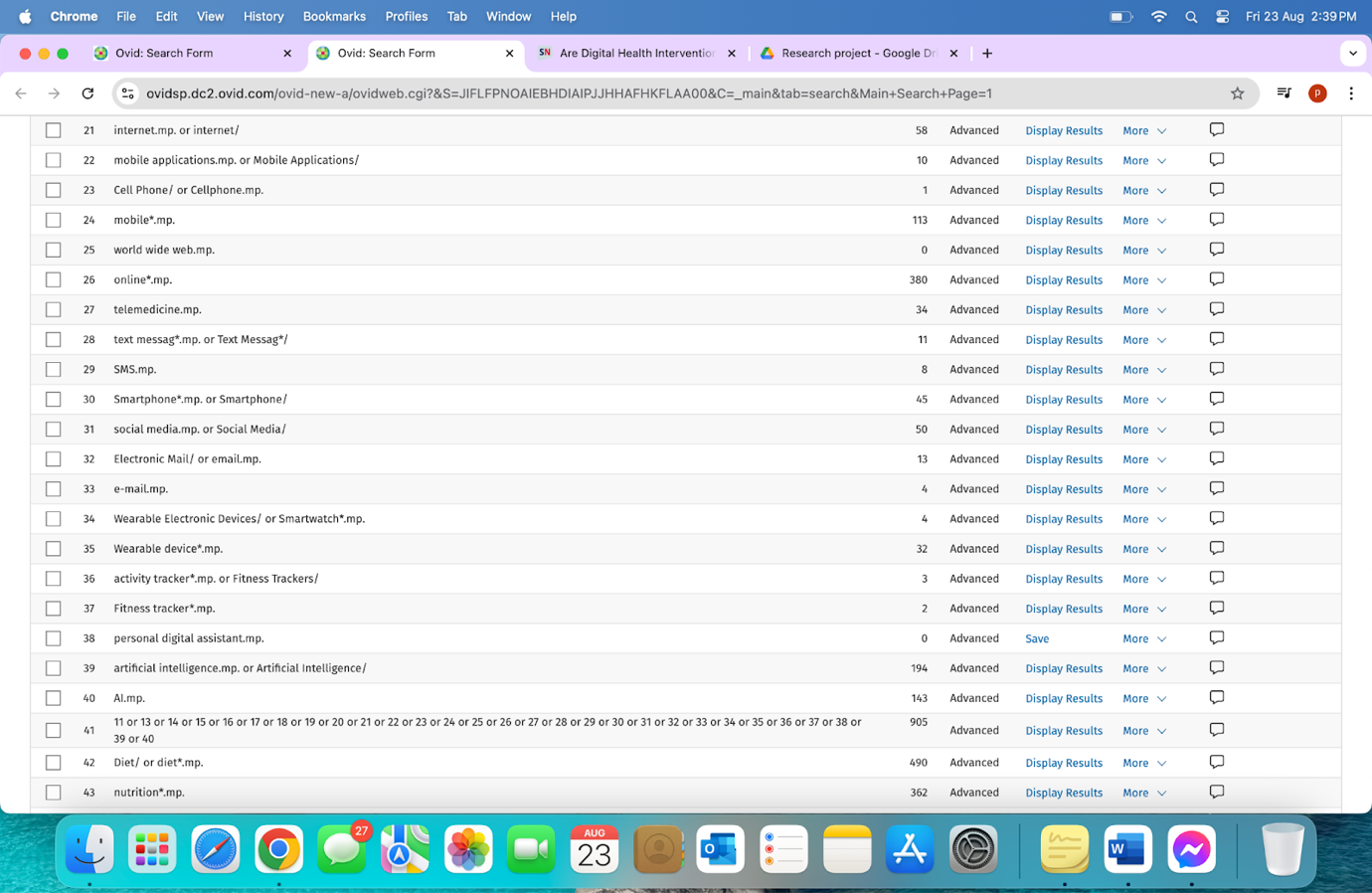


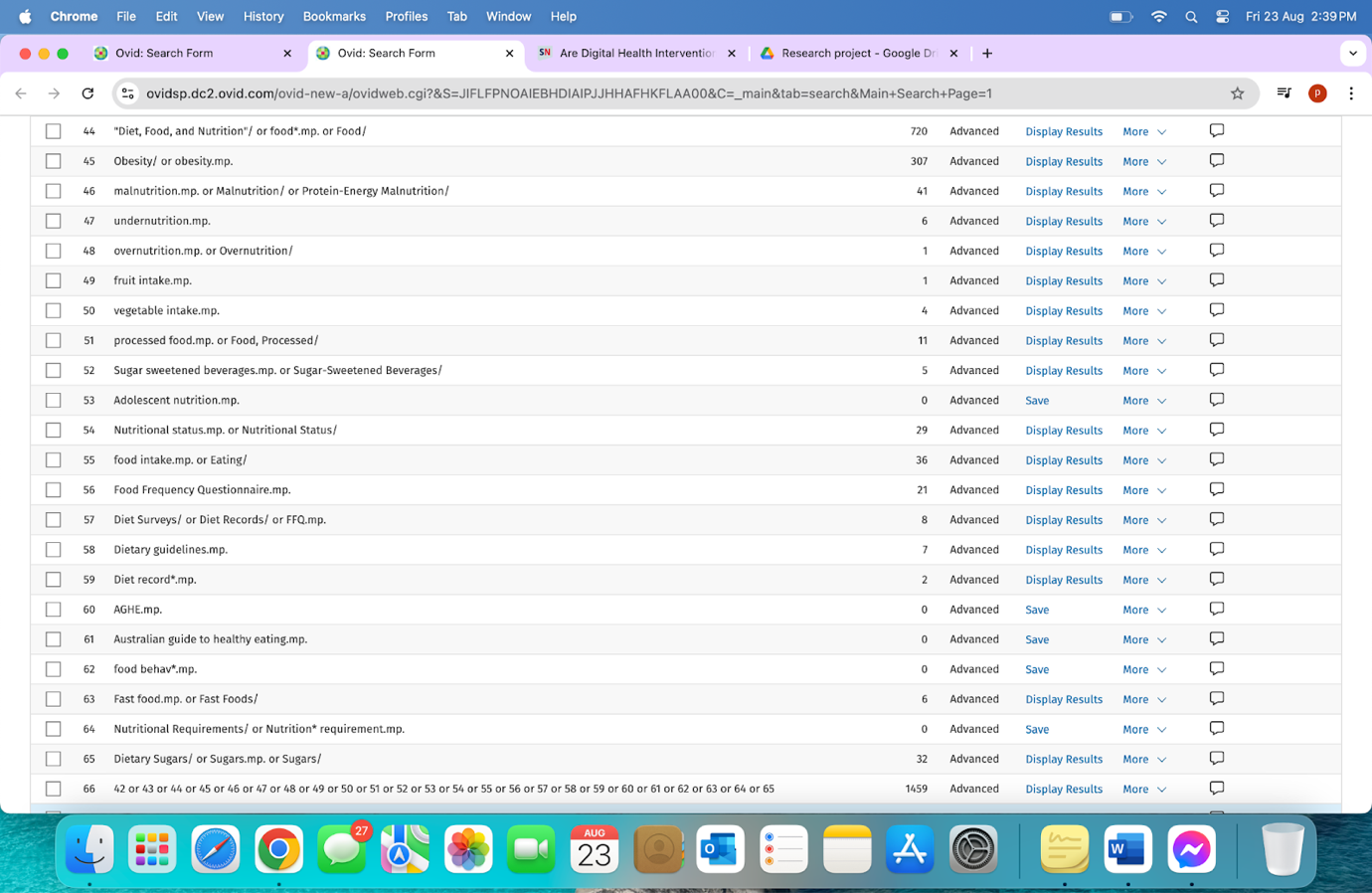


**Figure S3**. Cochrane Search Screenshot


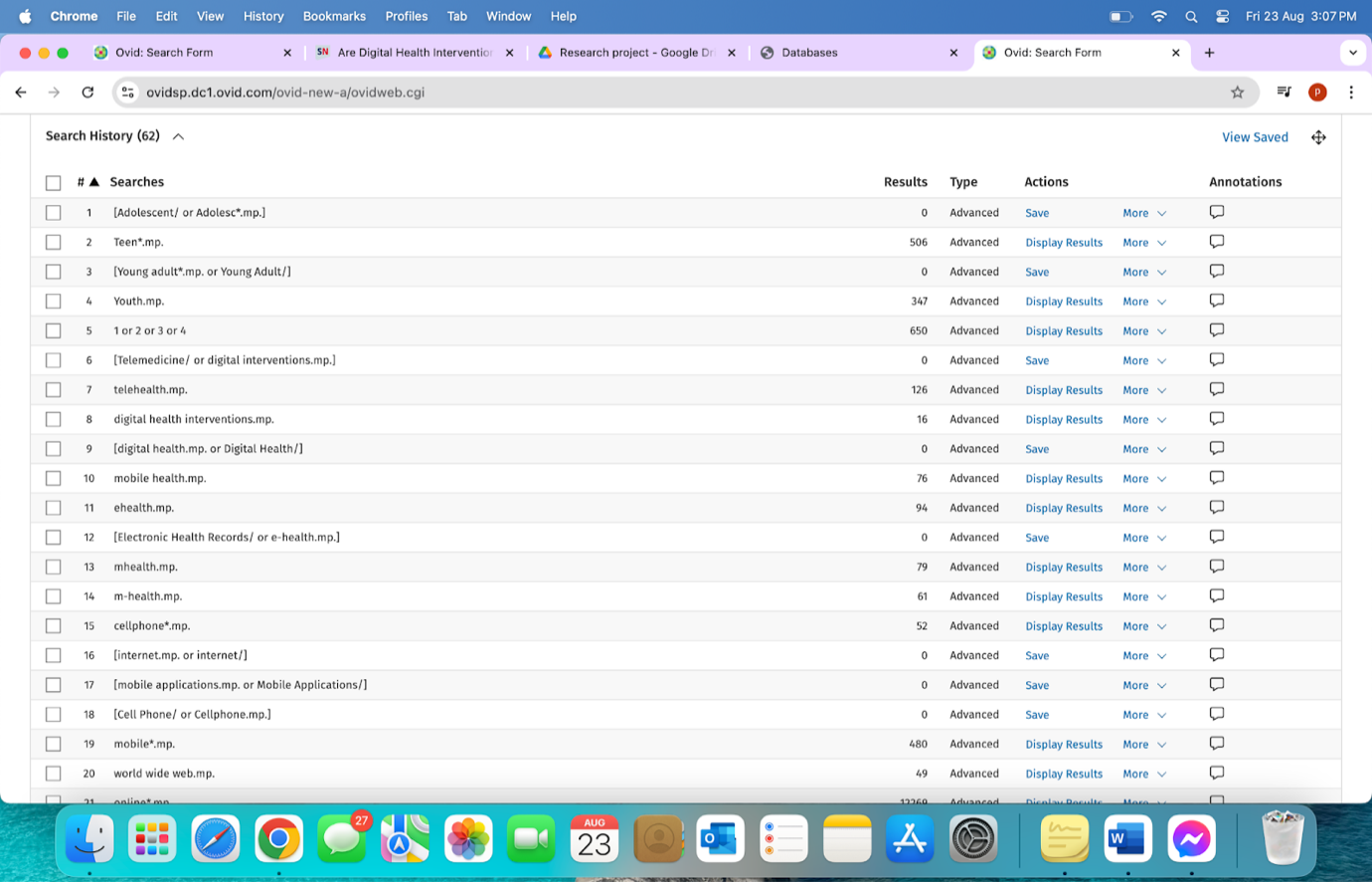


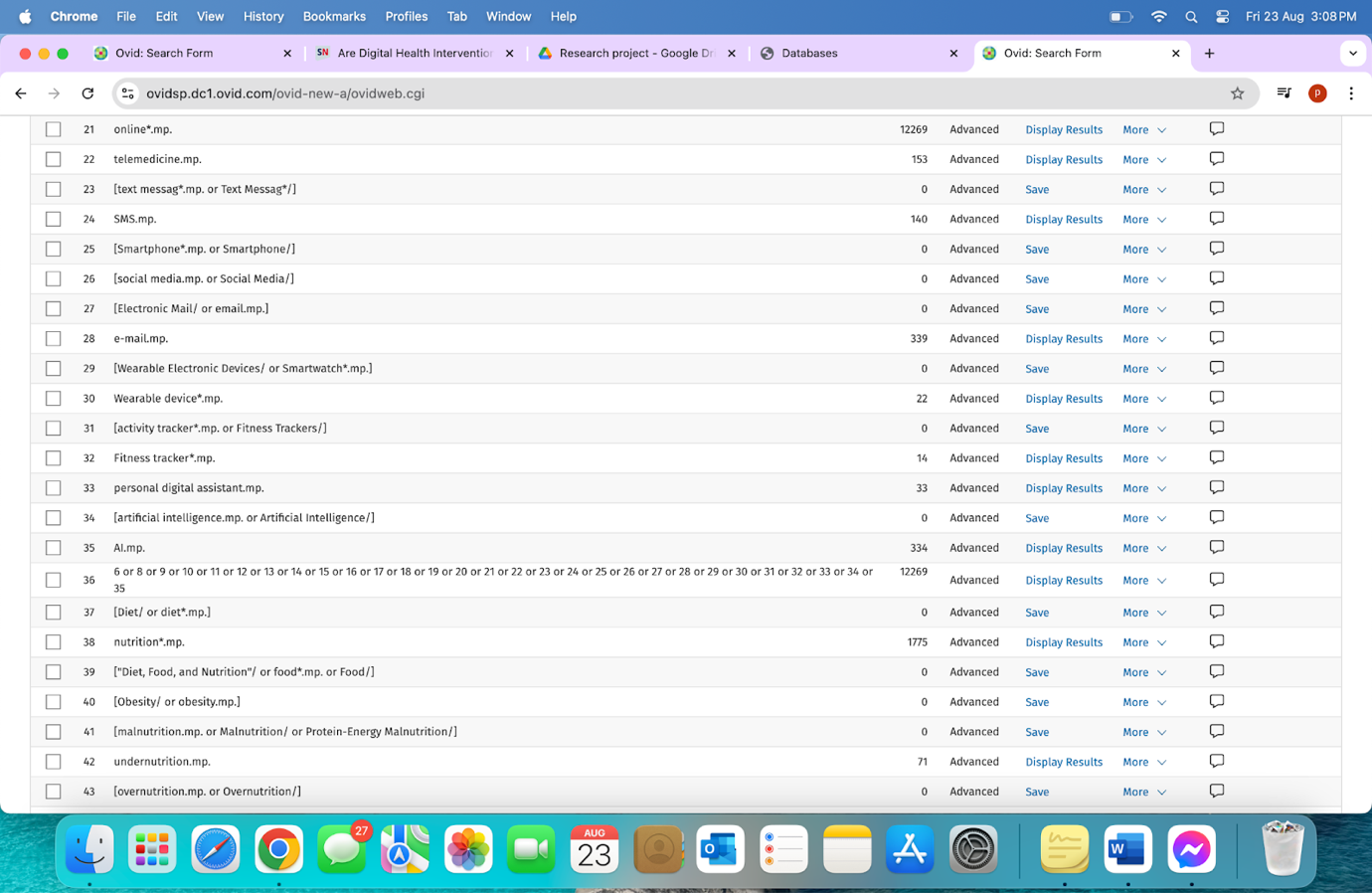


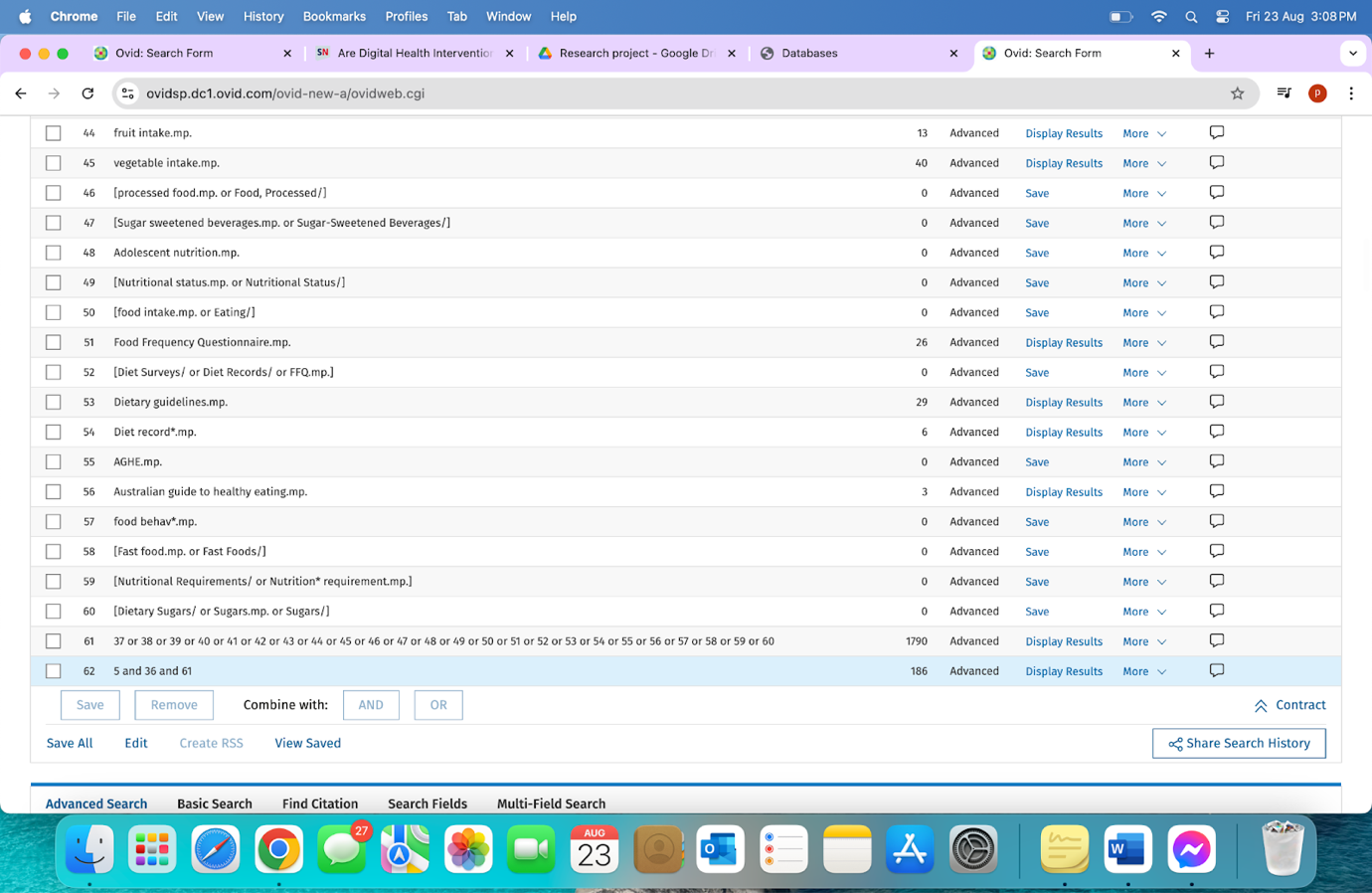


**Figure S4.** CENTRAL Search Screenshot


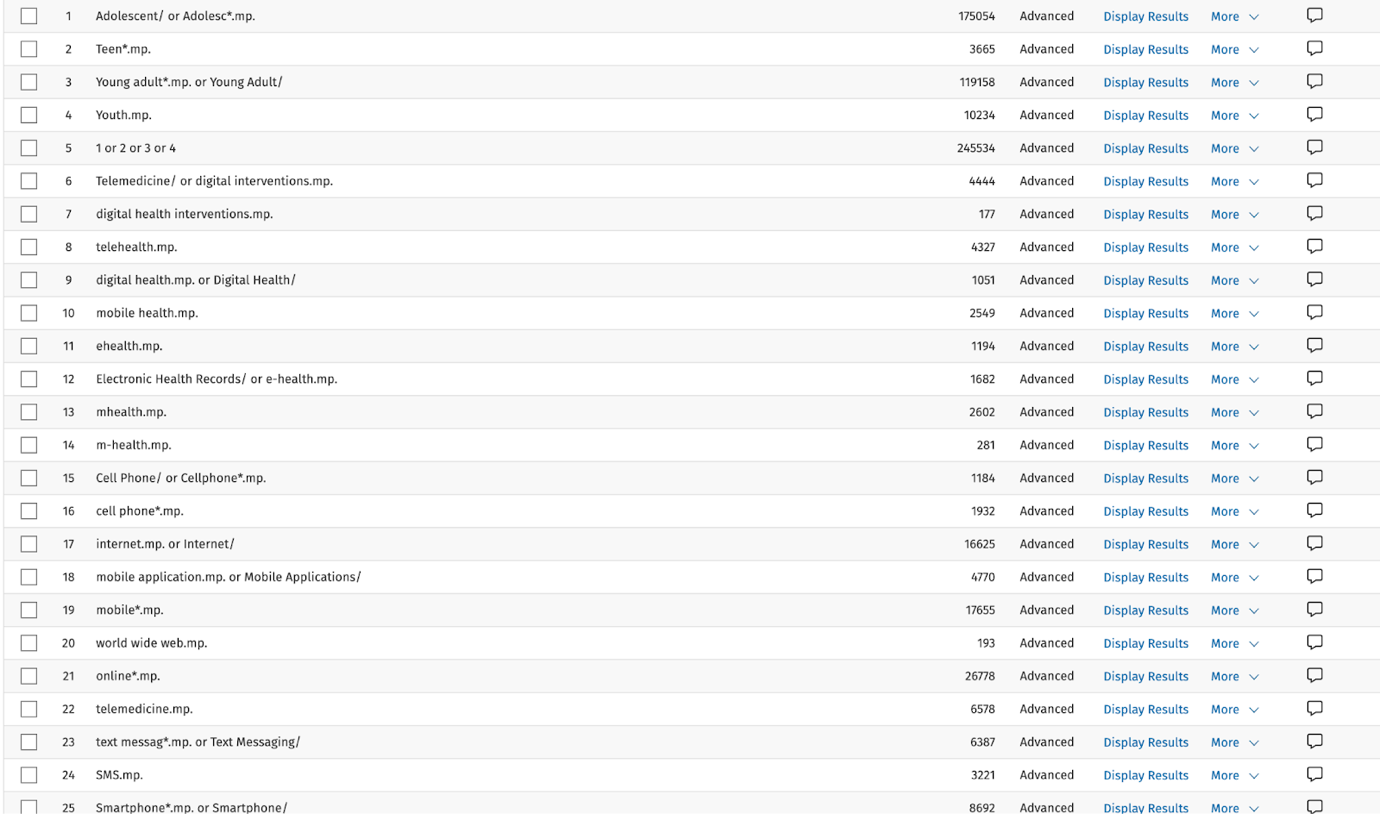


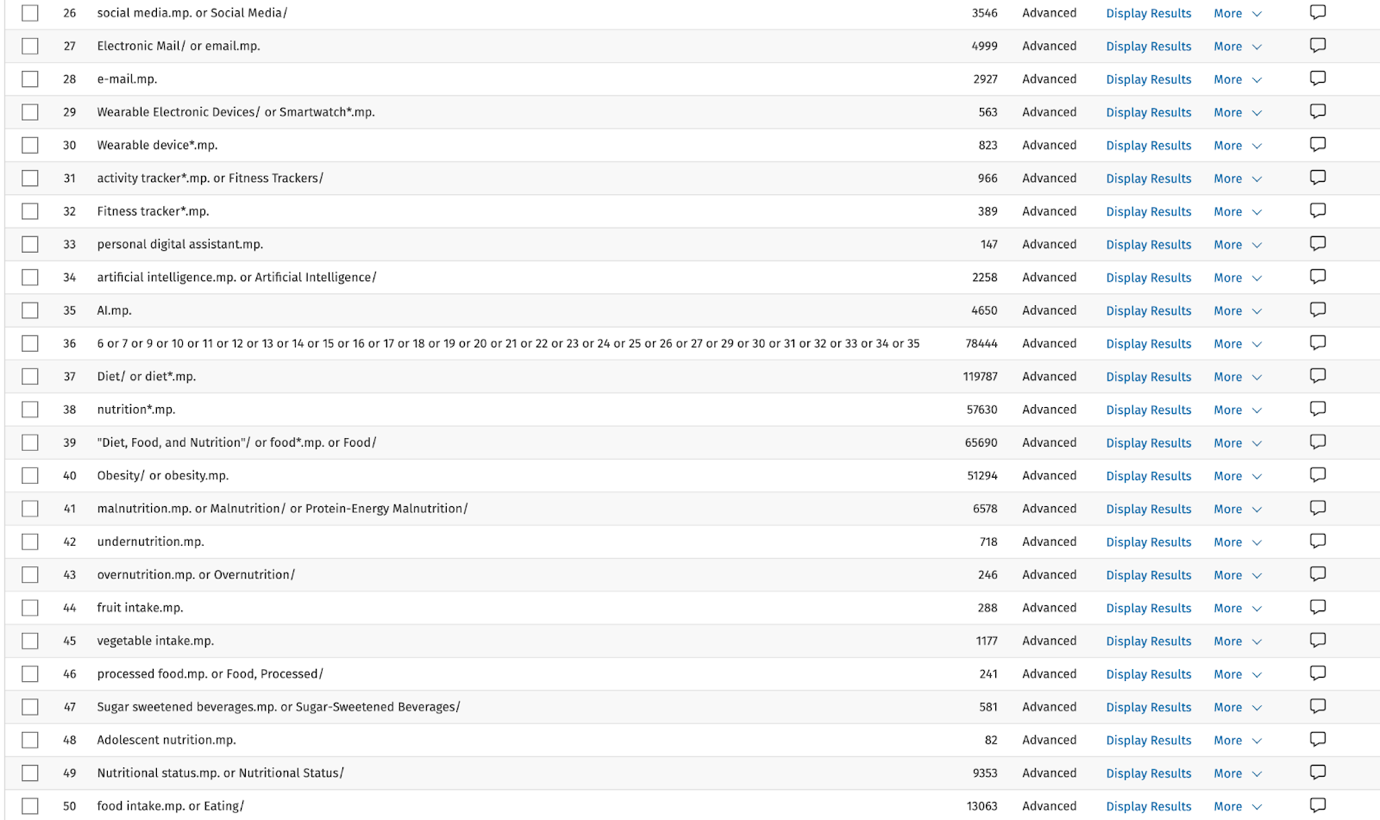


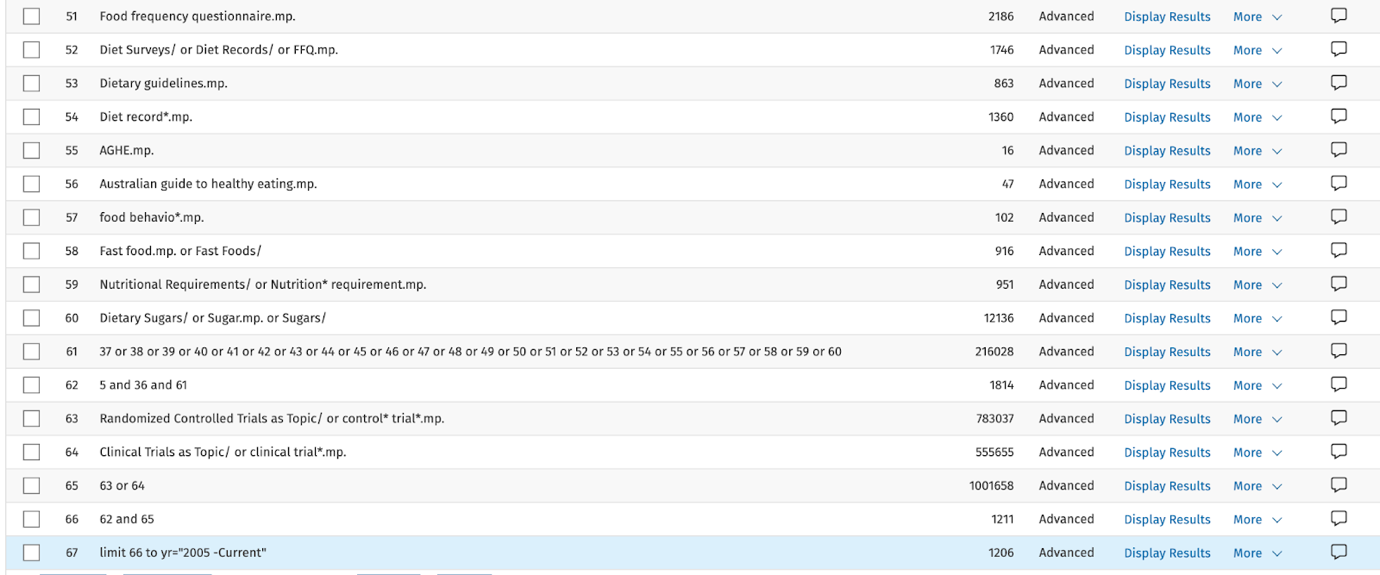


**Figure S5.** EMBASE Search Screenshot


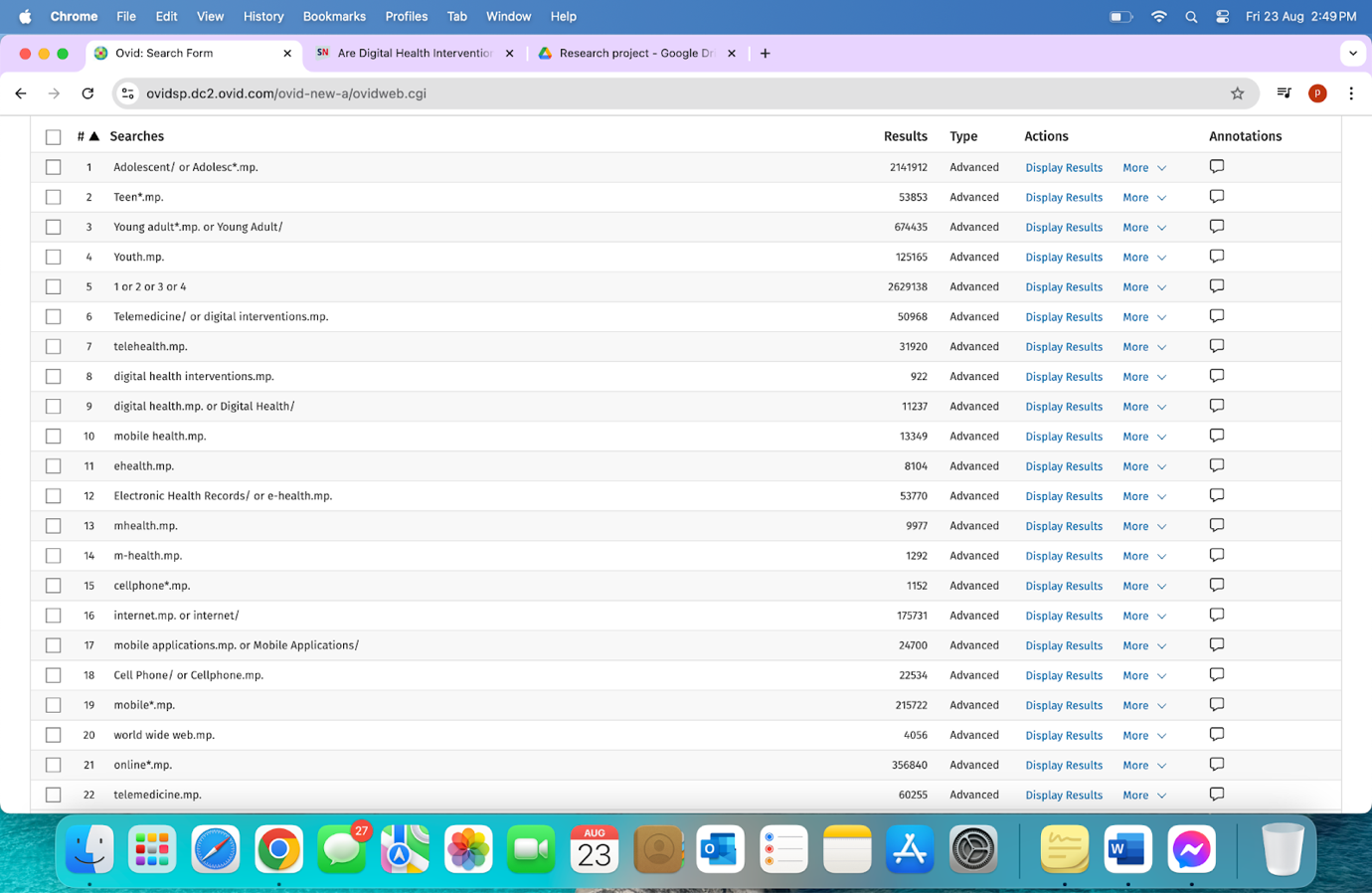


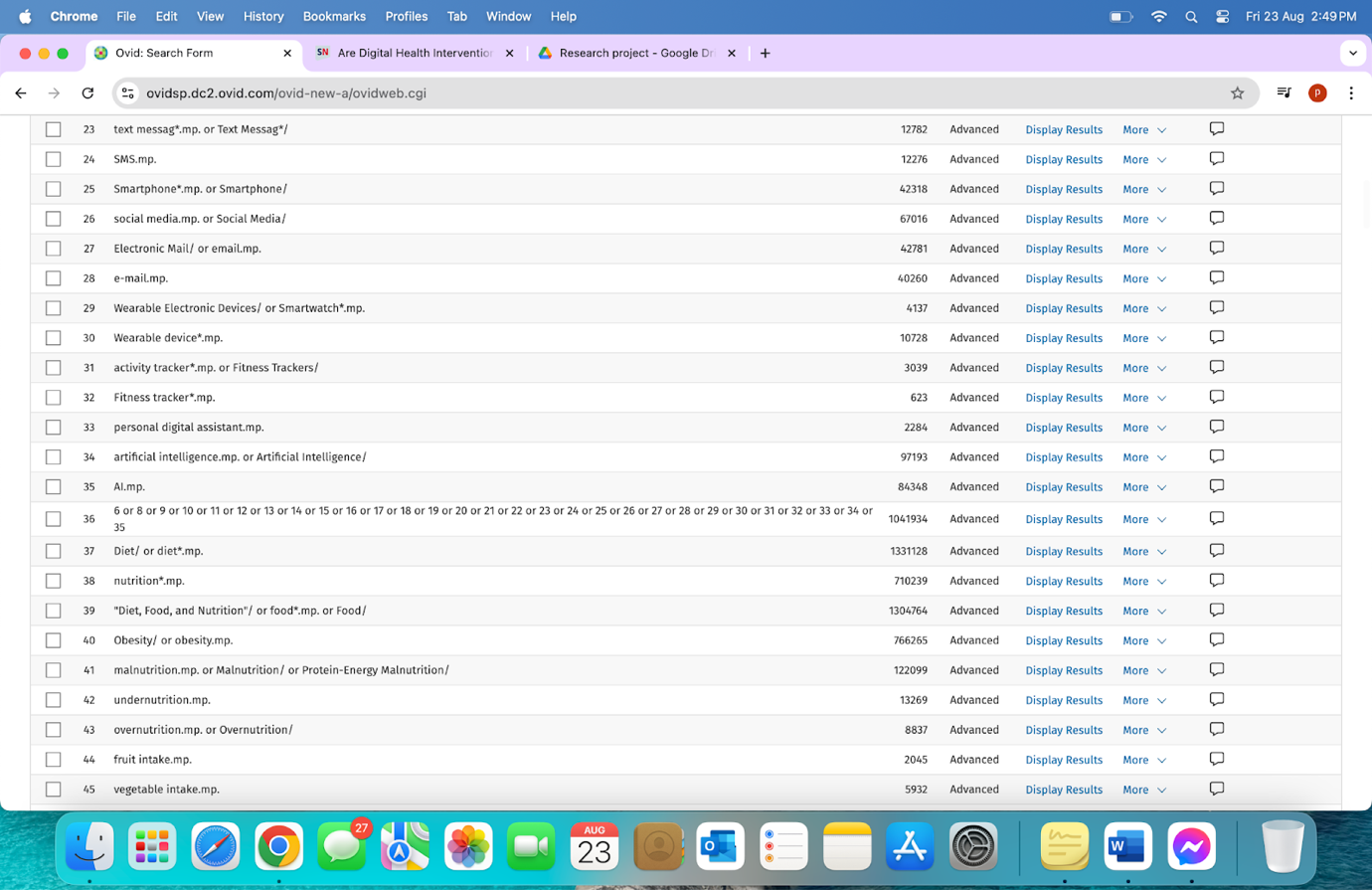


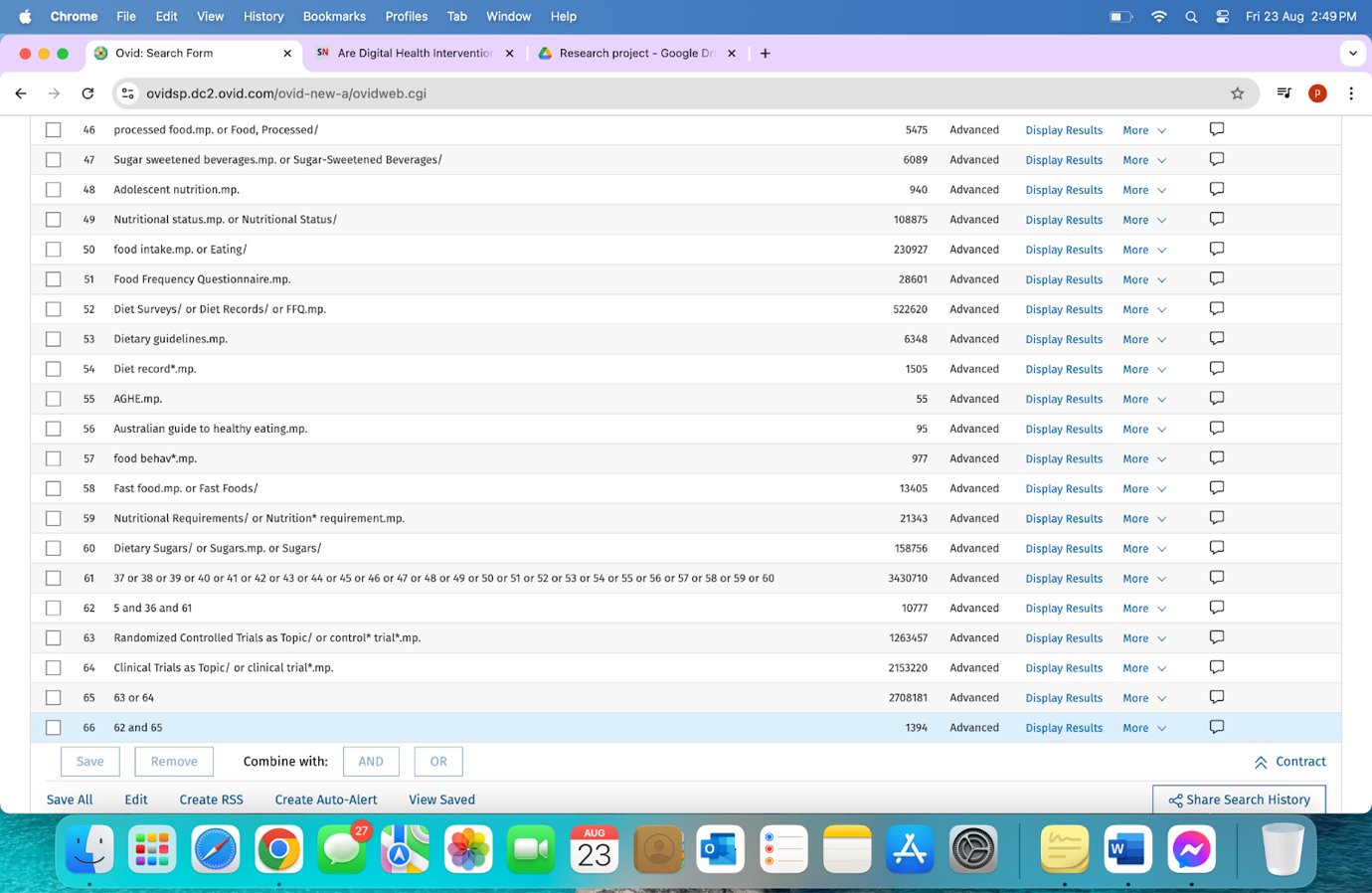


**Figure S6.** AMED Search Screenshot


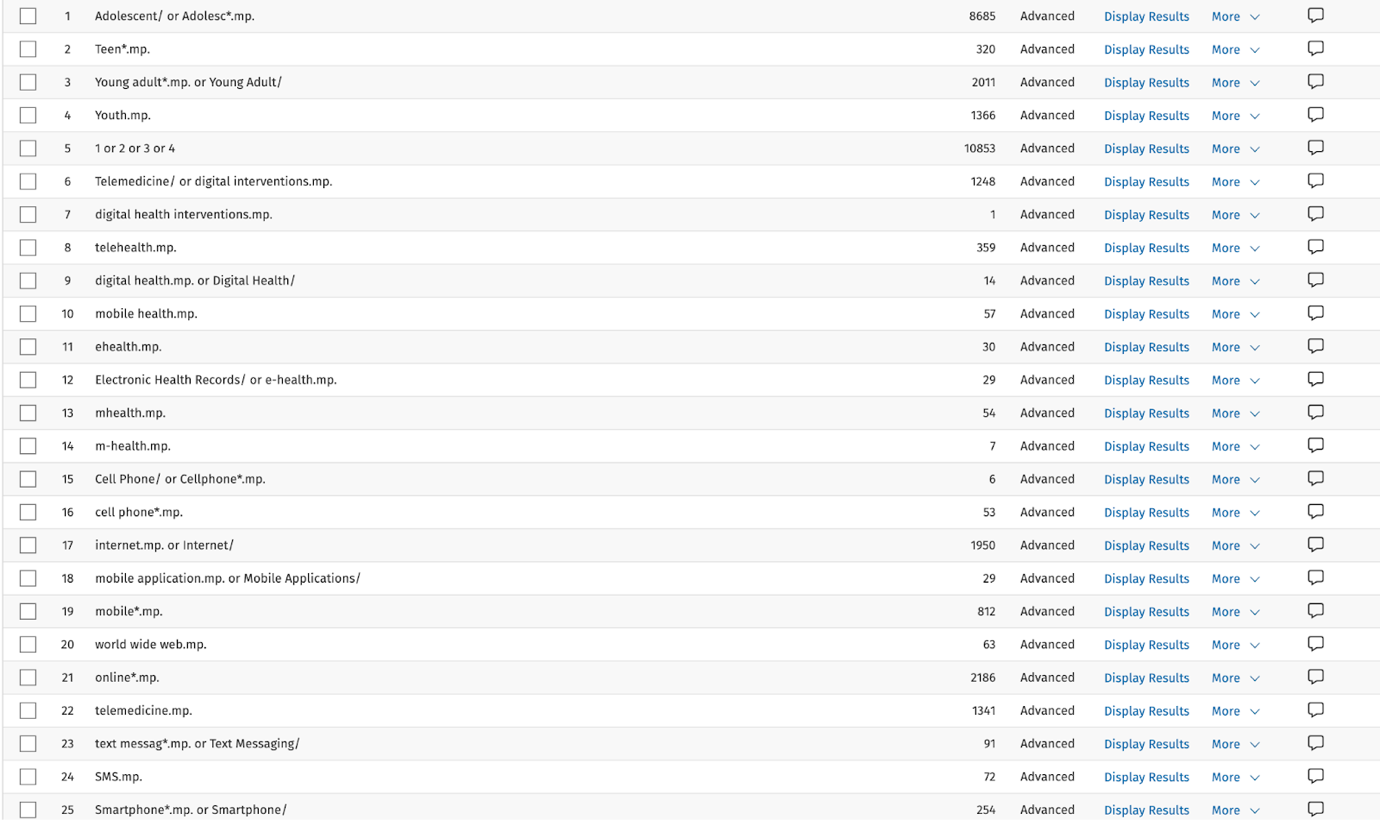


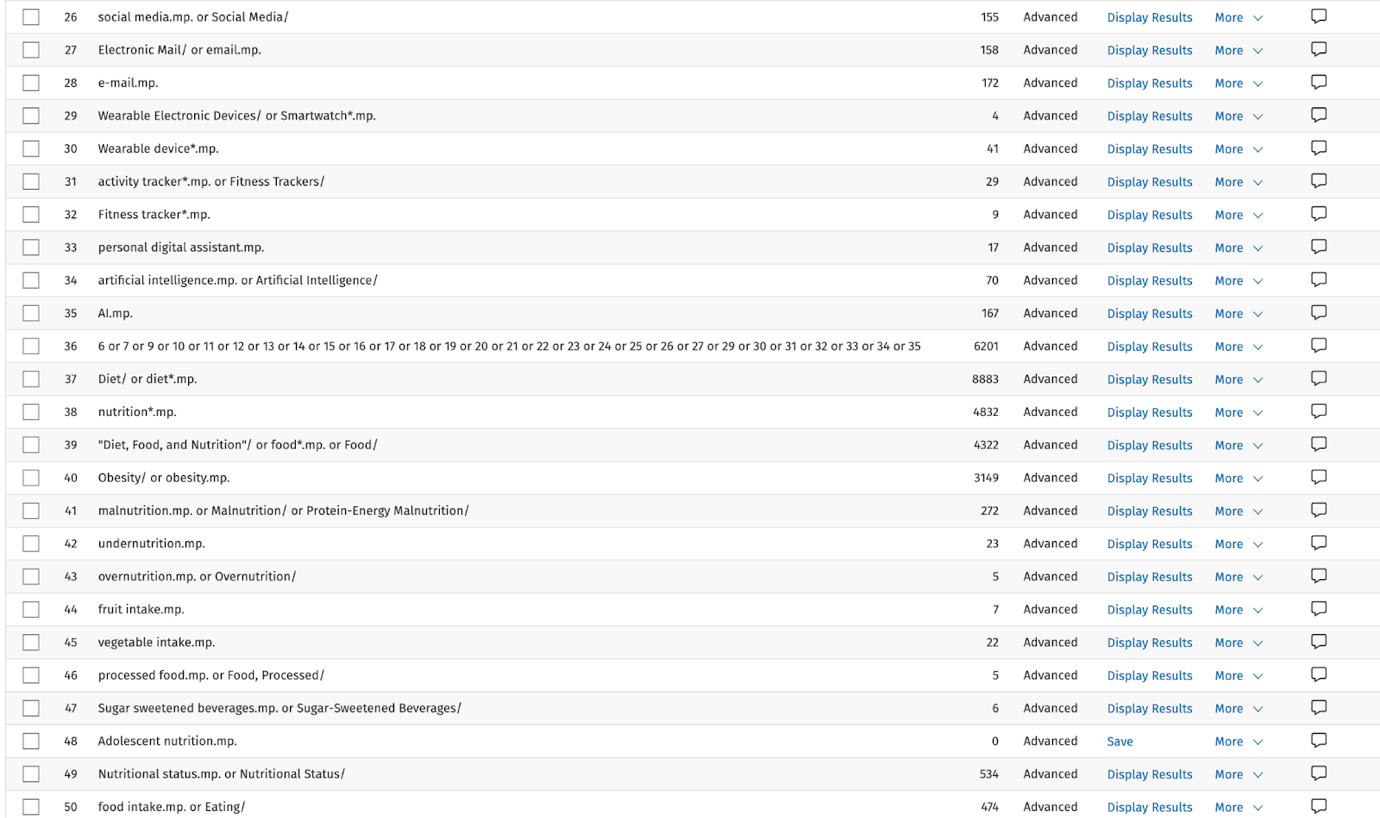


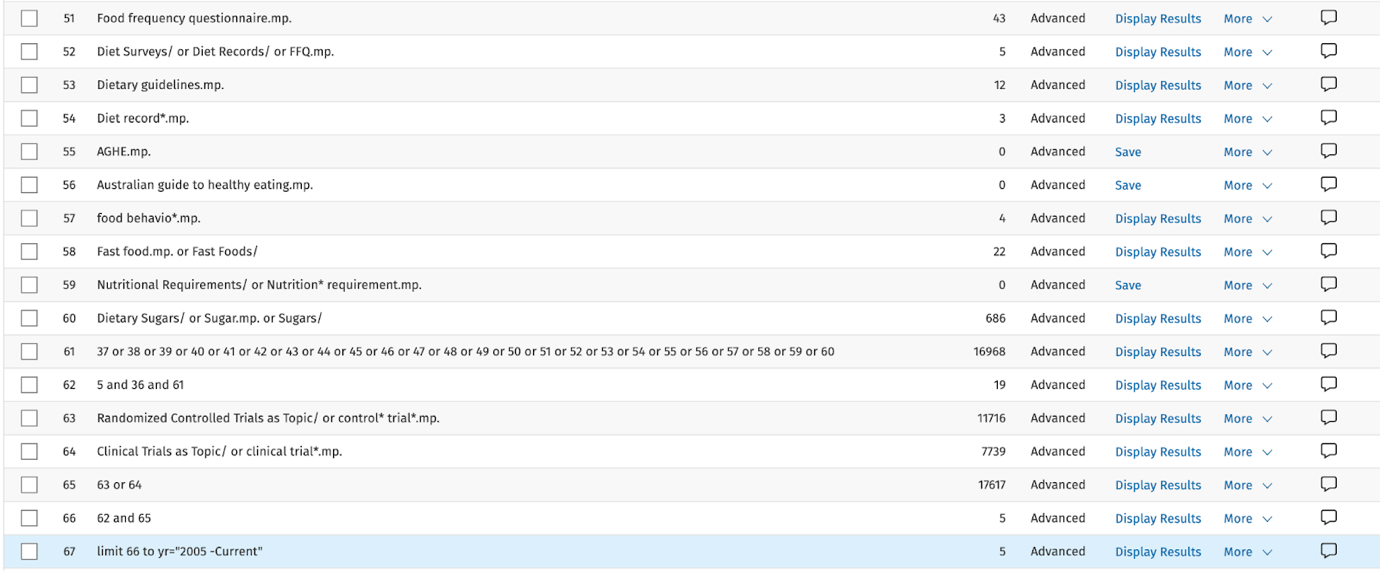


**Figure S7.** Scopus Search Screenshot


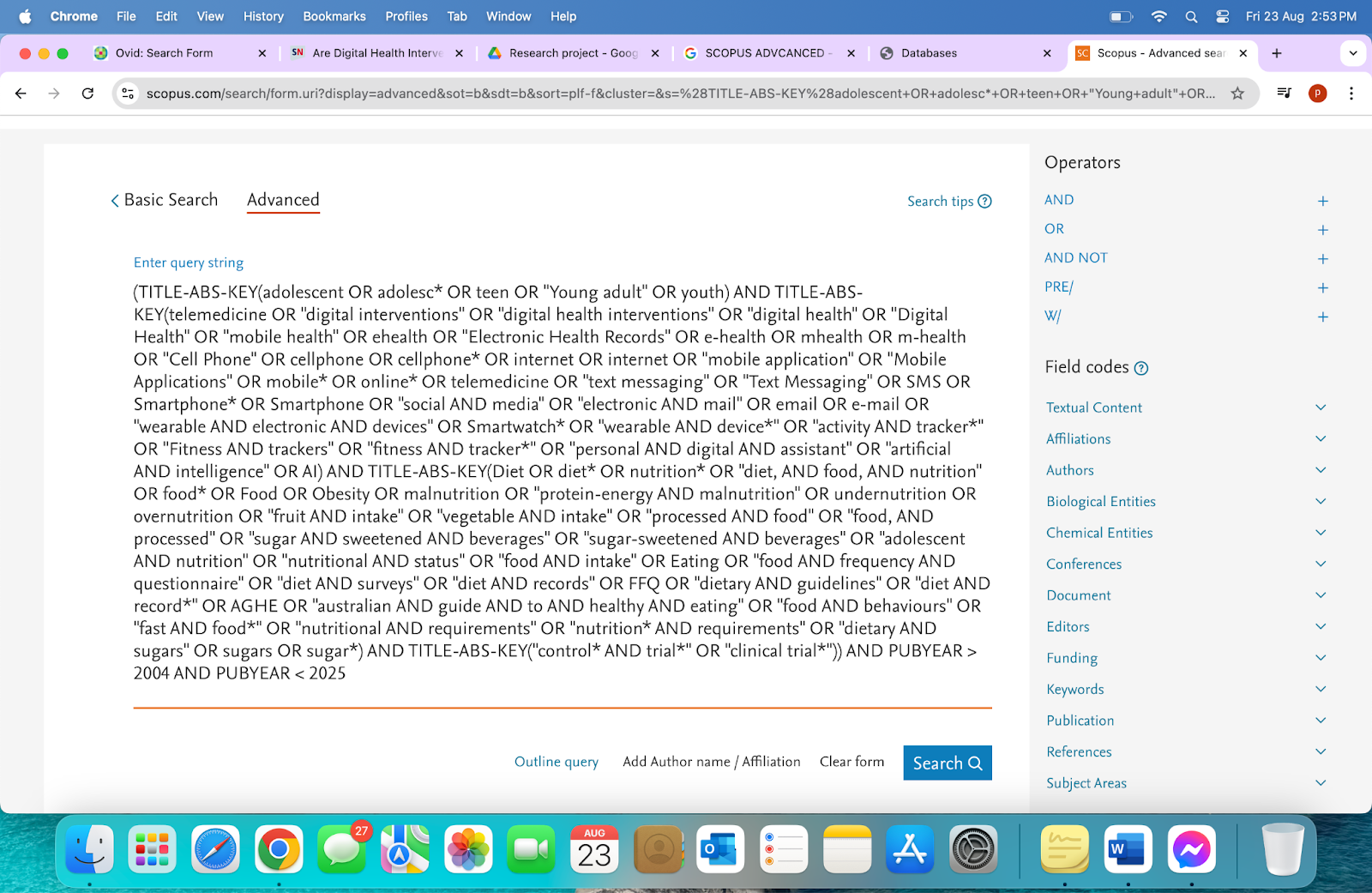


**Figure S8.** Web of Science Search Screenshot


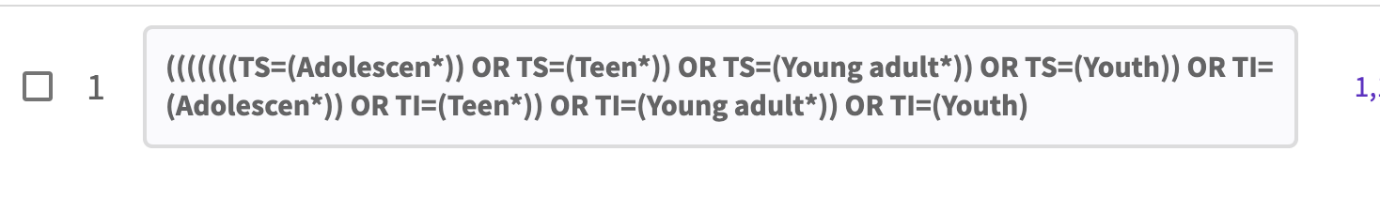


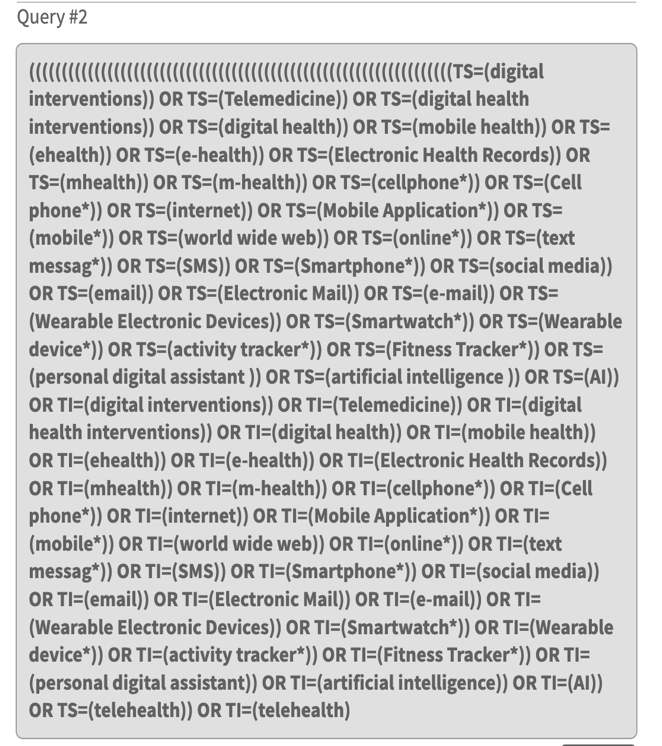

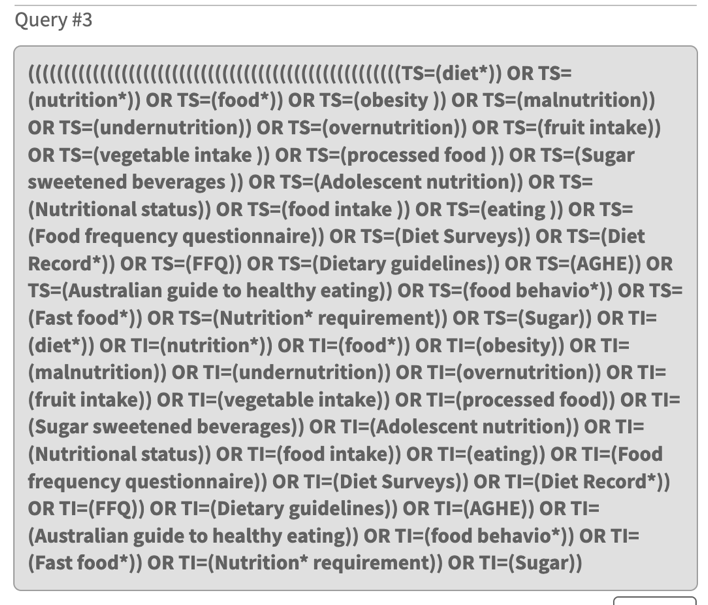


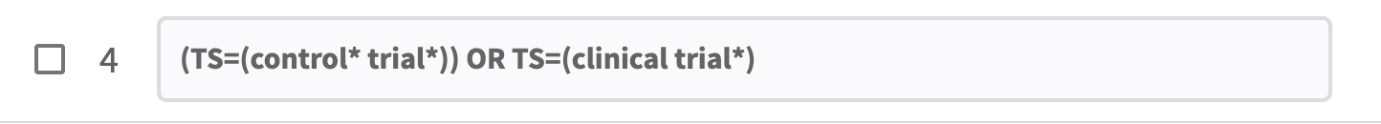


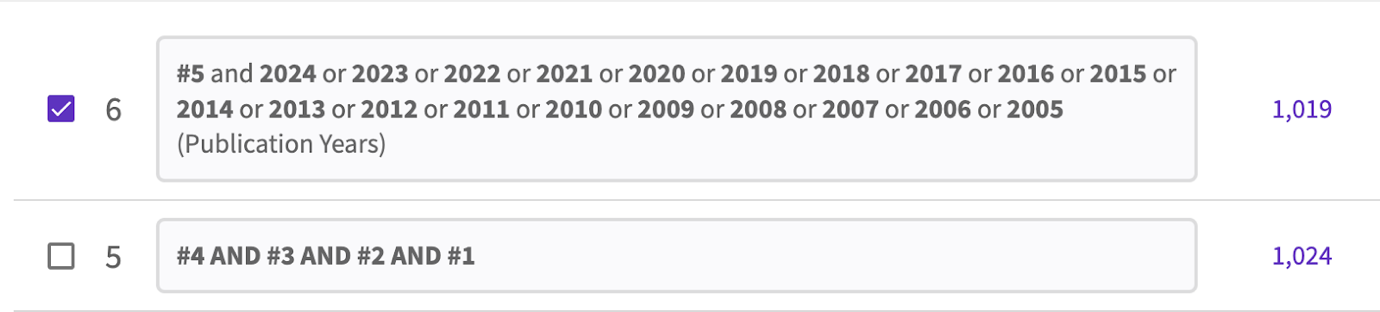


**Figure S9.** Informit Search Screenshot


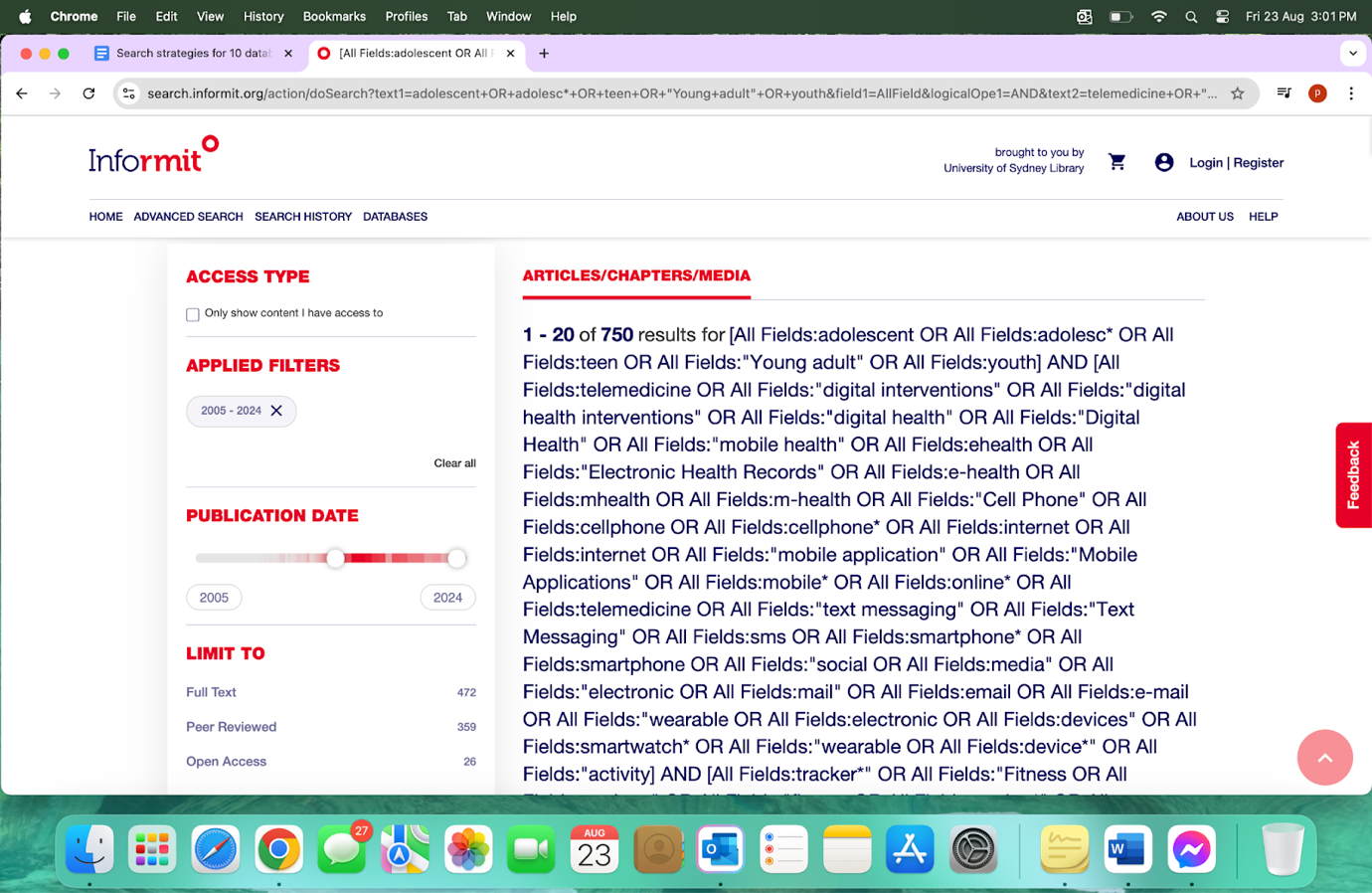


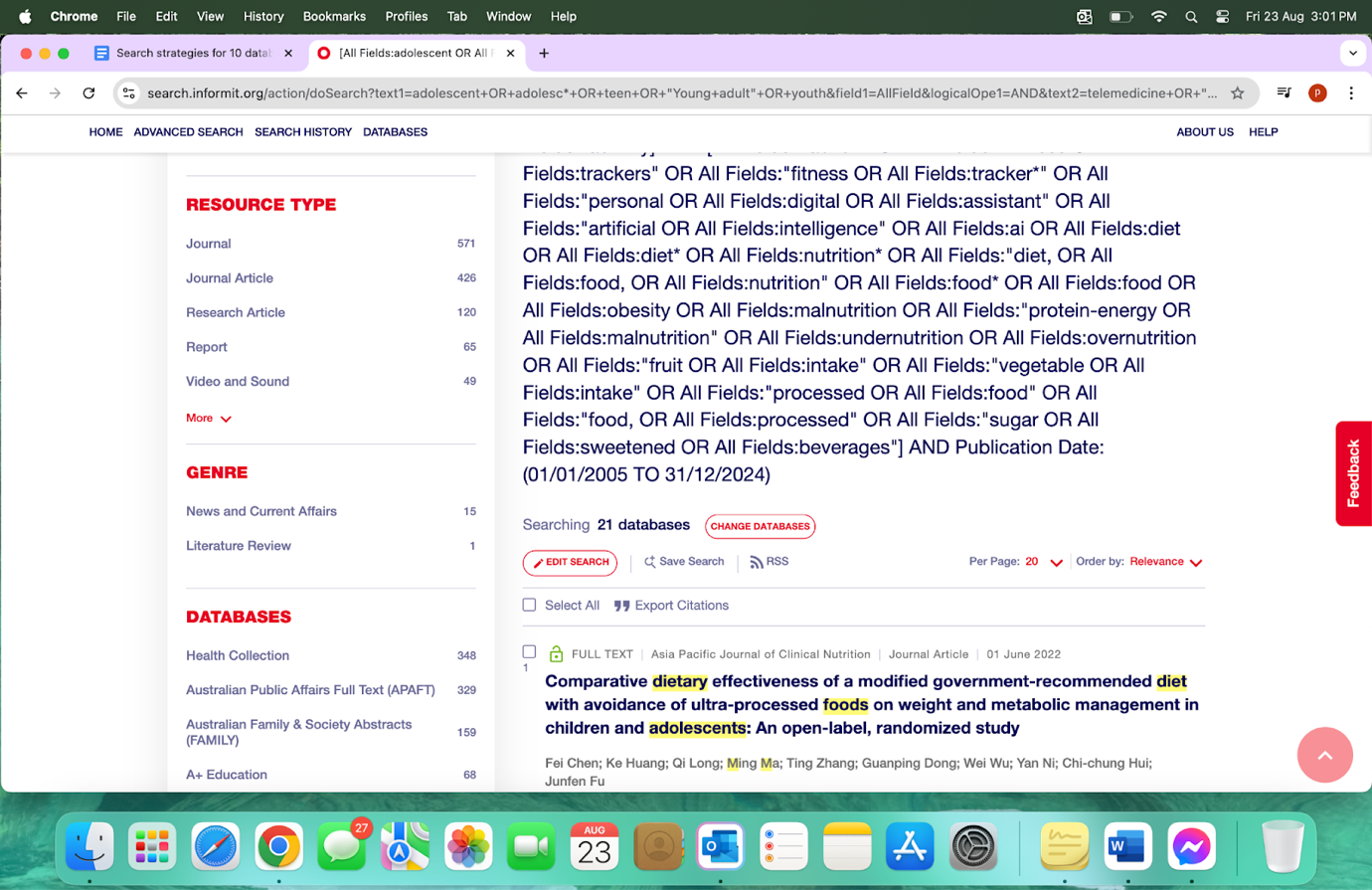


**Figure S10.** CINAHL Search Screenshot


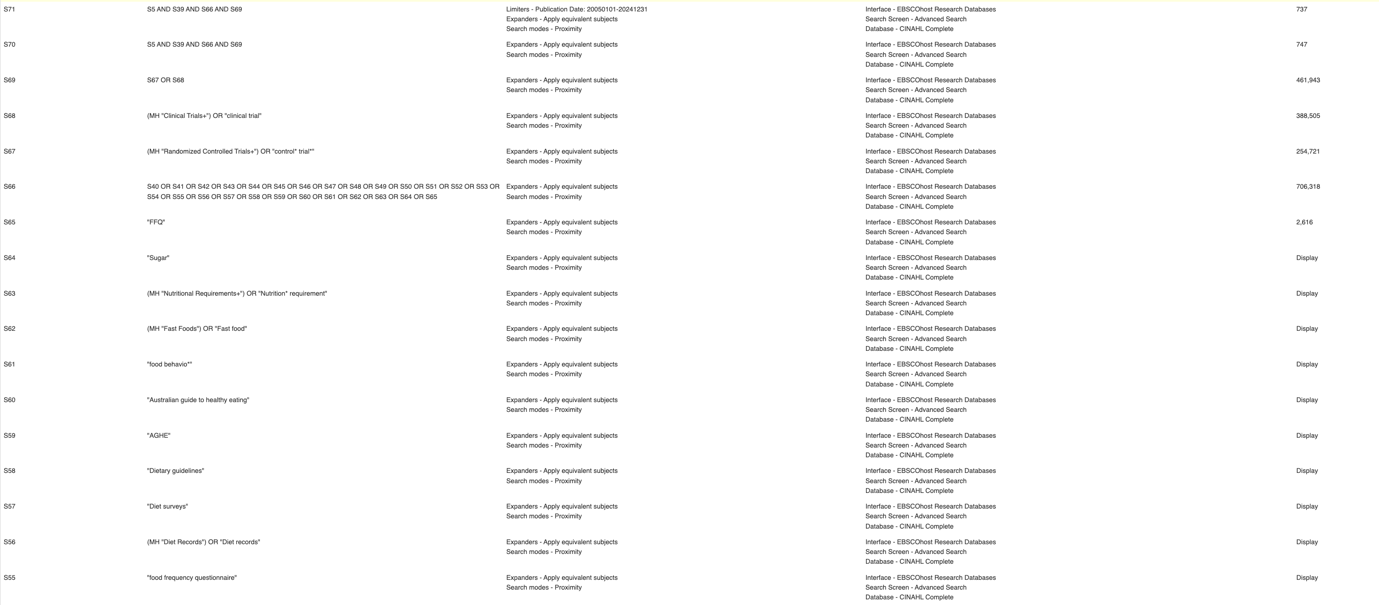


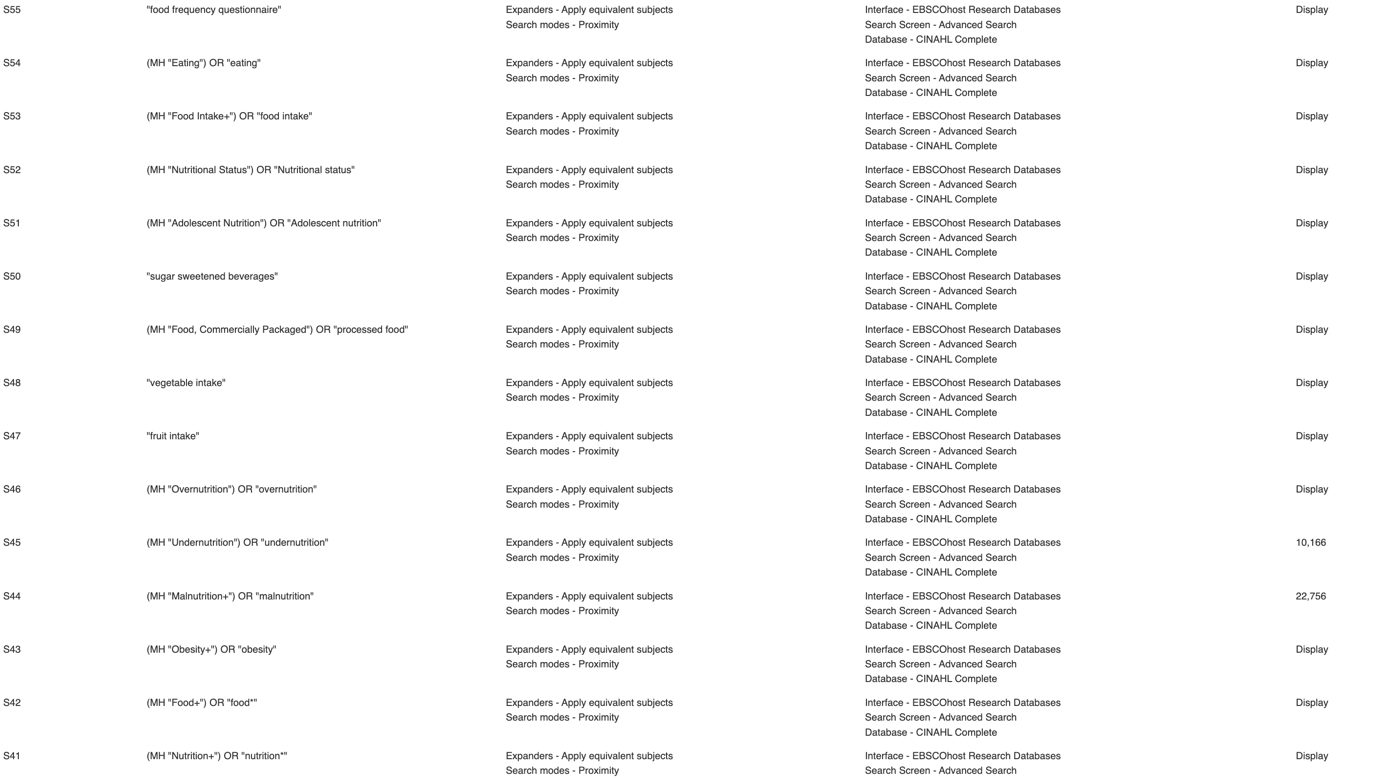


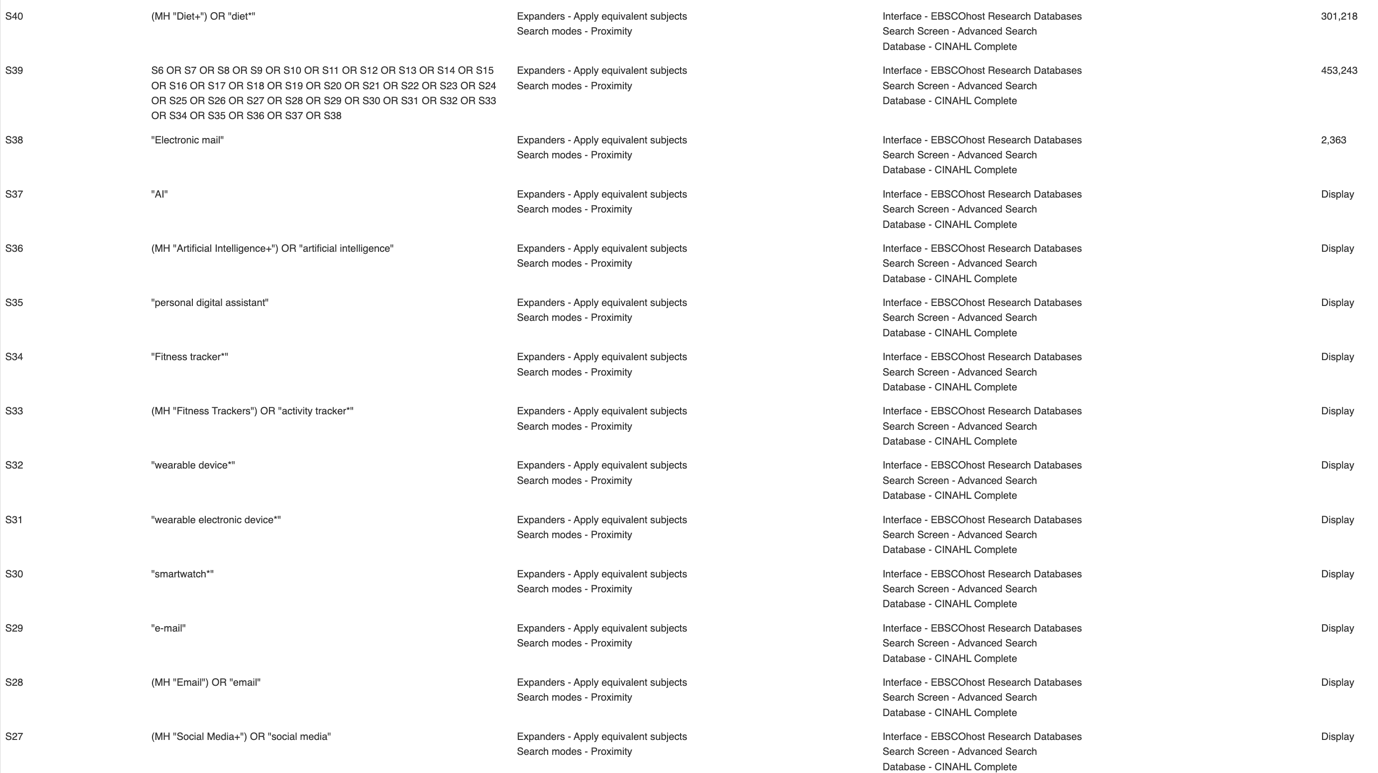


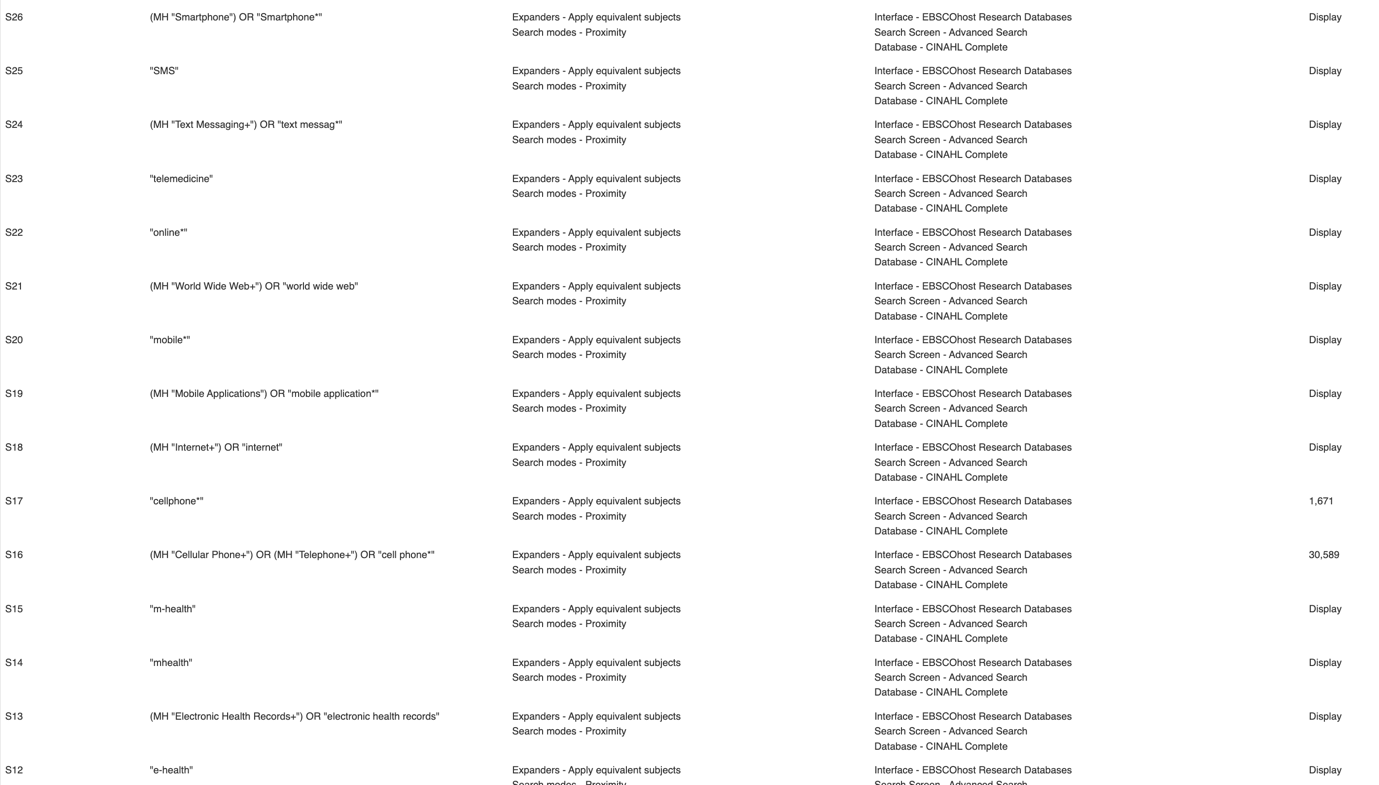

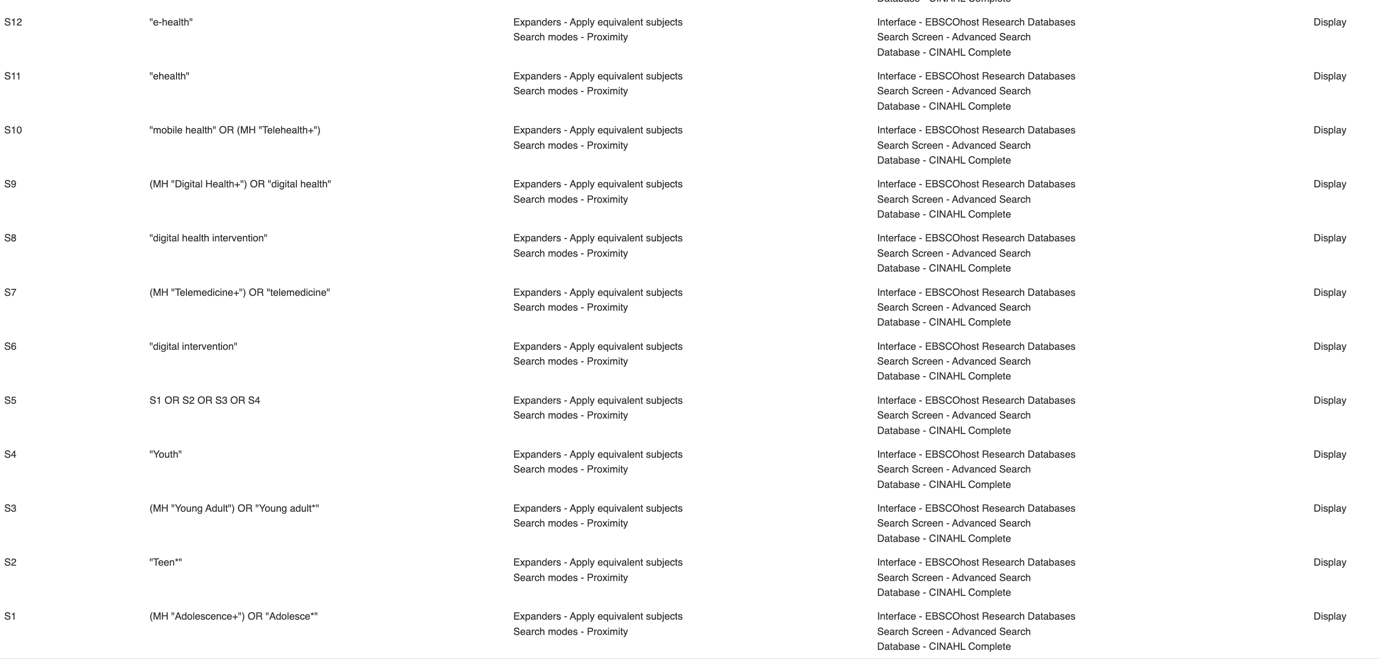


**Table S8.** Reasons for the exclusion of articles from full-text screening (n=53).

| **Article title** | **Reason for exclusion** |
| --- | --- |
| 01 Pilot Intervention with Adolescents to Increase Knowledge and Consumption of Folate-rich Foods | Conference abstract |
| A group randomized controlled trial integrating obesity prevention and control for postpartum adolescents in a home visiting program | Study design ineligible |
| A Mobile Phone App Intervention Targeting Fruit and Vegetable Consumption: The Efficacy of Textual and Auditory Tailored Health Information Tested in a Randomized Controlled Trial | Patient population ineligible |
| A multilevel, multicomponent childhood obesity prevention group-randomized controlled trial improves healthier food purchasing and reduces sweet-snack consumption among low-income African-American youth | Lack of usual care control group |
| A pilot text messaging intervention for adolescents and young adults recently off treatment for cancer | Conference abstract |
| A randomised control trial to evaluate the online delivery of the Better Health Program for children aged 7-13 years who have overweight or obesity in Victoria | Patient population ineligible |
| A randomized controlled feasibility trial of metacognitive training with adolescents receiving treatment for anorexia nervosa | Does not report nutrition outcome |
| A randomized trial comparing diet and delivery strategies for weight management in adolescents with intellectual disabilities | Does not report nutrition outcome |
| A theory-based online health behaviour intervention for new university students (U@Uni): results from a randomised controlled trial | Patient population ineligible |
| A trial of a six-month sugar-sweetened beverage intervention in secondary schools from a socio-economically disadvantaged region in Australia | Study design ineligible |
| An App Alone or With a Coach as a Comparable Approach to In-Person Treatment in Youth With Obesity | Conference abstract |
| An mHealth intervention for the treatment of patients with an eating disorder: A multicenter randomized controlled trial | Not able to contact author |
| Boy Scout 5-a-Day Badge: outcome results of a troop and Internet intervention | Lack of usual care control group |
| Changes in food addiction scores over the course of weight management interventions in a diverse sample of adolescents with overweight and obesity | Conference abstract |
| Cognitive-behavioural therapy for adolescents with bulimic symptomatology: The acceptability and effectiveness of internet-based delivery | NOT RCT |
| Comparison of an interviewer-administered with an automated self-administered 24 h (ASA24) dietary recall in adolescents | Study design ineligible |
| Creating action plans in a serious video game increases and maintains child fruit-vegetable intake: a randomized controlled trial | Study design ineligible |
| Differences between two types of nutritional education programs | Conference abstract |
| EDNET - Eating Disorders Diagnostic and Treatment Network | NOT RCT |
| Effect of a Multicomponent mHealth Intervention on the Composition of Diet in a Population with Overweight and Obesity—Randomized Clinical Trial EVIDENT 3 | Patient population ineligible |
| Effect of an interactive mobile health support system and daily weight measurements for pediatric obesity treatment, a 1-year pragmatical clinical trial | Does not report nutrition outcome |
| Effectiveness of Web-Based Health Education and Consultation on Health Promotion Behaviors of Adolescents | Not able to contact author |
| Effects of Advice to Drink 8 Cups of Water per Day in Adolescents With Overweight or Obesity: A Randomized Clinical Trial | Does not report nutrition outcome |
| Efficacy and acceptability of self‐monitoring via a smartphone application versus traditional paper records in an intensive outpatient eating disorder treatment setting | Patient population ineligible |
| Efficacy of an internet-based behavioral weight loss program for overweight adolescent African-American girls | Study design ineligible |
| Evaluation of a web-based program promoting healthy eating and physical activity for adolescents: teen choice: food and fitness | Study design ineligible |
| Exposure effects to unfamiliar food advertisements on YouTube: A randomized controlled trial among adolescents | Does not report nutrition outcome |
| Extending obesity care beyond the office doors using telemedicine health coaches. A pilot test randomized trial | Conference abstract |
| Healthy lifestyle management of pediatric obesity with a hybrid system of customized mobile technology: the pediafit pilot project | NOT RCT |
| Healthy outcome for teens (HOT project): Change in social cognitive theory constructs in a randomized control trial | Conference abstract |
| Imagine HEALTH: results from a randomized pilot lifestyle intervention for obese Latino adolescents using Interactive Guided Imagery | Lack of usual care control group |
| Impact of a Web-Based Nutrition Intervention on Eating Behaviors and Body Size Preoccupations among Adolescents | Study design ineligible |
| Mobile health (mHealth) intervention called BodiMojo using text messaging aimed at healthy lifestyles for youth with diabetes (DM): a pilot Randomized Controlled Trial (RCT) | Conference abstract |
| Motivational support programme to enhance health and well‐being and promote weight loss in overweight and obese adolescents: A randomized controlled trial in Turkey | Does not report nutrition outcome |
| Multiple Behavior Changes in Diet and Activity: A Randomized Controlled Trial Using Mobile Technology | Patient population ineligible |
| P34 Development and Evaluation of an Informal SMS-based Intervention to Promote Healthy Lifestyle Behaviors in Adolescents | Conference abstract |
| Pilot evaluation of the HELENA (Healthy Lifestyle in Europe by Nutrition in Adolescence) Food-O-Meter, a computer-tailored nutrition advice for adolescents: a study in six European cities | Lack of usual care control group |
| Playing with fruit: an experimental study to test the effectiveness of an online memory advergame to promote children's fruit consumption | Patient population ineligible |
| Randomized test of an implementation intention-based tool to reduce stress-induced eating | Patient population ineligible |
| Short-term efficacy of a web-based computer-tailored nutrition intervention: main effects and mediators | Patient population ineligible |
| Texting your way to healthier eating? Effects of participating in a feedback intervention using text messaging on adolescents' fruit and vegetable intake | NOT RCT |
| The Effect of the Promotion of Vegetables by a Social Influencer on Adolescents' Subsequent Vegetable Intake: a Pilot Study | Lack of usual care control group |
| The impact of Curtin University's activity, food and attitudes program on physical activity, sedentary time and fruit, vegetable and junk food consumption among overweight and obese adolescents: a waitlist controlled trial | NOT RCT |
| The outcomes of thirsty?: Choose water! determining the effects of a behavioural and an environmental intervention on water and sugar sweetened beverage consumption in adolescents: A randomised controlled trial | Lack of usual care control group |
| The use of a text-messaging program during weight loss treatment: baseline results regarding fruits and vegetables intake, screen time and physical activity in obese children and adolescents | Conference abstract |
| Use of new technologies for the follow-up in adolescent obesity; mobile health intervention (MHI) a randomized controlled trial | Conference abstract |
| Use of text messaging for monitoring sugar-sweetened beverages, physical activity, and screen time in children: a pilot study | Patient population ineligible |
| Using a website to build community and enhance outcomes in a group, multi-component intervention promoting healthy diet and exercise in adolescents | NOT RCT |
| Feasibility of Using an Artificial Intelligence-based Telephone Application for Dietary Assessment and Nudging to Improve the Quality of Food Choices of Female Adolescents in Vietnam: Evidence from a Randomized Pilot Study. | Lack of usual care control group |
| A mentor-led text-messaging intervention increases intake of fruits and vegetables and goal setting for healthier dietary consumption among rural adolescents in kentucky and North Carolina, 2017 | NOT RCT |
| Effects of integrating pedometers, parental materials, and E-mail support within an extracurricular school sport intervention | NOT RCT |
| Effects of a practice-focused nutrition intervention in Hungarian adolescents | NOT RCT |
| The Internet-based My Body, My Life: Body Image Program for adolescent girls improves body image and disordered eating | Conference abstract |

**Table S9. Characteristics of included studies *(n=20 articles reporting 19 studies)***

| **Author, year, country** | **Language** | **PROGRESS Plus characteristics measured at baseline** | **Recruitment** **setting** | **Sample size (n)** |
| --- | --- | --- | --- | --- |
| **(Bjerregaard et al., 2024), Denmark** | Danish | R: Danish  G: Both sex^1^  +: Age range (mean, SD): 14 (14^2^) | Community health centre | 7890 |
| **(Carfora et al., 2016), Italy** | Italian/ English | R: Italian  G: Both sex^1^  +: Age range (mean, SD): 13-19 (15, 1.53) | High school | 1056 |
| **(Chen et al., 2017; Chen et al., 2019), USA** | English | R: Chinese American  G: Both sex^1^  +: Age range (mean, SD): 13-18 (14.9^2^) | Community clinic in northern California | 40 |
| **(Doyle et al., 2008), USA** | English | R: White/ Black Hispanic  G: Both sex (62.5% Female, 37.5% Male)  E (Parents) : Less than high school/ High school graduate/ Some college or technical school/ College graduate/ Some graduate school/ Graduate/Professional degree  +: Age range (mean, SD): 12-18 (14.5, 1.7)  +: RF (Parents) - Married/remarried; Single/ divorced/ separated | Community health centre and school | 80 |
| **(Ghadam et al., 2023), Iran** | English | R: Iranian  S: Female  +: Age range (mean, SD): 10-19 (NR) | School | 176 |
| **(Heikkila et al., 2019), Finland** | Finnish | R: Finnish  G: Both sex (56% Female, 44% Male)  +: Age range (mean, SD): 16-20 (18, 1.4) | Sports club | 79 |
| **(Heinicke et al., 2007), Australia** | English | R: Caucasian, Indian, Asian  G: Female  E (Parents): Average Victorian socioeconomic status  SS: Average Victorian socioeconomic status  +: Age range (mean, SD): 12-18 (14.4, 1.48) | School | 83 |
| **(Jones et al., 2008), USA** | English | R: White/ Black/ Hispanic/ Latino/ Mexican  G: Both sex (73% female, 27% male)  +: Age range (mean, SD): NR (15.1 ^2^) | High school | 105 |
| **(Lana et al., 2014), Spain and Mexico** | NR | R: Spanish/ Mexican  G: Both sex (54.8% female, 46.2% male)  +: Age range (mean, SD): 12-16 (NR) | School | 2001 |
| **(Likhitweerawong et al., 2021), Thailand** | Thai | R: Thai  G: Both (31% Female, 69% Male)  +: Age range (mean, SD): 10-15 (Intervention: 13.11, 1.99; Control: 12.81, 1.79) | School | 77 |
| **(Manzano-Felipe et al., 2024), Spain** | Spanish | R: Spainish  G: Both sex (51%, Female, 49% Male)  +: Age range (mean, SD): 11-17 (Intervention: 13.31, 0.97; Control: 13.35 (0.98) | School | 305 |
| **(Nollen et al., 2014), USA** | English | POR: Economically disadvantaged neighbourhoods  R: African American/Bi or multi-racial/ American Indian/ Alaska Native/ Asian/ Hispanic/ Latina  G: Female  SS: Low-income  +: Age range (mean, SD): 9-14 (NR) | After-school program | 51 |
| **(Patrick et al., 2013), USA** | English | R: American Indian/ African American/ Hispanic/ Asian/ Pacific Islander  G: Both sex (61.5% female, 38.5% male)  SS: Economically diverse  +: Age range (mean, SD): 12-16 (NR) | Waiting rooms and local media at pediatric practices | 101 |
| **(Shatwan et al., 2023), Saudi Arabia** | Arabic | G: Both sex (54% Female, 46% Male)  E (Parents) : Paternal & maternal- High school or lower/ Bachelor/ Postgraduate  SS: Income-(USD) Less than $5000/ $5000 to <$10,000/ $10,000 to <$20,000/ >$20,000  Employment (Paternal & maternal)- Unemployed/ Employed/ Retired  +: Age range (mean, SD): 13-18 (15.1, 1.6) | School | 104 |
| **(Sivasamy, 2020), India** | Indian | G: Both sex^1^  +: Age range (mean, SD): 18 (18,0.49) | School | 42 |
| **(Tugault-Lafleur et al., 2023), Canada** | English | R: White/ European/ Aboriginal/ East or Southeast Asian/ South Asian/ Mixed (White and Aboriginal)/ Mixed (other combinations  G: Both sex (61.5% female, 38.5% male)  E (Parents) High school degree or lower/ Attended college/ Bachelors’ degree or above  SS: (CAD) <$50,000/ $50,000 to $99,999/ $100,000 to $149,999/ ≥$150,000  +: Age range (mean, SD): 10-17 (Intervention: 12.8, 2.2; Control: 13.1, 2.3)  + RF (Parents)- Married or common-law/ Single/ separated/ widowed | Hospital | 214 |
| **(van Egmond-Fröhlich et al., 2006), Germany** | German | G: Both sex^1^  +: Age range (mean, SD): 9-16 (13.3, 1.8) | Hospital | 521 |
| **(Vermeiren et al., 2021) , Belgium** | English | R: Belgian  G: Both sex (Inpatients: 58% female, 42% male; Outpatients: 55% female, 45% male outpatients)  +: Age range (mean, SD): 8-18 (Inpatient: 14.3^2^, Outpatient: 11.9^2^) | Hospital | Inpatients: 144  Outpatients: 115  Total: 259 |
| **(Wambach et al.), USA** | English | R: Black/ White/ non-Hispanic/ White/ More than one race/ Non-Hispanic/ More than one race/ Hispanic and Unknown/ Hispanic  G: Female  SS: (USD) Less than $25,000/ $25,001 to $55,000/ $55,001 to $85,000  +: Age range (mean, SD): 15-19 (17.88, 1.06) | Community health centre | 62 |

[^[1]^](https://auc-word-edit.officeapps.live.com/we/wordeditorframe.aspx?ui=en-US&rs=en-US&wopisrc=https%3A%2F%2Funisydneyedu-my.sharepoint.com%2Fpersonal%2Fllam7876_uni_sydney_edu_au%2F_vti_bin%2Fwopi.ashx%2Ffiles%2F264c450e2e1449a7a67951e196e426e9&wdenableroaming=1&mscc=1&wdodb=1&hid=22AD5BA1-A01F-3000-E43A-5316990DDB26.0&uih=sharepointcom&wdlcid=en-US&jsapi=1&jsapiver=v2&corrid=4e97dc2e-ef5a-f92e-47f2-cb8a387ce415&usid=4e97dc2e-ef5a-f92e-47f2-cb8a387ce415&newsession=1&sftc=1&uihit=docaspx&muv=1&cac=1&sams=1&mtf=1&sfp=1&sdp=1&hch=1&hwfh=1&dchat=1&sc=%7B%22pmo%22%3A%22https%3A%2F%2Funisydneyedu-my.sharepoint.com%22%2C%22pmshare%22%3Atrue%7D&ctp=LeastProtected&rct=Normal&wdorigin=ItemsView&wdhostclicktime=1729406257594&instantedit=1&wopicomplete=1&wdredirectionreason=Unified_SingleFlush#_ftnref1) No sex ratio reported

[^[2]^](https://auc-word-edit.officeapps.live.com/we/wordeditorframe.aspx?ui=en-US&rs=en-US&wopisrc=https%3A%2F%2Funisydneyedu-my.sharepoint.com%2Fpersonal%2Fllam7876_uni_sydney_edu_au%2F_vti_bin%2Fwopi.ashx%2Ffiles%2F264c450e2e1449a7a67951e196e426e9&wdenableroaming=1&mscc=1&wdodb=1&hid=22AD5BA1-A01F-3000-E43A-5316990DDB26.0&uih=sharepointcom&wdlcid=en-US&jsapi=1&jsapiver=v2&corrid=4e97dc2e-ef5a-f92e-47f2-cb8a387ce415&usid=4e97dc2e-ef5a-f92e-47f2-cb8a387ce415&newsession=1&sftc=1&uihit=docaspx&muv=1&cac=1&sams=1&mtf=1&sfp=1&sdp=1&hch=1&hwfh=1&dchat=1&sc=%7B%22pmo%22%3A%22https%3A%2F%2Funisydneyedu-my.sharepoint.com%22%2C%22pmshare%22%3Atrue%7D&ctp=LeastProtected&rct=Normal&wdorigin=ItemsView&wdhostclicktime=1729406257594&instantedit=1&wopicomplete=1&wdredirectionreason=Unified_SingleFlush#_ftnref4) No standard deviation reported

**Table S10. Intervention characteristics *(n=20 articles reporting 19 studies)***

| **Author, year, country** | **Intervention principle** | **Digital modality of intervention** | **Intervention details and exposure** | **Control** | **Duration of intervention** | **Nutrition outcomes**   - **Primary/ Secondary** | **Digital determinants of health (data poverty and information asymmetry, usability, perceived usefulness, interactivity, digital literacy, accessibility, affordability, algorithmic bias, and technology personalisation)** | **Not addressing DDoH** |
| --- | --- | --- | --- | --- | --- | --- | --- | --- |
| **(Bjerregaard et al., 2024), Denmark** | Chatbox health education | SMS | 2 x 2 x 2 factorial trial + control  1. 12-week intervention with 4 weeks for each of the 3 targeted SMS programs: increasing the adolescents’ fish intake, increasing their FV intake, or reducing their SSB intake  2. 9 groups: differ by involve mother, friends, and tailored target or not | Participants did not participate in any program | 12 weeks | ‘mini Healthy Eating Index’ (mini-HEI)  Fish intake  FV intake  SSB intake   - Primary | - Perceived usefulness: Tailored dietary outcome base on risk profile - Affordability: SMS reminder for free - Technology personlisation: Text messages are designed in a manner that would be appealing to the age group | Accessibility: Need a device to receive SMS, exclude population with disability  Algorithmic bias: N/A |
| **(Carfora et al., 2016), Italy** | Affective message intervention | SMS | 3 arm RCT – Affective (A), Instrumental (I) or Control (C)  A: one message each day over a 2-week period, focusing on positive affective consequences of FV intake.  I: one message each day over a 2-week period, focusing on physical benefits of FV intake.  C: no text messages | No text messages | 2 weeks | Fruit and vegetable intake*   - Primary | - Affordability: Free access to messages | Participants must have a mobile phone with an internet connection |
| **(Chen et al., 2017; Chen et al., 2019), USA** | Social cognitive theory | Wearables, online program, SMS | i Start Smart for Teens educational program  *1. used a wearable sensor (Fitbit Flex) for 6 months*  *2. reviewed eight online educational modules for three months,*  *3. received tailored, biweekly text messages for three months.* | Provided with pedometer, blank food and activity diary, access to online program with general health information | 6 months | CHIS questions: Food consumption (SSB, FV, breakfast and fast food)   - Secondary | - Affordability: Free access to online program - Technology personalization: Tailored text messages, individualised goals | Participants needed access to a computer and internet |
| **(Doyle et al., 2008), USA** | Cognitive behavioural approach | Online program | Student Bodies 2 (SB2) cognitive-behavioural program  16-week internet program  *1. wear pedometers throughout intervention phase*  *2. weekly education content for cognitive exercises*  *3. email weekly newsletter containing individualised feedback* | Received coloured handouts containing basic information on nutrition and physical activity but were not given specific instructions on behaviour modification | 16 weeks | Eating Disorder Examination Questionnaire: dietary diagnostic behaviours   - Primary | - Usability: The adolescent and parent received a brief orientation to the Internet program - Perceived usefulness: Internet interface with gender-specific content - Technology personalization: Individualised feedback on program activities - Interactivity: Have forum for discussion group - Accessibility: May use school/ public computer to access the program - Affordability: Free program | Accessibility: Need a home computer and internet to access the app, exclude who were incapable of reading at the grade 5 level or above  Algorithmic bias: N/A |
| **(Ghadam et al., 2023), Iran** | Game-based education | App | Randomized clinical trial  14-week predefined training through digital games: *Educational information about nutritional iron conveyed to gamers during limited game time.* | The control group received basic nutrition education through PowerPoint and pamphlets. | 14 weeks | Diet knowledge, attitude, practice of iron intake: Level of ferritin *, transferrin, iron, TIBC, Iron, score of attitude, practice   - NR | - Usability: Received the predefined training - Affordability: Free use of game | those who can use, and have access to, a smartphone or tablet |
| **(Heikkila et al., 2019), Finland** | Increase self-efficacy and establish behavioural change to achieve healthy eating | App | Group EDU and group EDU  +APP  Group EDU: 3 x 90min sessions fortnightly, nutrition knowledge questionnaire and three-day food diary  Group EDU + APP: 3 x 90min sessions fortnightly, nutrition knowledge questionnaire and three-day photo and nutritional network application MealLogger® with their smartphones for four days after each session diary and written feedback from the nutritionist | * Just education  * The aim of the education sessions was to increase the nutrition knowledge among the athletes | 17 weeks | Validated nutrition knowledge questionnaire for young endurance athletes: Eating behaviours   - Primary | - Affordability: Free access to mobile app - Technology personalisation: Personal feedback upon quality of meals |  |
| **(Heinicke et al., 2007), Australia** | Cognitive behavioural principle- self-awareness and positive behaviour establishment | Online program | My Body, My Life Program  *1. 6-weekly training sessions*  *2. Weekly 90-minute group sessions conducted online* | Delayed treatment control group  *After 6–8 weeks, participants in the delayed treatment control were sent a questionnaire and involvement in the randomised control trial was ceased and interested girls then participated in the intervention | 6 weeks | Dutch Eating Behaviour Questionnaire Restraint subscale: Eating behaviours*   - Secondary | - Usability: Intervention participants were sent the program manual and asked to check access to the program site before their first group and to inform their group leader of any problems. After our pilot program, a trouble-shooting CD was created and sent to participants to ensure they had essential software which greatly reduced technical difficulties - Perceived usefulness: A qualitative evaluation assessing participant’s thoughts on the internet delivery mode, comfort and acceptance of the internet approach, and practicality of the approach for reaching participants who might otherwise not be able to access traditional forms of therapy was used to gather evidence about the efficacy of internet-based treatments and their applicability to the adolescent population. - Interactivity: Weekly discussion group, discussion board - Affordability: Free access to online program | Participants in both groups who were randomised but then did not meet inclusion criteria (e.g., did not have internet access or were receiving alternative treatment), were excluded |
| **(Jones et al., 2008), USA** | Cognitive behavioural therapy | Online program | StudentBodies2-BED  I: 16-week internet-facilitated intervention  *1. weekly education content, research assistant-moderated discussion group*  *2. weekly recap educational letter*  Waitlist C: Begun the program 9-months later | Waitlist | 16 weeks | Eating behaviour Inventory: Binge eating behaviour*, dietary fat, sugar intake   - Secondary | - Interactivity: Discussion group among participants - Affordability: Free access to website |  |
| **(Lana et al., 2014), Spain and Mexico** | A.S.E. model, Prochaska and DiClemente’s Transtheoretical model | Online program | One control group (C) and 2 experimental groups (1E, 2E)  C: Usual care  1E: Exclusively online intervention  2E: Online intervention and encouraging text messages  *1.Online educational sections targeting risky behaviours,* also provided dietetic advice, homemade recipes, 24-hour food recall, peer-starred educational videos, forums, chat lines, documents, web link and educational games.  *2. Text messages (if they provided a cellphone) to encourage compliance with healthy behaviours*  *Intervention group 1: Just received online information*  *Intervention group 2: Received online information and text messages* | Just received the questionnaire | 9 months (academic year) | Validated Food Frequency Questionnaire in Spain: Fruit, red meat, vegetables, legumes, pastries and cream intake.   - Primary (as part of total behavioural risk score) | - Interactivity: Forums and chat lines - Affordability: Free access to all sections of the website - Technology personalisation: Dietetic advice after analysing recipes |  |
| **(Likhitweerawong et al., 2021), Thailand** | Technology-based healthy eating habits improving- improve adherence, promote motivation | App | Two arm parallel RCT: standard care (SC) and SC + intervention  SC: Standard treatment of obesity  SC + I: Standard care combined with the OBEST application  *1.Set intake target + reward*  *2. Regular educational information about different healthy food*  *3. Regular reminding messaging every fortnight* | Received the standard treatment of obesity, followed by the Thai clinical practice guideline for childhood obesity treatment and prevention | 6 months (baseline to follow-up) | Having 3 meals/day  Eating fruits and vegetables every day  Milk consumption every day  Less frequent consumption of snack  Less frequent consumption of sugar-sweetened beverages  Less frequent consumption of fast food*   - Secondary | - Usability: The participants in this group were provided a two-hour workshop to learn the application’s instructions, the recommended diet, calories, portion sizes of food, and nutritional facts - Perceived usefulness: Designed by paediatricians to be suitable for children/ adolescents personalised target in the app according to own data - Affordability: Free of charge application | Accessibility: Need android device, exclude if having an intellectual disability or requiring special educational needs  Algorithmic bias: N/A |
| **(Manzano-Felipe et al., 2024), Spain** | Theory of change processes, make process rewarding, behavioural strategies | App | mHealth RCT  I: in app read educational materials, answer questionnaire questions, professional feedback upon progress  C: Usual intervention on healthy habits | Received regular school-based health education which is part of Andalusian’s public health interventions | 8 months | KidMed questionnaire: Adherence to Mediterranean diet   - Secondary | - Digital literacy: Application designed for adolescents' knowledge level - Affordability: Free of charge application - Technology personalisation: Got standard feedback for their progress | Accessibility: Need mobile device with Android operating system, exclude student with visual/ hearing disability  Algorithmic bias: N/A |
| **(Nollen et al., 2014), USA** | Mobile technology-based behaviour changing | Online program | Mobile technology after school program  I: Mobile intervention prompted real-time goal setting, self-monitoring, feedback and positive reinforcement to the target behaviours  C: Received same content written in a manual with no promoting  *1. I:In 12 weeks targeted fruits/vegetables, sugar-sweetened beverages and screen time. Real-time goal setting prompted, tips provided.*  *2. I+C: three 4-week modules on fruits/vegetables, sugar-sweetened beverages and screen time.* | Controls received the same content in a written manual but no prompting. | 12 weeks | Standardised 24-hour dietary recall multiple-pass method: Fruit and vegetable intake, SSB intake   - Secondary | - Data poverty and information asymmetry: Target economically disadvantaged neighbourhoods - Usability: Manuals were composed of screen shots from each respective module and were identical in content to MT. Unlike MT, the control condition relied on girls to initiate goal setting, planning, and self-monitoring and did not include action cues or a reward system. - Accessibility: Provided with handheld computer - Affordability: Free access to the program on mobile phones and participants were given a MyPal A626 handheld computer - Technology personalisation: Own goal setting |  |
| **(Patrick et al., 2013), USA** | Behavoural determinants model, transtheoretical model of behavioural change | SMS, online program, phone calls, email | 4 arm RCT: I1, I2, I3 and UC  UC: Printed materials and three initial group sessions  I1: Website only  I2: Website, monthly group sessions and follow-up calls  I3: Website and SMS  *1. All participants wear pedometer and do regular weigh in.*  *2. Web: Weekly educational sessions, tutorials + skill building exercises, check-in emails, monthly mailed tips sheet, feedback provided on progress*  *3. Monthly sessions+ follow-up call: monthly 90 min adolescents +parents group sessions, bimonthly health counsellor phone calls*  *4. SMS: weekly >=3 text messages on intervention goals and behavioural strategies, case manager communication* | UC participants were given printed materials and encouraged to attend three initial group sessions | 12 months | Self-administered food frequency questionnaire for adolescent: Diet fat behaviour, fruit and vegetables intake   - Primary | - Data poverty and information asymmetry: Economically and ethnically diverse population - Usability: SMS supporting intervention goals and behavioural strategies and communicated via SMS with a case manager. During the development phase of the intervention, content was piloted and revised after input from a diverse group of adolescents regarding reading level, understanding of concepts, ability to hold their attention, and usability of information. - Perceived usefulness: Intervention has been designed and tested for - Digital literacy: Intervention has been tested to ensure understanding of concepts adolescent's usefulness - Accessibility: allow access to internet at school - Affordability: Free access to the website | Other inclusion criteria for both teens and parents included access to the Internet at home, work, or school for both parent and teen; having a functioning telephone; ability to speak and read English (for adolescent) or English or Spanish (for the parent); and willingness to participate in online activities and attend monthly group sessions |
| **(Shatwan et al., 2023), Saudi Arabia** | Smartphone-based healthy diet habit promotion | App | MyPlate  I: 6-week app program, instructional brochure, goal setting, app notifications and SMS reminders.  C: Pre and post interview questions | No procedures | 6 weeks | Validated food-frequency questionnaire: Fruit intake score,  vegetable intake score   - Primary | - Usability: Adolescents in the intervention group were divided into 11 smaller groups, each containing 5 participants, to explain the app.   The research team was available to assist participants at any time during the study period.   - Affordability: Free to download application - Technology personalization: Choose own 3 goals out of 7 provided | Accessibility: Need access to personal or parent's smartphone, exclude adolescents whose health not in good condition  Algorithmic bias: N/A |
| **(Sivasamy, 2020), India** | Mobile technology based dietary instructions delivery | App | Double-blinded RCT  I: Tailored dietary instructions via an app, motivational messages and questionnaires, questions to be asked through messaging  C: Standard one to one dietary instructions | Standard one-to-one dietary instructions once at start of study | 4 weeks | Nizel and Papas criteria: Sweet score   - Primary | - Affordability: free to download application - Technology personalization: tailored instructions | Accessibility: need access to own smartphone and be active on the application, exclude subjects with special diet requirement  Algorithmic bias:  N/A |
| **(Tugault-Lafleur et al., 2023), Canada** | Healthy behaviours promotion | App | Aim2Be intervention  I: Aim2Be program with a live coach for six months, health coach tailored message + optional scheduled or unscheduled text support+ optional web-based appointment  Waitlist C: Aim2Be with no live coach, accessed after three months | Waitlist control, access to app not till 3 months after, received a brochure with Canadian health recommendations about PA, diet, screen time, and sleeping habits | 3 months | Mean total daily calories  Vegetable and fruit servings  Percentage of daily calories from saturated fats  Total amount from solid fats (saturated fats and trans fats)  Total daily intakes of unsaturated fats (in g)  Total fibres (in g)  Total sugars (in g)  Percentage of daily calories from discretionary foods (foods not part of the 4 “core” food groups in the 2007 Canada’s Food Guide)  Mean daily calories from sugary beverages (including and excluding 100% fruit juice)   - Secondary | - Perceived usefulness: Application pilot-tested for features getting higher engagement and better results, tailored messages from health coach - Interactivity: Users were able to interact with others through moderated social wall - Digital literacy: Designed different versions to suit level of literacy (Preteen: 10-13y, Teen: 14-17y) - Affordability: application Free to download - Technology personalisation: App designed for paediatric obesity management, have 3 versions targeting different age range | Accessibility: Need a home computer and internet to access the app, exclude who were incapable of reading at the grade 5 level or above  Algorithmic bias: N/A |
| **(van Egmond-Fröhlich et al., 2006), Germany** | Modular consultation guideline | Phone calls | Multi-centre RCT  I: Regular monthly outpatient consultations by the primary care physicians + telephone consultation  C: Standard care | Standard care | 13.5 months | Nutrition behaviour  ± Extras  ± Oils/Fats  ± Meat/Fish/Eggs  ± Milk/Dairy Products  ± Vegetables  ± Fruit  ± Cereal Products   - Secondary | - Affordability: Free of charge telephone consultation | Usability: Excludes disability or language barrier that precludes group training.  Algorithmic bias: N/A |
| **(Vermeiren et al., 2021), Belgium** | Self-control training | Online program | Double-blinded multi-centre RCT  Inpatient intervention group: 12 months intense Multidisciplinary obesity treatment (MOT)+ 6 months booster MOT sessions  Inpatient control group: 3.5 months self-control/ sham training after 10.5 months intervention group started MOT  Outpatient intervention group: 12 months intense MOT established by dietitians  Outpatient control group: 3.5 months self-control/ sham training after 4.5 months intervention group started MOT  MOT  *1. implement healthy diet. Individual psychological+ contextual support*  *2. Caregivers involved in healthy lifestyle education*  Self-control training  *1. 6 sessions every fortnight+ 8 sessions every week : Computer-based attention+ inhibition training on attention on healthy food and avoid unhealthy food*  *2. Caregivers involved in healthy lifestyle education* | Sham training | 12 months | Dutch Eataing Behaviour Questionnaire: External eating, motional eating   - Secondary | - Affordability: Free access to online training |  |
| **(Wambach et al.), USA** | Mobile technologies supported health behaviours change promotion | Online program,  SMS | Longitudinal RCT  I: 12-week intervention: nine multi-media educational modules, web-based information, daily text messages, weekly virtual home visit and peer support groups and online games  C: Received usual care | Received standard health care from their prenatal providers during clinic visits on healthy lifestyle, breastfeeding and postpartum lactation support through hospital “warm lines” and paediatric providers. | 12 weeks | Automated Self-Administered 24-hour Recall (version 2016 and 2020): Diet/24-hour food recall   - Secondary | - Usability: Given technology use and support-access instructions and participants were given nine multi-media educational modules were preloaded on the tablet, including links to web-based information/videos and interactive games/apps - Perceived usefulness: Adolescents prefer in-home research involvement --> designed to base at home - Accessibility: Participants were lent an iPad mini (32 GB) with a 6 GB data plan - Affordability: Used low-cost mobile technology to cover underserved teenagers | Algorithmic bias: N/A |

C: control; FV: fruit and vegetable; I: intervention; N/A: Not applicable; NR: Not reported; SMS: short message service; SSB: sugar sweetened beverage; UC: usual care; * significant change

**Table S11.** **Risk of bias of included randomized controlled trials (n=19) using the revised Cochrane risk-of-bias tool for randomized trials (RoB 2)**

| **Study** | **Randomization process** | | | | **Deviations from the intended interventions** | | | | | | | | **Missing outcome data** | | | | | **Measurement of the outcome** | | | | | | **Selection of the reported result** | | | | |
| --- | --- | --- | --- | --- | --- | --- | --- | --- | --- | --- | --- | --- | --- | --- | --- | --- | --- | --- | --- | --- | --- | --- | --- | --- | --- | --- | --- | --- |
| **First author, Year, Country** | **Allocation sequence** | **Allocation concealment** | **Baseline difference between groups** | **Risk-of-bias judgement** | **Participant blinding** | **Personnel blinding** | **Protocol deviations** | **Protocol deviations impact on outcome** | **Protocol deviations balanced between groups** | **Appropriate analysis** | **Impact of non-intention to treat analysis** | **Risk-of-bias judgement** | **Outcome data available for nearly all participants** | **Result not biased by mussing outcome data** | **Missingness dependent on true value** | **Likely that missingness dependent on true value** | **Risk-of-bias judgement** | **Outcome assessment appropriate** | **Outcome measurement differed between groups** | **Outcome assessors aware of participant intervention** | **Outcome influenced by knowledge of intervention received** | **Assessment of outcome influenced by knowledge of intervention** | **Risk-of-bias judgement** | **Follow a pre-specified analysis plan** | **Selective reporting of outcome within the outcome domain** | **Selective reporting of outcome based on multiple analyses of data** | **Risk-of-bias judgement** | **Overall risk of bias judgement** |
| Wambach et al. (2022) US | PY | Yes | No | **LR** | Yes | Yes | No | N/A | N/A | Yes | N/A | **LR** | No | No | PY | PN | **SC** | No | No | Yes | PY | PY | **HR** | Yes | No | No | **LR** | **HR** |
| Bjerregaard et al. (2024) Denmark | Yes | Yes | No | **LR** | Yes | PY | N/A | N/A | N/A | Yes | N/A | **LR** | No | Yes | N/A | N/A | **LR** | No | No | Yes | PY | PY | **HR** | Yes | No | No | **LR** | **HR** |
| Manzano-Felipe et al. (2024) Spain | Yes | Yes | No | **LR** | Yes | Yes | No | N/A | N/A | PY | N/A | **LR** | Yes | N/A | N/A | N/A | **LR** | No | No | NI | PY | PN | **SC** | NI | No | No | **SC** | **HR** |
| Likhitweerawong et al 2021 Thailand | Yes | Yes | No | **LR** | Yes | Yes | PN | N/A | N/A | Yes | N/A | **LR** | Yes | N/A | N/A | N/A | **LR** | No | No | No | PN | PN | **LR** | Yes | No | No | **LR** | **LR** |
| van Egmond-Fröhlich et al. (2006) Germany | NI | NI | NI | **SC** | Yes | PY | NI | N/A | N/A | Yes | N/A | **SC** | No | No | PY | PN | **SC** | No | PN | NI | PY | PN | **SC** | NI | NI | NI | **SC** | **HR** |
| Shatwan et al. (2023) Saudi Arabia | Yes | Yes | No | **LR** | Yes | Yes | PN | N/A | N/A | PY | N/A | **LR** | NI | PY | N/A | N/A | **LR** | No | No | Yes | PY | PN | **SC** | Yes | No | No | **LR** | **SC** |
| Tugault-Lafleur et al. (2022) Canada | Yes | Yes | No | **LR** | Yes | Yes | No | N/A | N/A | Yes | N/A | **LR** | Yes | N/A | N/A | N/A | **LR** | No | No | Yes | PY | PN | **SC** | Yes | No | No | **LR** | **SC** |
| Doyle et al (2008) USA | Yes | PY | Yes | **SC** | Yes | Yes | PN | N/A | N/A | Yes | N/A | **SC** | No | No | PY | PY | **HR** | No | No | NI | PY | PN | **SC** | NI | No | No | **SC** | **HR** |
| Ghadam et al. (2022) Iran | NI | PY | No | **LR** | Yes | Yes | PN | N/A | N/A | PY | N/A | **LR** | No | No | PY | PN | **SC** | No | No | NI | PN | PN | **LR** | Yes | No | No | **LR** | **SC** |
| Lana et al. (2013) Spain and Mexico | Yes | PY | Yes | **SC** | Yes | PY | PN | N/A | N/A | PY | N/A | **LR** | No | No | NI | NI | **HR** | No | No | Yes | PN | N/A | **LR** | Yes | No | No | **LR** | **HR** |
| Vermeiren et al (2021) Belgium | Yes | Yes | No | **LR** | Yes | Yes | PN | N/A | N/A | PY | N/A | **LR** | No | No | PY | PY | **HR** | No | No | Yes | PN | N/A | **LR** | Yes | No | No | **LR** | **HR** |
| Heinicke et al. (2007) Australia | Yes | Yes | No | **LR** | Yes | Yes | PN | N/A | N/A | Yes | N/A | **LR** | No | Yes | N/A | N/A | **LR** | No | No | Yes | PN | N/A | **LR** | Yes | No | No | **LR** | **LR** |
| Nollen et al. (2014) America | PY | NI | No | **SC** | Yes | Yes | PN | N/A | N/A | PY | N/A | **LR** | Yes | Yes | N/A | N/A | **LR** | No | No | NI | PN | N/A | **LR** | Yes | No | No | **LR** | **SC** |
| Patrick et al. (2013) America | NI | NI | No | **SC** | Yes | Yes | PN | N/A | N/A | Yes | N/A | **LR** | No | Yes | N/A | N/A | **LR** | No | No | Yes | PN | N/A | **LR** | Yes | No | No | **LR** | **SC** |
| Carfora et al. (2016) Italy | Yes | PY | No | **LR** | Yes | PN | N/A | N/A | N/A | Yes | N/A | **LR** | No | Yes | N/A | N/A | **LR** | No | No | Yes | PN | N/A | **LR** | Yes | No | No | **LR** | **LR** |
| Jones et al. (2008) America | Yes | NI | No | **SC** | No | No | PN | N/A | N/A | PY | N/A | **LR** | No | No | PN | PN | **LR** | No | No | No | N/A | N/A | **LR** | PY | No | Yes | **HR** | **HR** |
| Chen et al. (2017) AND Chen et al. (2019) America | Yes | Yes | No | **LR** | No | No | PN | N/A | N/A | Yes | N/A | **LR** | Yes | Yes | PN | PN | **LR** | No | No | No | N/A | N/A | **LR** | Yes | No | No | **LR** | **LR** |
| Heikkilä et al. (2019) Finland | Yes | PY | No | **LR** | Yes | Yes | PN | N/A | N/A | PY | N/A | **LR** | No | PN | PY | PN | **SC** | No | No | NI | PN | N/A | **LR** | Yes | No | No | **LR** | **SC** |
| Sivasamy et al., (2020) | Yes | Yes | No | **LR** | PY | PY | No | N/A | N/A | NI | PN | **SC** | No | No | PY | NI | **HR** | No | No | No | N/A | N/A | **LR** | Yes | No | No | **LR** | **HR** |
